# Harnessing Proton-Coupled Electron Transfer for Hydrogenation of Aza-Arenes: Photochemistry of Quinoxaline Derivatives in Methanol

**DOI:** 10.1021/acs.jpca.3c05077

**Published:** 2023-10-16

**Authors:** Olaf W. Morawski, Paweł Gawryś, Andrzej L. Sobolewski

**Affiliations:** Institute of Physics, Polish Academy of Sciences, Al. Lotników 32/46, 02-668 Warsaw, Poland

## Abstract

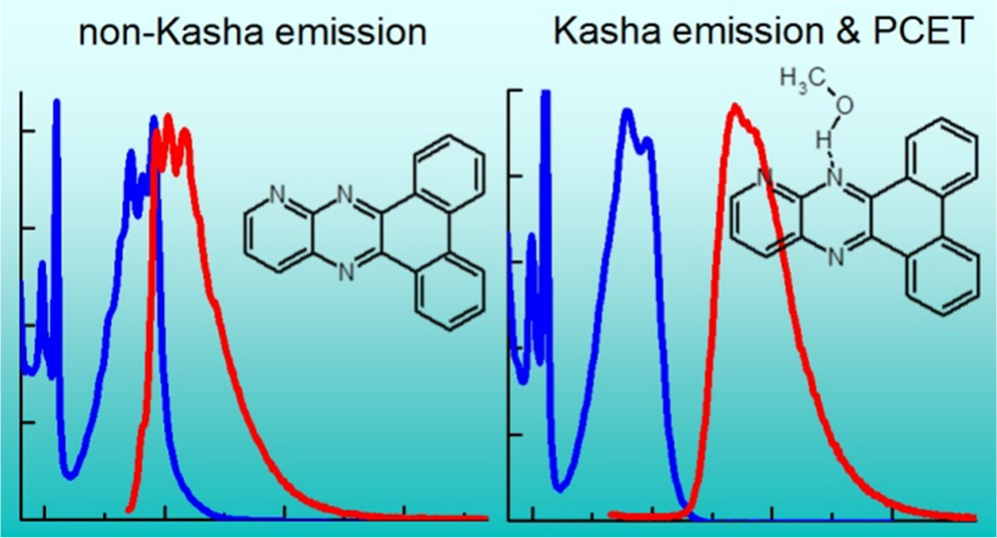

Three quinoxaline derivatives are investigated both experimentally and theoretically to assess their ability for the methanol oxidation and harvesting of hydrogen. In inert solvents, the nonplanar compounds exhibit very weak fluorescence from the lowest excited singlet state, whereas the planar and rigid chromophore emits non-Kasha fluorescence from the S_2_(ππ*) state despite the proximity of the S_1_(nπ*) state. In methanol, hydrogen-bonded complexes with solvent molecules are formed, and in all chromophores, the lowest singlet state is populated after excitation of the S_2_(ππ*) state. The switch from non-Kasha emission of the planar compound in inert solvents to regular emission in methanol is related to reduced symmetry of the hydrogen-bonded complex with methanol which results in effective mixing of ππ* and nπ* states and fast internal conversion to the lowest excited singlet state. The S_1_(nπ*) state of the hydrogen-bonded complex has charge-transfer character, and for all compounds in methanol, hydrogen transfer to the chromophore is observed. The chromophores retain the transferred hydrogen atoms, serving both as photocatalysts and as hydrogen storage materials. Undesired dark side reactions that occur are also discussed.

## Introduction

The conversion of solar energy into chemical energy represents a compelling concept for the production of clean and renewable fuels. However, it poses a significant challenge for photochemistry in terms of identifying suitable substrates, photocatalysts, and reactions. Fujishima and Honda made a breakthrough by demonstrating hydrogen generation from water in a photoelectrochemical cell.^1^ In their approach, holes created in the photoirradiated semiconductor photoanode are able to oxidize water molecules, resulting in the production of molecular hydrogen and molecular oxygen through a four-electron process.^2−4^ However, despite more than five decades of extensive research efforts in the field of photocatalytic water splitting, significant challenges persist. The stability of the materials and the low efficiency of photoelectrochemical cells remain key issues, rendering this technique inadequate for widespread industrial applications.^5,6^

An alternative approach to water splitting involves the direct photoinduced homolytic cleavage of H_2_O molecules into H• and OH• radicals using photocatalytically active chromophores.^7^ The strategy makes use of the fact that the basic sites of certain heteroaromatic systems, known as photobases, exhibit enhanced basicity in excited electronic states, which allows them to abstract hydrogen atoms from protic solvents, such as alcohol or water. The theoretical exploration of the excited electronic states of the hydrogen-bonded complex of oxotitanium porphyrin (TiOP) with a single water molecule has revealed that a hydrogen atom can be abstracted from H_2_O through a photoinduced electron–proton-transfer reaction in the TiOP–H_2_O complex. This reaction leads to the formation of the TiPOH• and OH• radicals, which can subsequently recombine to form closed-shell products, thus generating chemical energy carriers like H_2_ or H_2_O_2_.^7^ The experimental verification of this concept has been achieved using visible-light-absorbing oxotitanium tetraphenylporphyrin^8^ and titanyl phthalocyanine,^9^ where the generation of hydroxyl radicals has been observed. The mechanism of direct homolytic water splitting also has been confirmed in hydrogen-bonded clusters of pyridine (Py) and water under supersonic expansion conditions, with spectroscopic observation of PyH• radicals.^10^ For a derivative of heptazine, the photoinduced hydrogen transfer from water to the heptazine core has been demonstrated with OH• detection and isotope-dependent luminescence quenching of the chromophore in water.^11^ It has also been shown that the barrier for the photoinduced abstraction of a H atom from the water molecule is raised (lowered) by electron-donating (electron-withdrawing) substituents.^12^ The highly reactive hydroxyl radicals generated by water oxidation can recombine with the reduced chromophore radicals to yield photohydrates, and for some substituents, self-healing of the photocatalyst or recovery of its ability to perform another H-abstraction process has been suggested.^12^ Moreover, it has been predicted for heptazine (Hz) that the HzH radicals that are formed by the photoreduction of Hz can disproportionate to Hz and HzH_2_ in an exothermic reaction with a low reaction barrier, forming HzH_2_ that is a closed-shell and long-lived molecule.^13^ A report on photochemical hydrogen storage using hexaazatrinaphthylene (HATN) in methanol provides additional evidence for this mechanism and demonstrates that heterocyclic molecules with multiple nitrogen atoms can undergo photochemical dihydrogenation to form closed-shell molecular systems capable of long-term hydrogen storage.^14^ These findings highlight the potential of heterocyclic molecules with multiple nitrogen atoms as promising candidates for the research and development of photocatalytic hydrogen abstraction and storage systems.

**Chart 1:**
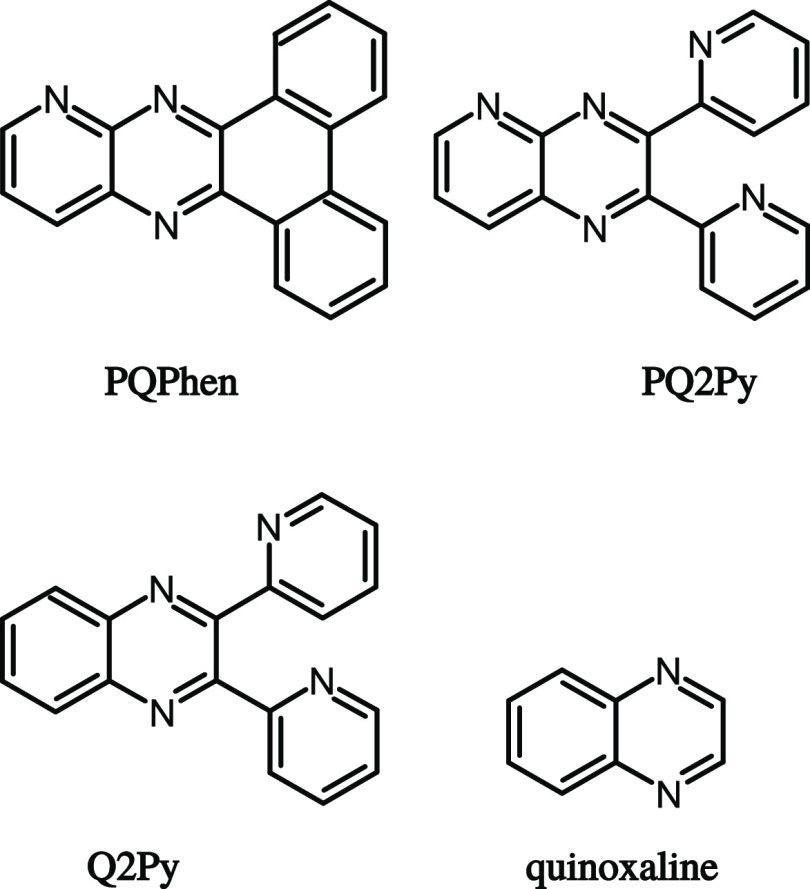
Molecular Structures of **PQPhen**, **PQ2Py, Q2Py**, and the Bare Quinoxaline

Quinoxalines and pyridopyrazines are aromatic heterocycles that can be easily synthesized and have diverse applications as organic semiconductors,^15^ dyes,^16^ electroluminescent materials,^17^ or bioactive materials in medical treatments of several illnesses.^18,19^ These heterocyclic molecules can also form hydrogen-bonded complexes with polar solvents, such as alcohols or water, expanding their potential applications. The presence of multiple nitrogen atoms in quinoxalines and pyridopyrazines provides an opportunity to investigate the formation of multiple hydrogenated products by the homolytic abstraction of hydrogen from methanol or water. For our combined experimental and theoretical studies, we have chosen three specific heterocyclic compounds with three, four, and five nitrogen atoms, respectively: dibenzo[*f*,*h*]pyrido[2,3-*b*]quinoxaline (**PQPhen**), 2,3-di(pyridin-2-yl) pyrido [2,3-*b*]pyrazine (**PQ2Py**), and 2,3-di(pyridin-2-yl)quinoxaline (**Q2Py**) as illustrated in Chart 1.

## Methods

Synthesis, experimental, and theoretical methods are described in the Supporting Information.

## Results and Discussion

### Ground-State Structures and Electrochemical Characterization

**Figure 1:**
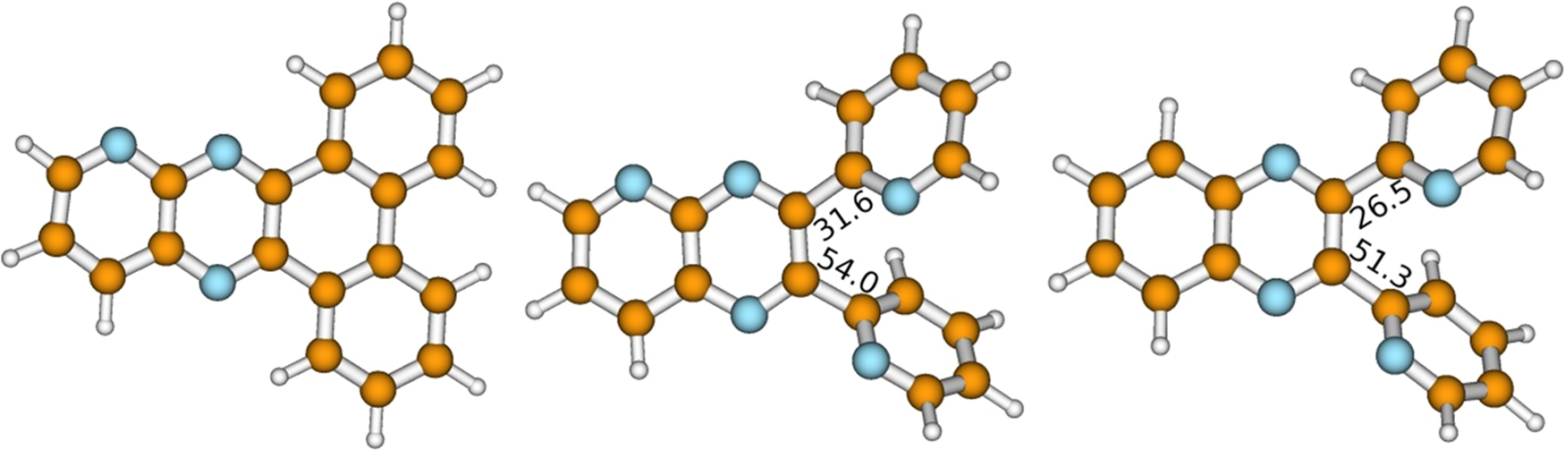
MP2/cc-pVDZ-optimized equilibrium molecular structures of **PQPhen**, **PQ2Py**, and **Q2Py**. The numbers denote values of NCCC and CCCC dihedral angles for **PQ2Py** and **Q2Py** and quantify the nonplanarity of these molecules.

The ground-state equilibrium geometries of the compounds determined with the second-order Møller–Plesset (MP2) method are presented in Figure 1. In contrast to planar and symmetric **PQPhen**, the structures of **PQ2Py** and **Q2Py** possess twisted pyridine rings and their symmetry is reduced. **PQPhen** forms a yellow powder, whereas the powders formed by **PQ2Py** and **Q2Py** are white. Cyclic voltammetry reveals that all compounds have similar redox properties and may undergo reversible electrochemical protonation in methanol (see Chapter 5.2 of the SI).

### Optical Spectra in *n*-Hexane

**Figure 2:**
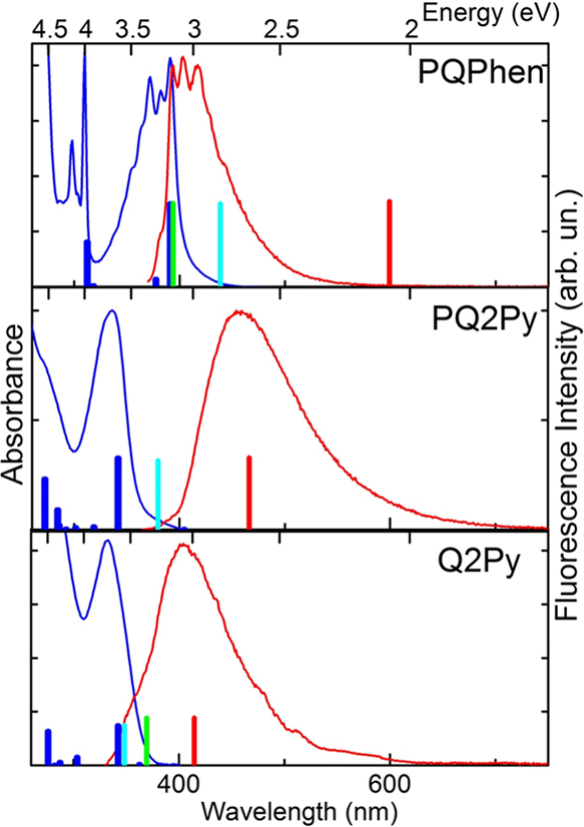
Normalized absorption (blue) and fluorescence spectra (red lines) of **PQPhen**, **PQ2Py**, and **Q2Py** in *n*-hexane. Vertical bars represent ADC(2)/cc-pVDZ-calculated transition energies and oscillator strengths of the absorption (blue, ππ* states; magenta, nπ* states) and fluorescence (green, ππ* states; red, nπ* states) bands, respectively. The heights of the bars for nπ* states are equal to those of ππ* states to make them visible. The (non-Kasha) fluorescence ππ* transitions for **PQPhen** and **Q2Py** were calculated with the C_s_ and *C*_*2*_ symmetry constrain, respectively, since only this symmetry allows geometry optimization of higher-lying ^1^ππ* state. Such a distinction by symmetry was not possible for the **PQ2Py** molecule, so only the S_1_ state of the ^1^nπ* orbital nature was optimized.

Although the three molecules of Chart 1 are known, their photophysics has not been extensively investigated yet.^20,21^ Our findings demonstrate that the optical properties of these compounds exhibit the typical characteristics of aromatic heterocycles, which are associated with their molecular structures. **PQPhen**, possessing the most extended π-electron system, exhibits an absorption band at 3.17 eV, while **PQ2Py** and **Q2Py** display absorption maxima at energies that are higher by 0.52 and 0.55 eV, respectively (see Figure 2). For **PQPhen**, the Stokes shift between the absorption and fluorescence spectra is minimal (258 cm^–1^) and vibronic progressions are visible in both spectra, indicating that the emission originates from the absorbing singlet state of ππ* character and that there is a negligible geometry change upon excitation of this state. In contrast, for **PQ2Py** and **Q2Py**, large Stokes shifts are observed, 7921 and 5216 cm^–1^, respectively (Table S4), which suggests that radiationless relaxation from the absorbing electronic state (S_2_) to the lowest excited singlet state (S_1_) takes place. The latter is fluorescent but not visible in the absorption spectrum. Hence, for **PQ2Py** and **Q2Py**, one may assign ππ* character to the S_2_ state and nπ* character to the weakly emissive S_1_ state. The assignment of S_1_(nπ*) and S_2_(ππ*) to the emissive and absorbing states accounts for the absence of the S_1_ band in the absorption spectrum, large Stokes shift, and the very low fluorescence quantum yield, Φ_F_, observed for **PQ2Py** and **Q2Py** (see Table 1). The rotation of the pyridine rings also may contribute to the considerable Stokes shift, very weak emission, and broad structureless band shapes of the luminescence spectra of these two compounds. Large amplitude twisting of the pyridine rings may lead to conical intersections of the potential energy surfaces (PESs) of the S_1_ and S_0_ states and thus fast internal conversion to the ground state. Indeed, low Φ_F_ and short fluorescence decay times are observed for P**Q2Py** and particularly for **Q2Py** (see Table 1). The much stronger and structured emission of **PQPhen**, in which two pyridine rings are replaced by phenanthrene, demonstrates that the rigidity of the molecular structure enhances the emission and preserves the vibronic structure of the optical spectra.

**Table 1:** Fluorescence Quantum Yield, Φ_F_; Decay Time, τ_F_; Radiative, *k*_r_; and Nonradiative Decay Rate, *k*_nr_, for **PQPhen**, **PQ2Py**, and **Q2Py** in Different Solvents

comp.	solvent	Φ_F_ [%]	τ_F_ [ps]	*k*_r_ [10^7^ s^–1^]	*k*_nr_ [10^9^ s^–1^]
**PQPhen**	*n*-hexane	2.7	180	15.0	5.4
	ACN	0.11	120	0.9	8.3
	MeOH	0.9	26	3.4	38.1
**PQ2PY**	*n*-hexane	0.25	200	1.25	5.0
	ACN	0.05	150	0.03	6.7
	MeOH	0.11	51	2.16	19.6
	H_2_O	0.09	550	0.16	1.82
**Q2PY**	*n*-hexane	0.012	46	0.26	21.7
	ACN	0.005	40	0.13	25.0
	MeOH	0.007	45	0.16	22.2
	H_2_O	0.022	206	0.11	4.85

### Optical Spectra in Other Solvents

**Figure 3:**
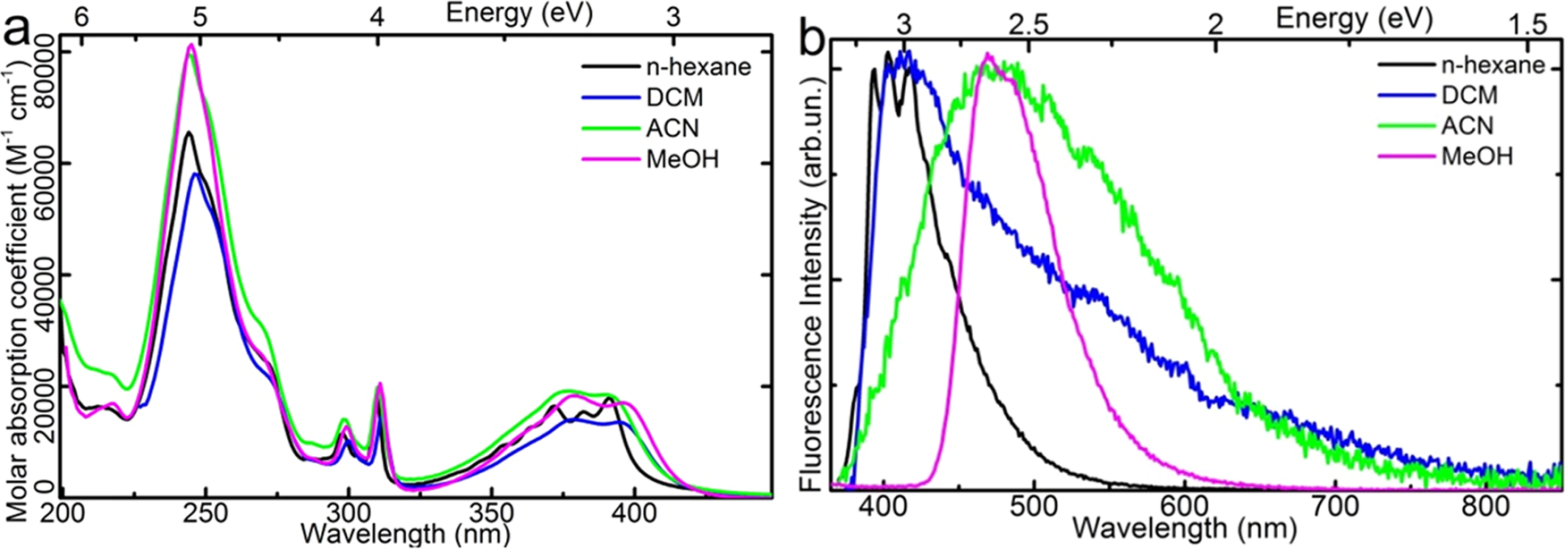
Absorption (a) and fluorescence spectra (b) of **PQPhen** in different solvents: *n*-hexane, dichloromethane, acetonitrile, and methanol. The fluorescence is recorded with excitation at 270 nm.

The absorption spectrum of **PQPhen** in solvents displays a weak bathochromic shift, indicating that the absorbing state is somewhat polar (Figure 3a). The largest shift occurs in methanol, even though it is less polar than acetonitrile (with dielectric constants 32.7 and 37.5, respectively). This observation suggests a solvent-specific interaction of **PQPhen** with protic methanol, presumably involving the formation of hydrogen-bonded complexes. Hydrogen bond decreases the energy of ππ* states and increases it for states of nπ* character; the change is correlated to electron density on the heteroatom and strength of the hydrogen bond.^22^ The fluorescence spectrum of **PQPhen** exhibits a much larger solvatochromic effect. In both acetonitrile and methanol, the maximum emission is shifted by 0.5 eV relative to *n*-hexane. This significant red shift of the emission spectrum, much larger than that observed for absorption, suggests that the emission of the hydrogen-bonded complex originates from either a highly polar state that experiences large solvatochromism or a different, lower-energy state not visible in the absorption spectrum. The decrease of the fluorescence quantum yield and shortening of the decay time observed for **PQPhen** in solvents other than *n*-hexane (Table 1) are typical for the optically forbidden nπ* states, suggesting that the emissive state possesses nπ* character. However, the S_1_(nπ*) assignment forces the assumption that the fluorescence in *n*-hexane originates from the S_2_(ππ*) state and is a non-Kasha emission (see the calculation and discussion below supporting this conclusion). The fluorescence spectrum in ACN is significantly broader than that in *n*-hexane or methanol. This phenomenon may be attributed to acetonitrile’s high polarity and its limited ability to form hydrogen bonds. Although its hydrogen-bond donor acidity parameter α is 0.19—much lower than methanol’s 0.93—ACN might influence the excited state of the solute and its emission through weak hydrogen-bonding interactions.^23^ Thus, the broad luminescence may consist of emissions from several HB solute–solvent complexes of different bond lengths and energy.

The absorption spectra of **PQ2Py** and **Q2Py** are structureless and show a small red shift in polar solvents (see Table S4 and Figure S1 in the SI). Similar small changes are observed for the fluorescence spectra of **PQ2Py** and **Q2Py** in various solvents, with the exception of the spectra in water, which display a blue shift with respect to those in DCM or ACN (see Figure S2). The blue shift in protic solvents is a characteristic of nπ* states in hydrogen-bonded complexes^22^ and provides further evidence that the fluorescence of these two compounds originates from a state of nπ* character. The substantial Stokes shift observed for **PQ2Py** and **Q2Py** in solvents decreases only in water (Table S4), where strong hydrogen bonds decrease the energy of absorbing S_2_(ππ*) states and increase the weakly emitting S_1_(nπ*). Fluorescence decay time of both compounds in water is longer, and radiationless pathways of relaxation are slowed down (Table 1). The fluorescence and fluorescence excitation spectra of powders, where the high concentration of molecules allows for the observation of the direct S_0_ → S_1_(nπ*) excitation, support the conclusion that the state of ^1^nπ* nature is below that of ^1^ππ* character (see Figure S3). The S_1_(nπ*) state is located above ^3^ππ* states (see Table 2) which facilitates intersystem crossing (ISC) to the triplet manifold (according to the El-Sayed rule^24^). ISC is another channel that contributes to a decrease of the fluorescence quantum yield and thus a shortened fluorescence decay time. Indeed, the fluorescence quantum yields of **PQ2Py** and **Q2Py** are lower than that of **PQPhen** (see Table 1). For **PQPhen** and **PQ2Py**, the shortest fluorescence decay times are observed in methanol, and the rates of radiationless deactivation are the highest in this solvent. This suggests that an additional nonradiative relaxation channel exists in this solvent.

**Table 2:** Vertical Absorption Energies (Δ*E*), Oscillator Strengths (*f*), and Dipole Moments (μ) of the Lowest Excited States of **PQPhen**, **PQ2Py**, and **Q2Py** Determined with the ADC(2)/cc-pVDZ Method at the MP2/cc-pVDZ Equilibrium Geometry of the Ground State

comp.	state	Δ*E*/eV	*f*	μ/Debye
PQPhen	S_0_	0.0		2.14
	^3^nπ*	2.932		1.09
	^3^ππ*	3.011		3.19
	^1^nπ*	3.226	0.0	1.20
	^1^ππ*	3.641	0.341	4.19
	^1^ππ*	3.754	0.031	12.23
	^1^ππ*	4.367	0.004	5.50
PQ2Py	S_0_	0.0		2.37
	^3^nπ*/^3^ππ*	3.127		1.20
	^3^ππ*	3.498		1.81
	^3^ππ*	3.565		4.11
	^1^nπ*/^1^ππ*	3.466	0.001	0.81
	^1^ππ*	4.030	0.308	3.61
	^1^nπ*/^1^ππ*	4.290	0.011	1.25
	^1^nπ*	4.515	0.008	3.37
Q2Py	S_0_	0.0		0.97
	^3^ππ*	3.618		2.20
	^3^ππ*	3.175		1.13
	^3^nπ*	3.459		1.22
	^1^nπ*	3.819	0.004	2.18
	^1^ππ*	4.021	0.168	0.64
	^1^ππ*	4.474	0.002	3.38
	^1^nπ*/^1^ππ*	4.484	0.034	1.98

### Computational Results for Isolated Chromophores

The vertical excitation energies of the three chromophores of Chart 1 computed with the ADC(2) method are listed in Table 2, and the molecular orbitals relevant for optical transitions are displayed in Table S1. Inspection of the orbitals reveals that in all systems, the lowest excited singlet state is mainly of nπ* character, while the orbital character of the lowest excited triplet state varies from pure nπ* in **PQPhen** via mixed nπ*/ππ* character in **PQ2Py** to pure ππ* character in **Q2Py**. Since the optical excitation of the ^1^nπ* state is essentially dipole forbidden due to the orthogonality of the n and π orbitals, the lowest absorption band observed in all compounds is assigned to the S_2_(ππ*) state. In nonplanar **PQ2Py** and **Q2Py**, the mixing of S_2_(ππ*) with S_1_(nπ*) by vibronic coupling is allowed and may be additionally facilitated by rotation of the pyridine rings. As a result, the S_2_(ππ*) → S_1_(nπ*) radiationless relaxation is fast and the fluorescence originates from the lowest excited singlet state.

Let us notice that the theory provides explanation for the unusual spectral behavior of the chromophores. For **Q2Py**, the theory predicts similar electric dipole moment, μ, for the S_0_, S_1_(nπ*), and S_2_(ππ*) states: 0.97, 2.18, and 0.64 D, and 5.79, 5.63, and 5.19 D for these states in an isolated molecule and in its complex with MeOH, respectively (Tables S1 and S3). The difference between the ground and excited electronic state dipole moment is small, below 1 D, whereas the molecular radius, *a*, is several angstroms large (>6 Ǻ), so the Lippert–Mataga solvatochromic slopes for absorption 2 μ_g_(μ_e_ – μ_g_)/*h*ca^3^ and emission 2 μ_e_(μ_e_ – μ_g_)/*h*ca^3^ are tiny.^31^ In result, one may expect negligible solvent polarity effect on emission or absorption spectra. This is indeed observed for **Q2Py** (Figures S1c and S2c) and the only exception, the hypsochromic shift of fluorescence spectrum in water, can be assigned to the increase of nπ* state energy in a hydrogen-bond complex with a highly protic water molecule. Also for **PQ2Py**, the calculated dipoles are small and hence solvent polarity plays a secondary role in spectral shifts for these molecule, and hydrogen bonding and excited-state relaxation are dominant factors for their nontypical solvatochromism. In **PQPhen**, the dipole moment of the S_2_(ππ*) state is bigger by 2 D than that of S_0_ (Table S1), and only for this molecule, one observes a solvatochromic shift of the fluorescence spectrum in polar solvents.

For **PQPhen** in *n*-hexane and DCM, anomalous non-Kasha^25,26^ luminescence from the S_2_(ππ*) state is observed. This is surprising, because the energy difference between the S_2_(ππ*) and S_1_(nπ*) states in **PQPhen** is only 0.4 eV, much less than the 1.74 eV reported for the S_2_–S_1_ energy gap in azulene, for example, which is the classic representative of non-Kasha fluorescence.^27,28^ The anomalous S_2_ emission in azulene is related to slow S_2_ → S_1_ internal conversion. According to the energy gap rule for radiationless transitions, the rate of internal conversion slows down with increasing energy separation between the states involved.^29^ Since **PQPhen** has S_2_(ππ*) and S_1_(nπ*) states separated by only 0.4 eV, fast S_2_ → S_1_ relaxation and ordinary emission would be expected. The lack of fast internal conversion from the ^1^ππ* state to the ^1^nπ* state in **PQPhen** can be related to the molecular structure of this compound. In planar and rigid **PQPhen**, the S_1_(nπ*)–S_2_(ππ*) mixing requires out-of-plane modes and, in particular, high-frequency C–H vibrations. The molecular structure of **PQPhen** is unusual: its 11 hydrogen atoms reside on the peripheral rings, which may explain why the effect of these vibrations on the mixing of the excited electronic states is small. For **PQPhen** in methanol, the solute–solvent molecule hydrogen-bonded complex has reduced symmetry and therefore S_1_(nπ*) fluorescence appears. These findings reveal the effect of the symmetry constraint on the vibronic relaxation from the S_2_ state to the S_1_ state in **PQPhen**. Let us note that a switching of the emissive state induced by intermolecular H bonding has been already reported for an azulene derivative.^30^ Our results for **PQPhen** document a similar process. A recent study on the effects of solvents on photophysical and photochemical pathways, conducted using ultrafast spectroscopy, provides insight into the mechanisms that link the rate constants of excited-state processes with the energy levels of ππ* and nπ* states.^32^ In hydrogen-bonded complexes, ππ* states are stabilized, while nπ* states are destabilized, leading to changes in the energy gap between them or even a potential reversal. Moreover, hydrogen bond may undergo strengthening and weakening in ππ* and nπ* states, respectively, thereby influencing hydrogen atom transfer.^33^ Additionally, the rate of internal conversion from S_2_(ππ*) to S_1_(nπ*) can increase, facilitating ordinary Kasha emission or the population of photochemically reactive states.^34^ This mechanism may influence the fluorescence switch observed in **PQPhen** in addition to the out-of-plane vibronic mixing discussed above.

### Computational Results for Complexes with Methanol

**Figure 4:**
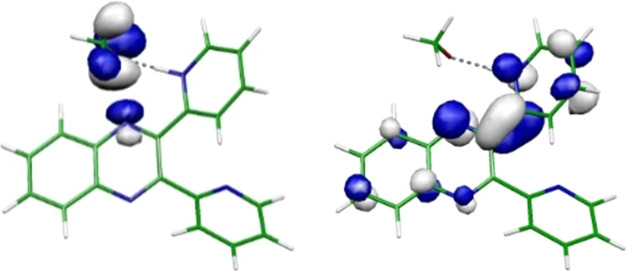
Natural transition orbitals (NTOs) determined at the optimized geometry of the S_1_(nπ*) state of the hydrogen-bonded complex of **Q2Py** with a methanol molecule: hole (left) and electron (right).

For each of the three chromophores, there exist several possibilities to form hydrogen-bonded complexes with a methanol molecule. The energetically most stable complexes are listed in Table S2. The complexation with the methanol molecule does not affect the orbital nature of the lowest excited singlet state of the systems. Like in the bare molecules, the S_1_ state of the complexes has nπ* character. The spectroscopic properties of the energetically most stable complexes with methanol computed at the ADC(2) level are listed in Table S3. ADC(2) optimization of the geometries of the complexes in the lowest excited singlet state (S_1_) allows the determination of the adiabatic excitation energies, the energies of vertical fluorescence, and the oscillator strengths (see Table S3). All transition energies and intensities are similar for the different conformers of a given chromophore with the exception of the **Q2Py-1** complex (**Q2PY** with one methanol molecule). This complex is not stable in the S_1_ state. The optimization of the equilibrium geometry of the S_1_ state results in spontaneous oxidation of the methanol molecule and the reduction of the nearby pyridine site. The photochemical reactivity of this complex can be related to the particular character of the n orbital of the S_1_(nπ*) state. In **Q2Py-1**, the n orbital is delocalized over the whole complex (see Table S3). In other words, the S_1_(nπ*) state of the complex exhibits significant charge transfer (CT) from methanol to the **Q2Py** chromophore. This provides the driving force for the photoredox reaction in this system. In the course of this reaction, the hole localizes on the methoxy moiety, while the proton is transferred to the pyridine site of **Q2Py** (see Figure 4). The energy profile of S_1_(nπ*) is barrierless along this proton-coupled electron-transfer (PCET) reaction path in this electronic state.

The lowest ^1^nπ* state of the complexes of **PQPhen** and **PQ2Py** with methanol also has partial CT character: upon optical excitation, part of the electron density is transferred from methanol to aromatic rings of the chromophores—compare the orbitals 76a and 83a for **PQPhen** or the orbitals 77a and 84a for **PQ2Py** (see Table S3). The effect is less pronounced than that for the orbitals 76*a*/77a and 84a of **Q2Py** (see Table S3), but clearly the charge is shifted onto chromophore rings, leaving a partial hole on the methanol molecule. Hence, the PCET reaction path is expected to exhibit a barrier in these complexes.

### Theoretical Exploration of the Kasha and Non-Kasha Photochemistry of the PQPhen–Methanol Complex

The case of **PQPhen**, showing non-Kasha florescence in *n*-hexane and in DCM, requires more extensive discussion of the close-lying S_1_(nπ*) and S_2_(ππ*) electronic states. Table 3 contains results obtained for the optimized geometry of the S_1_(nπ*) state of the molecular complex with methanol and for the optimized geometry of the S_2_(ππ*) state. In the latter case, it was necessary to impose the *C*_*s*_ symmetry constraint. In both cases, the CT character is clearly visible for the holes, as it was for the **Q2Py-1** complex. The locally excited (LE) S_2_ state has a substantial dipole moment, which explains the bathochromic shift of the **PQPhen** fluorescence in nonprotic solvents (Figure 3). The pronounced CT character of both states suggests that the PCET process may occur in the S_1_ state as well as in the S_2_ state.

**Table 3:**
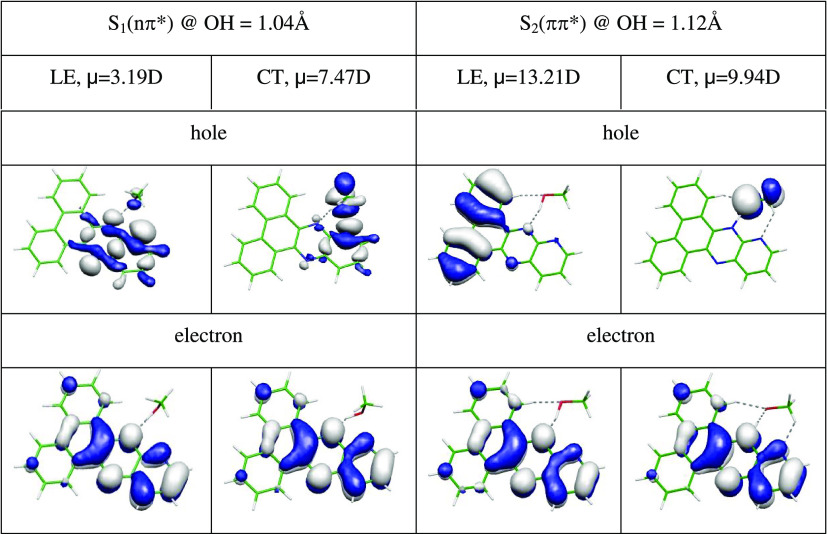
Natural Transition Orbitals (NTOs) Determined at the Optimized Geometries of the S_1_(nπ*) (Left) and S_2_(ππ*) (Right) States of the **PQPhen**–Methanol Complex

**Figure 5:**
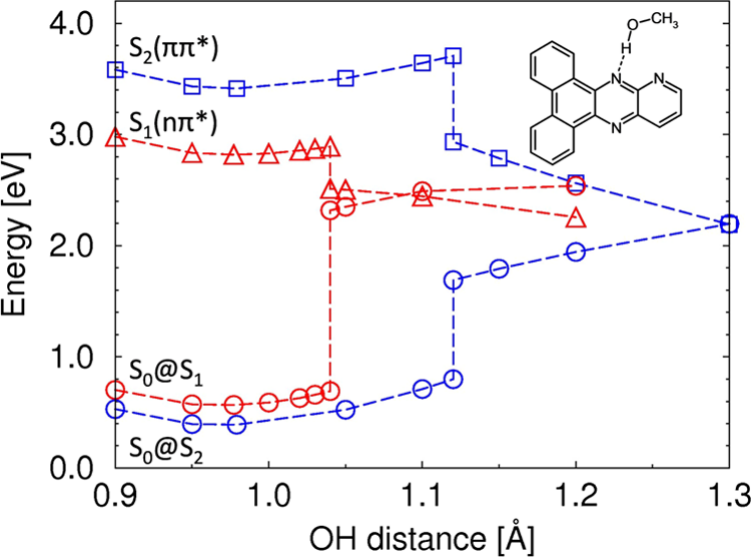
Energy profiles of minimum energy paths for hydrogen transfer from methanol to **PQPhen** in the S_1_(nπ*) (red triangles) and S_2_(ππ*) (blue squares) states of the **PQPhen** complex, calculated with the ADC(2)/cc-pVDZ method along the OH-stretching coordinate of the methanol molecule. Circles denote energies of the ground state computed at geometries optimized in excited states (S_0_ @ S_1_ and S_0_ @ S_2_, respectively).

To illustrate the mechanism of the PCET phenomenon, potential energy (PE) profiles determined for the S_1_ and S_2_ states of the P**Q2Phen-1** complex along the minimum energy path for H-atom transfer are presented in Figure 5. The PE profile of the S_1_(nπ*) state exhibits a small barrier of 0.037 eV (300 cm^–1^, ∼1 kcal/mol), which indicates rapid PCET in this state. The discontinuities of the PE profiles in Figure 5 indicate the existence of a second reaction coordinate and the formation of a hydrogen bond with the neighboring nitrogen atom. Further increase of the OH distance leads to further decrease of the PE and an S_1_–S_0_ energy crossing (i.e., a conical intersection). The energy profile of the S_2_(ππ*) state also possesses a local minimum in the vicinity of the ground-state equilibrium of the complex and exhibits a significantly higher barrier of 0.26 eV (2100 cm^–1^ or 6 kcal/mol) and therefore is less reactive with respect to PCET, although it should be kept in mind that the geometry optimization of the S_2_ state was possible only with the *C*_*s*_ symmetry constraint.

In the liquid phase, the reaction path and its energy barrier can be modified by interaction with polar solvent molecules. In particular, the dipole moment of the complex changes along the reaction coordinate. For the S_1_ state, the minimum of the energy is located at an OH distance of 0.963 Å and the dipole moment is 3.10 D. Upon elongation of the OH distance to 1.04 Å, the dipole moment increases slightly to 3.19 D. For the S_2_ state, the increase of the dipole moment is larger, from 12.79 to 13.21 D. The estimation of the decrease of the barrier due to the increase of the dipole moment along the reaction coordinate within the point-dipole approximation and a continuum model, as described in Section 2 of the SI, yields 1.6 and 30.0 meV for the S_1_ and S_2_ states, respectively. These effects are small compared with the barriers of 37 and 260 meV in the S_1_ and S_2_ states computed at the ADC(2) level. Another argument against an efficient PCET reaction in the S_2_ state is the finding that the release of the *C*_*s*_ symmetry constraint results in rapid relaxation to the S_1_ state, followed by a PCET process in this state.

### Hydrogenated Chromophores as Chemical Energy Storage Materials

Photochemically hydrogenated chromophores, such as **PQPhenH**• radicals, store chemical energy but are reactive and therefore are short-lived species. Their dihydrogenated closed-shell counterparts, such as **PQPhenH**_**2**_, are closed-shell systems and chemically stable. With the aid of suitable catalysts, the excess hydrogen atoms may be released or may engage in reactions with other molecules. For example, calculations performed at the DFT/D3-B3LYP/cc-pVDZ level indicate that the reaction between **PQPhenH**_**2**_ and molecular oxygen is exothermic, with an enthalpy change of −0.946 eV (−22 kcal/mol)—see Scheme SS1 in Section 4 of the SI.

### Spectroscopic Verification of the PCET Reactions

The theoretical predictions discussed above indicate the existence of a photoinduced PCET reaction in the *Q*-MeOH complexes, where *Q* stands for any of the three chromophores in Chart 1.
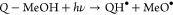
1

**Figure 6:**
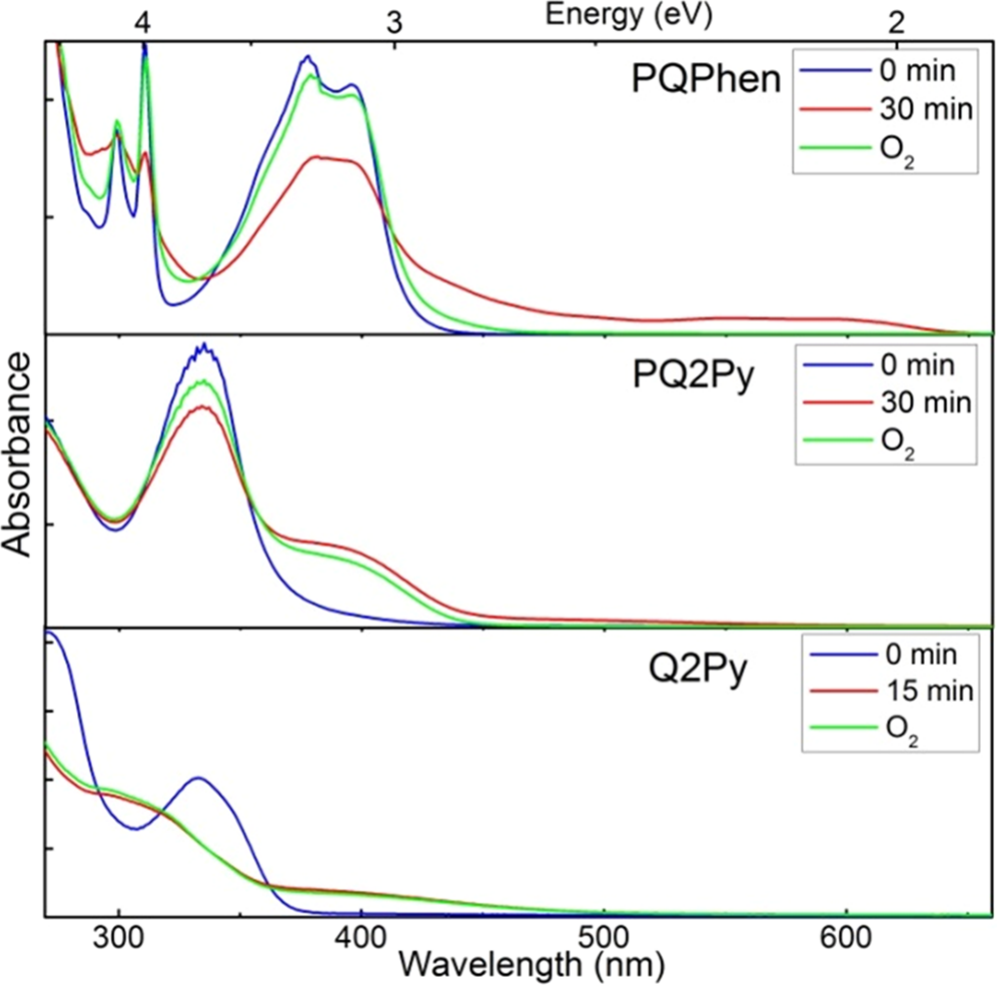
Absorption spectra of **PQPhen**, **PQ2Py**, and **Q2Py** in deaerated methanol before irradiation (blue), after irradiation (red), and after aeration (green). The solutions are photoirradiated at 405 (**PQPhen**), 375 (**PQ2Py**), and 277 nm (**Q2Py**).

**Figure 7:**
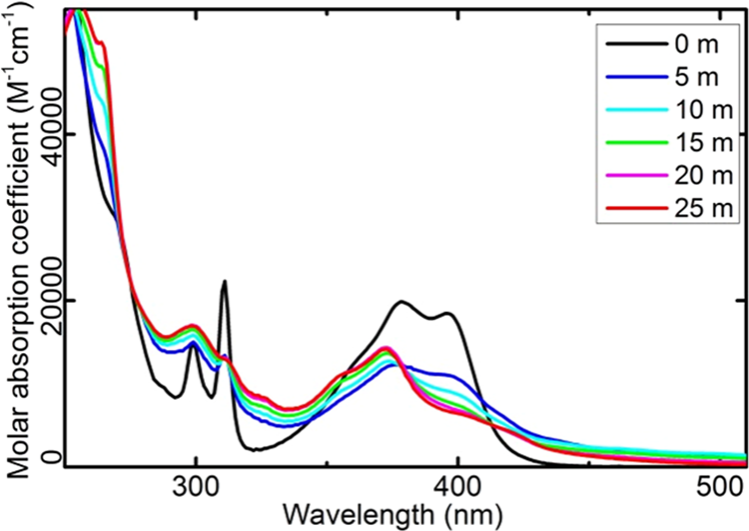
Effect of 405 nm photoirradiation on the absorption spectrum of **PQPhen** in methanol. The legend specifies the time of irradiation in minutes.

To verify these predictions experimentally, we conducted irradiation tests on chromophores dissolved in degassed methanol. After subjecting the methanol solution to 15 min of irradiation, noticeable changes in the absorption spectra were observed (see Figure 6). These alterations in the absorption spectra were accompanied by a change in the color of the solutions. The emergence of new absorption bands occurs at the expense of the original bands (see Figures 7 and S4). This clearly demonstrates the conversion of the substrate into photoproducts by a photochemical process. Moreover, it is evident that the absorption spectra of the photoproducts differ from those of the original compounds. The **PQPhen** photochemistry is reversible in the presence of molecular oxygen, while for **PQ2Py** and **Q2Py**, the photoinduced process is only partially reversible and the recovery of the original absorption spectrum is not possible (see Figure 6). This observation suggests that molecular oxygen may abstract hydrogen atoms from hydrogenated **PQPhen**, thereby recovering its original structure. Indeed, a second deaeration–photoirradiation cycle is feasible for **PQPhen** and results in similar changes of the absorption spectrum. This is not possible for **PQ2Py** and **Q2Py**, indicating that the hydrogenation reaction is accompanied by other photochemical processes and photodegradation of the chromophores cannot be excluded.

**Figure 8:**
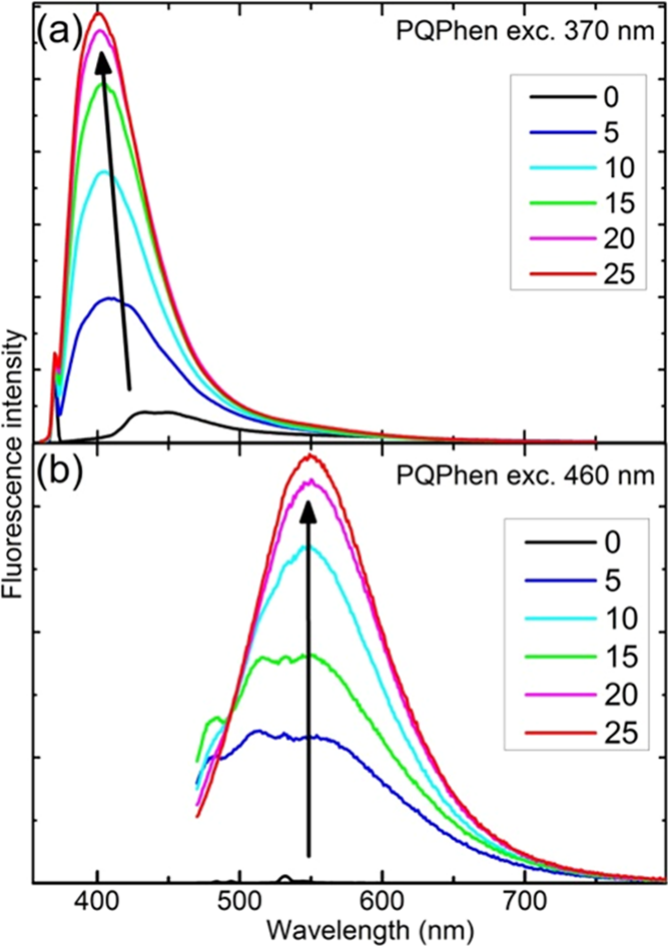
Effect of 405 nm irradiation on the fluorescence spectra of **PQPhen** in methanol. Upper (a) and lower (b) panels present spectra obtained with 370 and 460 nm excitation, respectively. The legend specifies time of irradiation in minutes. Arrows visualize the increase in fluorescence intensity with time of the irradiation. The very small black band in the lower panel represents a Raman line of the solvent in a spectrum recorded before irradiation (0 min).

The luminescence spectra also clearly indicate the formation of photoproducts. **PQPhen** exhibits two strongly emissive photoproducts (see Figure 8), **PQ2Py** one (see Figure S5) and **Q2Py** two (see Figures S6–S8). The fluorescence of the photoproducts exceeds the very weak emission of the original compounds, suggesting a transformation of the nature of the lowest emissive singlet state. Interestingly, the luminescence spectra of photoproducts are emitted not only at longer wavelengths than those of the substrates (see Figures 8b and S5 and S6) but also at shorter wavelengths (see Figures 8a and S6). One may hypothesize that this indicates a partial decomposition (S_1_ states usually have higher energies in small molecules and lower energies in larger ones) or a large change in the molecular structure of the chromophores and in the character of the lowest excited electronic state.

### Detection of Photoproducts with NMR and EPR

The photochemical processes also can be monitored with NMR. Upon irradiation, the intensity of the ^1^H lines of the original compound decreases, while new bands emerge in the spectra (see Figures S10–S12). The intensity of the new NMR lines increases with the time of photoirradiation, as illustrated in Figure S11. The new spectral features comprise not only narrow lines but also broad bands or fuzzy features emerging from the background, preventing an unambiguous analysis of the spectra.

The photoreaction (1) predicts the generation of radicals, and therefore, an attempt of the observation of radicals with the EPR technique was made. No signal was observed for the photoirradiated solutions. In contrast, for irradiated HATN, the radicals resulting from the hydrogenation of the HATN chromophore exhibited a weak EPR signal.^14^ The absence of an EPR signal in the irradiated solutions of the present chromophores can likely be attributed to fast radical recombination processes such as methoxylation of the hydrogenated chromophores or rapid formation of dihydrogenated chromophores by disproportionation of radicals.

**Figure 9:**
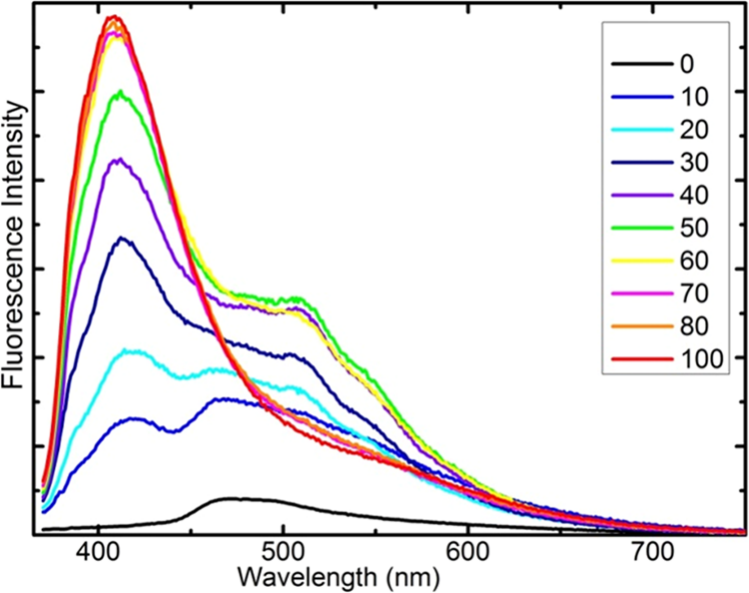
Effect of 405 nm irradiation on the fluorescence spectrum of a solution of **PQPhen** in methanol with 0.5 M coumarin as the radical scavenger. The legend specifies the time of photoirradiation. Spectra were recorded with excitation at 345 nm.

An attempt was made to detect methoxy radicals with coumarin as a radical scavenger. For **PQPhen** in methanol, the fluorescence spectra of the two **PQPhen** photoproducts, possessing maxima around 400 and 550 nm (see Figure 9), compete well with the fluorescence of methoxylated coumarin expected in the 450–550 nm spectral range (see Figure 9). This result, obtained with a high concentration of coumarin, 0.5 M, confirms the fast recombination of radicals postulated above and is another evidence that the recombination of radicals generated from **PQPhen** is faster than the recombination of the radicals generated from HATN, for which methoxylated coumarin spectra have been readily visible.^14^ For **PQ2Py** and **Q2Py**, the methoxylated coumarin fluorescence spectrum cannot be distinguished from the emission of the photoproducts, and the recombination of the generated radicals must be even faster.

### High-Resolution Mass Spectrometry of the Chromophores and Their Photoproducts

**Chart 2:**
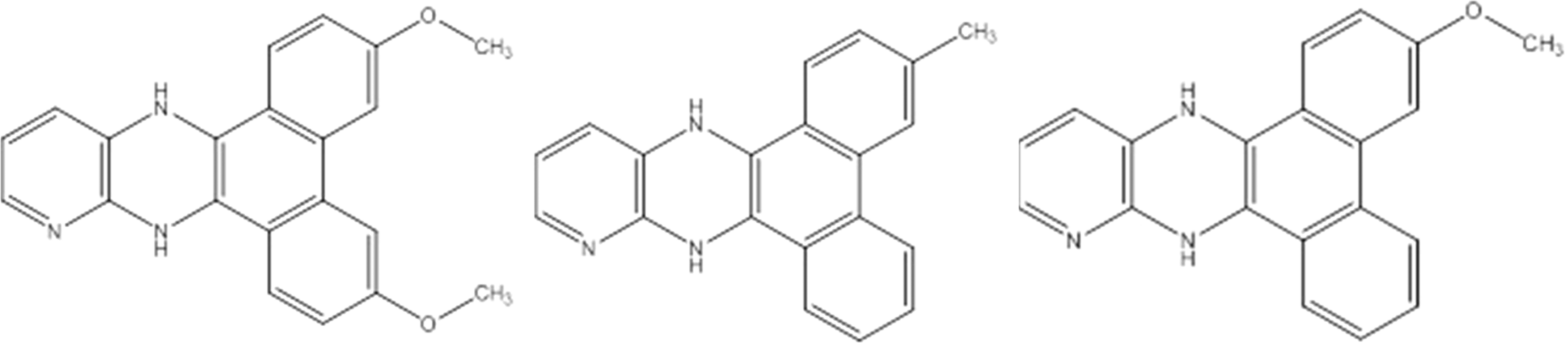
Proposed Molecular Structures of the Most Abundant **PQPhen** Photoproducts in Methanol

The absorption spectra of the irradiated solutions prove that the photoproducts are persistent in anaerobic solutions. To determine the masses and possible structures of the photochemical products, we performed high-resolution mass spectrometry under oxygen-free conditions. The measurements were performed before and after irradiation with positive and negative polarity (see Section 2 of the SI for details). Mass spectroscopy of **PQPhen** in methanol before irradiation reveals the protonated chromophore [**PQPhen** + H]^+^ with a mass to charge ratio *m*/*z* = 282.10 and the chromophore with sodium ion [**PQPhen** + Na]^+^ with a *m*/*z* = 304.08 (see Figure S13). The mass spectrum of the solution after five min of photoirradiation at 405 nm contains methoxylated **PQPhen** [(**PQPhen** + OCH_3_) + H]^+^ at *m*/*z* = 312.11 and [(**PQPhen** + OCH_3_) + Na]^+^ at *m*/*z* = 334.10 (see Figure S14). Longer (10 min) irradiation yields several other products: dihydrogenated and methoxylated **PQPhen**, methylated **PQPhen** (see Figure S15), as well as dihydrogenated and dimethoxylated **PQPhen** (see Figure S16). Several possible structures are presented in Table S5 and some of them are depicted in Chart 2, all with ambiguous positions of methoxy or methyl groups. Interestingly, neither methylation nor methoxylation results in the dehydrogenation of the compounds. The dihydrogenated compound may thus withstand the attack by methoxy radicals, thereby preserving the photochemical storage of hydrogen, as reported earlier for HATN.^14^ However, the risk of photodegradation of the reaction products via dark radical chemistry cannot be excluded.

The mass spectra of **PQ2Py** in methanol, depicted in Figure S17 before photoradiation and Figures S18–S20 after photoradiation, demonstrate that the photochemical reactions are akin to those observed for **PQPhen**. Methoxylation, dimethoxylation, and dihydrogenation of the chromophore are once again observed, confirming the previous conclusion that methoxylation does not destroy the dihydrogenated structure. Alternatively, it is possible that the methoxylated chromophore can undergo hydrogenation.

**Chart 3:**
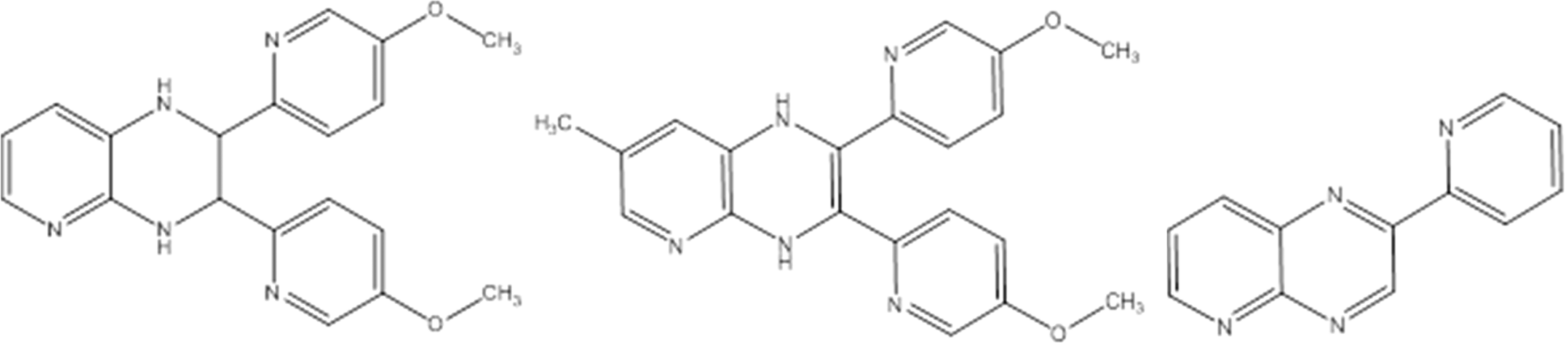
Molecular Structures of Selected Products for **PQ2Py** in Methanol

To check the stability of these reaction products, another measurement was performed, not directly after irradiation, as all spectra discussed earlier were taken, but after an additional 15 min of rest time in the dark. The effect of the chemistry of photogenerated radicals on photoproducts is depicted on Figure S21. Along with **PQ2Py** and methoxylated **PQ2Py** observed at *m*/*z* = 286.11, 308.09, and 318.13, respectively, a new structure of low mass (*m*/*z* = 209.08) was detected and assigned to **PQ2Py** missing a pyridine ring. This result indicates degradation of the chromophore and is consistent with the irreversible changes in the absorption spectra of **PQ2Py** reported above. The rest-in-the-dark test unveils the occurrence of dark chemistry involving photogenerated radicals even after the termination of photochemistry. The dark reactions will be present also during long photoirradiation and the generated radicals potentially may react several times with the photocatalyst, increasing its mass. This is observed in Figure S22, which presents the heavy masses part of the mass spectrum obtained after 30 min irradiation of **PQ2Py** with a 375 nm laser. The spectrum reveals several new heavy compounds which are not visible for shorter irradiation times. The object at *m*/*z* = 384.14 is identified as the [(**PQ2Py** + 2H + 2(OCH_3_) + CH_3_) + Na]^+^ ion and illustrates the possibility of multiple methoxylation or methylation of the chromophore. It also reveals the capacity of multiply methoxylated or methylated compounds to store two hydrogen atoms. Table S6 contains several possible photoproducts generated from **PQ2Py**. Chart 3 illustrates the two possible photochemical pathways for **PQ2Py**: the increase in mass through photocatalytic methoxylation, methylation, and hydrogenation or decomposition by loss of one of its pyridine rings.

The MS spectra taken for **Q2Py** in methanol before (Figure S23) and after (Figure S24) photoirradiation with a 277 nm LED unveil the photochemical pathways of this chromophore, which are akin to those of **PQ2Py**. For example, the methoxylated structure at *m*/*z* = 317.14 [(**Q2Py** + CH_3_O) + H]^+^ is shown in Figure S24. However, there are also differences between the reactivities of the chromophores: for **Q2Py**, several intense lines cannot be easily assigned to specific photoproducts, e.g., the line at *m*/*z* = 360.32 in Figure S24. The prediction table for this photoproduct displays a significant drop in the double bond equivalent (level of unsaturation) to 4.5 only from 14.5 obtained for bare **Q2Py (**Figure S24). This suggests ring opening and formation of alkyl groups. The spectra of **Q2PY** in the mass range less than *m*/*z* = 285.12 indicate numerous photoproducts resulting from the photodegradation of this compound (Figures S25 and S26). This confirms that decomposition of the chromophore occurs fast and is the dominant pathway. This can be related to the theoretically predicted barrierless PCET and thus the very effective hydrogenation of this chromophore and the concurrent generation of methoxy radicals. Another reason of the fast photodegradation may be related to differences of the PCET process in **PQPhen**, **PQ2Py**, and **Q2Py**. In **PQPhen**, the hydrogen bond with methanol is formed with a nitrogen atom of the pyrazine ring as depicted in Table S3. In the ^1^nπ* excited state, a hydrogen atom is transferred along this hydrogen bond to nitrogen, as illustrated in Table 3 via the NTOs of the CT state. In **Q2Py**, a hydrogen bond is also formed with a nitrogen of pyrazine, but the H-atom transfer occurs to the nitrogen of the pyridine ring, as shown in Figure 4. Consequently, the methoxy radical is located in proximity to the quinoxaline–pyridine bond and can potentially attach to either carbon atom of this bond. This may open a pathway to the detachment of the pyridine ring or ring opening.

## Conclusions

Three quinoxaline derivatives have been studied experimentally and theoretically. All compounds exhibit the lowest excited singlet state of nπ* character and a slightly higher absorbing ππ* state. The two nonplanar compounds show very weak luminescence from the lowest ^1^nπ* state, whereas a chromophore with a planar and rigid structure exhibits a moderately intense and structured non-Kasha fluorescence from the absorbing state in nonpolar and aprotic solvents. In hydrogen-bonded complexes with protic solvent molecules, the symmetry is reduced, and vibronically induced mixing of the nπ* and ππ* states occurs. This results in fast radiationless relaxation to the lowest excited singlet state and ordinary Kasha-type emission in the hydrogen-bonded complexes. The phenomenon is an unusual example of switching of the emissivity of excited states induced by intermolecular H bonding.

The present study demonstrates that direct photoinduced homolytic hydrogen abstraction from methanol is feasible for all quinoxaline derivatives forming hydrogen-bonded complexes with the solvent. In the complexes with methanol, the PCET mechanism operates effectively in the ^1^nπ* state of CT character for all three chromophores studied. This process results in the formation of hydrogenated chromophore radicals, which then yield stable dihydrogenated compounds by disproportionation. The dihydrogenated chromophores offer a potential avenue for photochemical hydrogen storage. The experimental and theoretical results shed light on a potential mechanism of photochemical hydrogen storage, and this is the main finding of the present work.

On the other hand, the observation of methoxylated, methylated, or decomposed reaction products heralds the existence of competing undesired radical chemistry leading to photodegradation of the chromophores. PCET has been widely promoted as an essential ingredient for the conversion of light to chemical energy^35^ and our findings support this concept. However, one should acknowledge that photoinduced PCET reactivity also initiates undesired side processes that must be taken into consideration. Our results show that the branched molecular structures of **PQ2Py** and **Q2Py** are more vulnerable for assail of radicals and experience nonreversible photodegradation than the concrete structure of **PQPhen**. In practical applications, the use of efficient scavengers of the oxidized radicals or the use of suitable flow reactors that separate the photoproduction of radicals from ensuing radical recombination reactions may mitigate the effects of the undesired competing reactions.

## Supporting Information Available

Synthesis of the three quinoxaline derivatives; experimental and theoretical methods; computational results; optical, NMR, and MS spectra; molecular orbitals; and potential energy surfaces (PDF)

## Abbreviations

PCET: proton-coupled electron transfer
MS: mass spectroscopy
NMR: nuclear magnetic resonance
