# Supplementary material for: Harnessing Proton-Coupled Electron Transfer for Hydrogenation of Aza-Arenes: Photochemistry of Quinoxaline Derivatives in Methanol
Source: J Phys Chem A. 2023 Oct 16;127(42):8871–81. doi: 10.1021/acs.jpca.3c05077 (PMC10614181; doi:10.1021/acs.jpca.3c05077)
Supplement: Supplementary file 1 — jp3c05077_si_001.pdf [file jp3c05077_si_001.pdf]

Supplementary Information

Harnessing Proton Coupled Electron Transfer for Hydrogenation of Aza-Arenes: Photochemistry of Quinoxaline Derivatives in Methanol

Olaf W. Morawski*, Paweł Gawryś, and Andrzej L. Sobolewski

Institute of Physics, Polish Academy of Sciences, Al. Lotników 32/46, 02-668 Warsaw, Poland

Table of content

1. Synthesis 2

2. Experimental methods 5

3. Theoretical methods 7

4. Computational results 8

5. Experimental results 16

5.1 Optical spectroscopy results 16

5.2 Electrochemical characterization 23

5.3 The ^{1}H NMR results 25

5.4 Mass Spectroscopy results 28

1. Synthesis

Synthesis of dibenzo[f, h]pyrido[2, 3-b]quinoxaline [PQ2Phen]

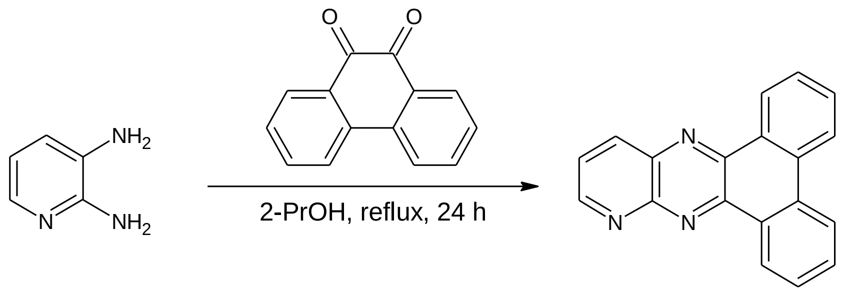

Molecular Formula	= $C_{19}H_{11}N_3$
Formula Weight	= 281.31074
Composition	= C(81.12%) H(3.94%) N(14.94%)
Monoisotopic Mass	= 281.095297 Da
M+	= 281.094749 Da
[M+H]+	= 282.102574 Da

The reaction and purification was carried out without protection from oxygen and water from air. In a one necked-flask (250 ml) was put 9,10-phenanthrenedione (2.08 g, 10 mmol, 1 equivalent); 2,3-diaminopyridyne (1.08 g; 10 mmol, 1 equivalent) and 2-propanol (any grade, 80 ml). The resulting brown-orange suspension was vigorously refluxed with stirring under condenser for 24 hours. The reaction mixture was heterogeneous entire time. The monitoring of the reaction was done by TLC analysis using dichloromethane as eluent. After the condensation was practically completed, the flask was cooled below 50 ^{o}C. The crystalline powder was filtered off on a sinter funnel (G3, 1.5 inch wide) and washed with methanol (3 x 40 ml) and dried in air for 24 hours to yield brown-yellow crystalline powder (1.91 g). By cooling of the mother liquor (freezer), second crop was obtained and isolated similarly (0.64 g). The final very impure crop could be isolated from mother liquors (0.13 g). Total yield (95%). The first crop was crystallised from boiling 1-propanol (100 ml, p.a. grade, 2-propanol is too weak solvent to crystallise the compound) and putting in the fridge overnight. The light brown needles were filtered on a sinter funnel (G3, 1.5 inch wide) and washed with 2-propanol (2 x 30 ml) and dried in air for 24 hours. (1.79 g, 63 %)

The product at this point had good NMR characteristics but it was unsuitable for the photophysical studies. 220 mg of needles were sublimed under vacuum (220°C, 10^{-3} Torr, 4 hours) in a sublimation apparatus and yellow crystalline solid was obtained (200 mg). Everything was suspended in 2-propanol (40 ml, Uvasol) and ethyl acetate was added to dissolve the product (10 ml, Uvasol). The solution was boiled until the volume was reduced to 30 ml. The resulting pale yellow solution was put in the freezer (-25°C) for 48 hours. The crystallisation yielded pale yellow needles which were filtered on a sinter funnel (G3, 1 inch wide) and washed with 2-propanol (2 x 30 ml, Uvasol). The collected crystals were dried in air for 24 hours and in vacuum desiccator (6h, 75°C, 2 mbar). Pale yellow needles (178 mg). The compound is known (I. Pyszka et *al*., Materials 2021, 14, 3085, https://doi.org/10.3390/ma14113085)

<u>HRMS (ESI^{+}):</u> calculated M+H^{+}: 282.1026, found: 282.1037, error: 3.90 ppm.

<u>^{1}H NMR (CD_3OD, 298 K):</u> 9.45 (m, 1H), 9.38 (m, 1H), 9.29 (m, 1H), 8.84 (m, 1H), 8.75 (m, 1H + 1H), 8.00 (m, 1H), 7.91 (m, 1H + 1H), 7.82 (m, 1H + 1H).

<u>^{1}H NMR ($CDCl_3$+TMS, 298 K):</u> 9.53 (m, 1H), 9.32 (m, 1H), 9.30 (m, 1H), 8.66 (m, 1H), 8.54 (m, 1H+1H), 7.78-7.85 (m, 1H + 1H + 1H), 7.71-7.78 (m, 1H + 1H).

<u>^{13}C {^{1}H} NMR / ^{13}C {^{1}H} DEPT135 NMR ($CHCl_3$ + TMS, 298 K):</u> 154.45 (C-H), 149.77 (C), 144.86 (C), 143.46 (C), 138.24 (C-H), 137.24 (C), 132.38 (C), 132.16 (C), 131.06 (C-H), 130.75 (C-H), 129.64 (C), 129.41 (C), 128.05 (C-H), 127.92 (C-H), 127.25 (C-H), 126.37 (C-H), 124.79 (C-H), 122.88 (C-H), 122.76 (C-H).

<u>Melting point (2-propanol):</u> 222-223°C.

Synthesis of 2,3-di(2-pyridyl)pyrido[2,3-*b*]pyrazine [PQ2Py]

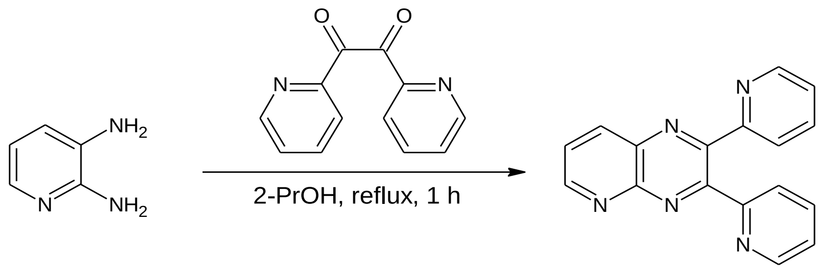

Molecular Formula = $C_{17}H_{11}N_5$
Formula Weight = 285.30274
Composition = C(71.57%) H(3.89%) N(24.55%)
Monoisotopic Mass = 285.101445 Da
M+ = 285.100897 Da
[M+H]+ = 286.108722 Da

The reaction and purification was carried out without protection from oxygen and water from air. In a one necked-flask (250 ml) was put 1,2-di(2-pyridyl)ethanedione (2,2'-Pyridil, 2.12 g, 10 mmol, 1 equivalent); 2,3-diaminopyridyne (1.08 g; 10 mmol, 1 equivalent) and 2-propanol (any grade, 80 ml). The resulting suspension was vigorously refluxed with stirring under condenser for 1 hour. The initial suspension promptly formed black solution. The monitoring of the reaction was done by TLC analysis using ethyl acetate as eluent. After the condensation was practically completed, the flask was cooled below 50 ^{o}C and filtered through a pad of cotton wool to remove insolubles. Next, the solution was put in the freezer (-25 ^{o}C) and gradually diluted with hexanes (3 x 50 ml) (the product crystallised capriciously at this step) to induce separation of the solid phase. After several days the beige solid was filtered off on a sinter funnel (G3, 1.5 inch wide) and washed with hexanes (3 x 30 ml) and dried in air for 24 hours. Beige powder (2.07 g, 72%). The mother liquor was kept. The obtained product was crystallised from boiling 2-propanol (20 ml, p.a. grade), quick diluting with *n*-heptane (3 x 20 ml) and leaving at ambient temperature. This time the crystallisation was quick and after 24 hours the light beige crystals were filtered on a sinter funnel (G3, 1.5 inch wide) and washed with hexanes (3 x 30 ml) and dried in air for 24 hours. (1.74 g, 61 %).

The product at this point had good NMR characteristics but it was unsuitable for the photophysical studies. 250 mg of crystals were sublimed under vacuum (180°C, 10^{-3} Torr, 4 hours) in a sublimation apparatus and white crystalline solid was obtained (235 mg). Everything was dissolved in boiling 2-propanol (15 ml, Uvasol) and *n*-heptane was quickly added (2 x 25 ml, Uvasol) and the flask put in the freezer (-25°C). Nothing happened and the crystallisation could only be induced by shock on the next day and after further 24 hours the colourless crystals were filtered off on a sinter funnel (G3, 1 inch wide) and washed with *n*-heptane (3 x 20 ml, Uvasol). The collected crystals were dried in air for 24 hours and in vacuum desiccator (6h, 75°C, 2 mbar). Very faint yellow crystals (130 mg). The compound is known (J. J. Morales-Castellanos et *al*., Molecules 2012, 17, 5164-5176; https://doi.org/10.3390/molecules17055164)

HRMS (ESI^{+}): calculated M+H^{+}: 286.1087, found: 286.1097, error: 3.50 ppm.

^{1}H NMR (CD_3OD, 298 K): 9.23 (m, 1H), 8.71 (m, 1H), 8.29 (m, 2H), 8.16 (m, 1H), 7.95-8.05 (m, 4H), 7.41 (m, 2H).

^{1}H NMR ($CDCl_3$+TMS, 298 K): 9.22 (m, 1H), 8.58 (m, 1H), 8.39 (m, 1H), 8.33 (m, 1H), 8.27 (m, 1H), 7.98 (m, 1H), 7.89 (m, 1H), 7.85 (m, 1H), 7.78 (m, 1H), 7.23-7.29 (m, 1H + 1H).

^{13}C {^{1}H} NMR / ^{13}C {^{1}H} DEPT135 NMR ($CHCl_3$ + TMS, 298 K): 157.07 (C), 156.47 (C), 155.18 (C), 154.55 (C-H), 153.91 (C), 149.64 (C), 148.57 (C-H), 148.09 (C-H), 138.23 (C-H), 136.83 (C-H), 136.65 (C-H), 136.22 (C), 125.62 (C-H), 124.66 (C-H), 124.05 (C-H), 123.44 (C-H), 123.14 (C-H).

Melting point (2-propanol : *n*-heptane 1:4): 148-150°C.

Synthesis of 2,3-di(2-pyridyl)quinoxaline [Q2Py]

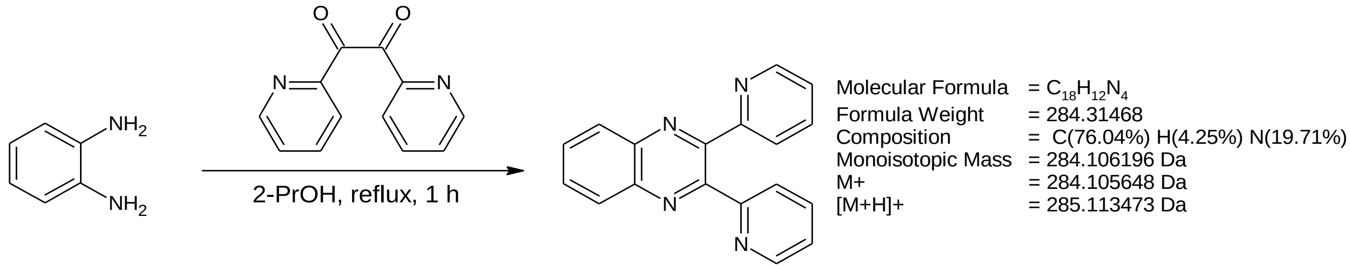

The reaction and purification was carried out without protection from oxygen and water from air. In a one necked-flask (250 ml) was put 1,2-di(2-pyridyl)ethanedione (2,2'-Pyridil, 2.12 g, 10 mmol, 1 equivalent); 1,2-phenylenediamine (1.08 g; 10 mmol, 1 equivalent) and 2-propanol (any grade, 80 ml). The resulting suspension was vigorously refluxed with stirring under condenser for 1 hour. The initial suspension slowly formed brown solution. The monitoring of the reaction was done by TLC analysis using ethyl acetate as eluent. After the condensation was practically completed, the flask was cooled below 50 ^{o}C and put in the freezer (-25 ^{o}C) for 24 hours. On the next day the obtained crystals were filtered off on a sinter funnel (G3, 1.5 inch wide) and washed with hexanes (3 x 30 ml) and dried in air for 24 hours to yield white crystals with brown tan. (2.54 g, 89%). The mother liquor was rejected.

The product at this point had good NMR characteristics but it was unsuitable for the photophysical studies. 265 mg of crystals were sublimed under vacuum (180°C, 10^{-3} Torr, 4 hours) in a sublimation apparatus and white-yellowish crystalline solid was obtained (215 mg). Everything was dissolved in boiling 2-propanol (15 ml, Uvasol) and *n*-heptane was quickly added (25 ml, Uvasol) and the flask put in the freezer (-25°C). After 24 hours the nearly colourless crystals were filtered off on a sinter funnel (G3, 1 inch wide) and washed with *n*-heptane (3 x 20 ml, Uvasol). The collected crystals were dried in air for 24 hours and in vacuum desiccator (6h, 75°C, 2 mbar). White crystals (200 mg). The compound is known (J. J. Morales-Castellanos et *al*., Molecules 2012, 17, 5164-5176; https://doi.org/10.3390/molecules17055164)

<u>HRMS (ESI^{+}):</u> calculated M+H^{+}: 285.1135, found: 285.1140, error: 1.75 ppm.

<u>^{1}H NMR (CD_3OD, 298 K):</u> 8.30 (m, 2H), 8.24 (m, 2H), 7.92-8.02 (complicated m, 2H + 2H + 2H), 7.39 (m, 2H).

<u>^{1}H NMR (D_2O, 298 K):</u> 8.41 (m, 2H), 8.29 (m, 2H), 8.07 (m, 2H), 8.01 (m, 2H), 7.82 (m, 2H), 7.52 (m, 2H).

<u>^{1}H NMR ($CDCl_3$+TMS, 298 K):</u> 8.39 (m, 2H), 8.23 (m, 2H), 7.96 (m, 2H), 7.80-7.85 (complicated m, 2H + 2H), 7.24 (m, 2H).

<u>^{13}C {^{1}H} NMR / ^{13}C {^{1}H} DEPT135 NMR ($CHCl_3$ + TMS, 298 K):</u> 157.41 (C), 152.46 (C), 148.60 (C-H), 141.14 (C), 136.62 (C-H), 130.47 (C-H), 129.38 (C-H), 124.22 (C-H), 122.95 (C-H).

<u>Melting point (2-propanol : *n*-heptane 1:4):</u> 179.5-180 ^{o}C.

2. Experimental methods

Spectroscopic grade solvents from Sigma-Aldrich were used as received.

Optical measurements were performed at room temperature with dilute solutions in standard quartz cells (10 × 10 mm). Solutions were N2 purged and stoppers were wrapped with laboratory film to slow down the oxygen penetration. Absorption spectra at room temperature (21°C) were recorded using a PerkinElmer Lambda 35 spectrophotometer. Emission spectra were obtained using a FLS 1000 of Edinburgh Instruments spectrofluorometer. Fluorescence kinetics studies were performed using the time correlated single photon counting technique. A mode-locked Coherent Mira-HP picosecond laser pumped by a Verdi 18 laser was used for excitation. The fundamental pulses of the Mira laser (tunable within 760 - 800 nm) were up-converted to ~390 nm. The temporal width of the excitation pulses was ~280 fs and the instrument response function (IRF) about 40 ps. For shorter wavelength excitation Delta diode laser 336 nm was used. Fluorescence was dispersed with a 0.25 m Jarrell-Ash monochromator and detected with a HMP-100-07 hybrid detector coupled to an SPC-150 PC module, (Becker&Hickl GmbH). Fluorescence decays were analyzed with deconvolution software using a nonlinear least squares procedure with the Marquardt algorithm.[1] A standard $\chi2$ test and Durbin-Watson (DW) parameter along with residual and autocorrelation function plots were used to assess the quality of a fit. The estimated accuracy for the determination of decay time was below 10 ps.

For EPR spin measurements the samples were placed in suprasil EPR tubes. A EMXplus EPR Bruker CW X-band spectrometer equipped with an ER 4131VT nitrogen cryostat was used to measure spectra at 100 K. The samples were degassed with freeze – pump – thaw method and sealed to block the molecular oxygen.

1H FT-NMR spectra were recorded using Bruker AVANCE 500 MHz spectrometer. The chemical shifts are given with the residual solvent peak of methanol-d_4. The sample was a saturated solution of HATN which was further transferred to NMR tube and efficiently purged with argon.

Mass spectrometry analyses were performed using Ultra-Performance Liquid Chromatograph ACQUITY UPLC I-Class (Waters) coupled with Synapt G2-S mass spectrometer (Waters) equipped with the electrospray ion source and quadrupole-Time-of-flight mass analyzer. The resolving power of the TOF analyzer was set to 20000 FWHM. The instrument was controlled and recorded data were processed using the MassLynx V4.1 software package (Waters). Both the positive and the negative ion modes were used in mass spectrometry. The measurements in positive mode were performed with capillary voltage set to 3.00 kV. The desolvation gas flow was 700 L/h and temperature 300 °C. The sampling cone voltage and source offset were set to 20 V and the source temperature was 120 °C. The measurements in negative ion mode were performed with capillary voltage set to 3.00 kV. The desolvation gas flow was 700 L/h and temperature 300 °C. The sampling cone voltage and source offset were set to 20 V, and the source temperature was 120 °C. Samples were dissolved in methanol, degassed and injected directly into the electrospray ion source. Methanol was used as a mobile phase with the flow rate 100 μl/min. The instrument worked with external calibration of sodium formate in the mass range of m/z = 50-1200. The Leucine-Enkephaline solution was used as the Lock-Spray reference material. The lock spray spectrum of the leucine-enkephalin was generated by the lock spray source and correction was done for every spectrum. The exact mass measurements for all peaks were performed within 3 mDa mass error.

Cyclic voltammetry measurements were performed using an SP-50 Potentiostat (BioLogic). All operations involving the electrolyte preparation and electrochemical measurements were carried out under dry argon atmosphere. Cyclic voltammetry studies were carried out for the 5x10-4 M solutions in 0.1 M $[NBu_4][PF_6]$ (tetra(*n*-butyl)ammonium hexafluorophosphate) in appropriate solvent (dichloromethane, acetonitrile, methanol or their mixtures). The choice of the solvent was dictated by the solubility of the studied compound. The electrolyte was placed in a one-compartment, three electrode electrochemical cell with a

glassy carbon disk working electrode of the surface area of 3 mm2, a platinum wire counter electrode and an silver wire pseudo-reference electrode reference electrode. To make the results comparable (using of the different solvents and pseudo-reference electrode) the potential of the Fc/Fc+ redox couple (internal reference)was checked at the end of each experiment and the obtained voltammograms were shifted to set zero at potential for the Fc/Fc^{+} redox couple.

<u>The rough evaluation of the polar solute – polar solvent interaction influence on the barrier of the PCET reaction</u> can be straightforwardly carried out within the continuous dielectric medium and point dipole approximation. The polar molecule with dipole moment μ and radius *a* induces in the continuous dielectric medium of dielectric constant ε the electric reaction field *R* which is proportional to the dipole moment μ and solvent polarizability *f* (the polarizability of the solute is neglected) [2]:

$$R = \frac{2\mu}{4\pi\varepsilon_0 hca^3} \cdot f \qquad (1)$$

The *f* is a result of solvent's electronic polarizability $f(n) = (n^2-1)/(2n^2+1)$ and dielectric polarizability $f(\varepsilon) = (\varepsilon-1)/(2\varepsilon+1)$. The reaction field is a sum of terms corresponding to the electronic and orientation polarizations [2]:

$$R_{el} = \frac{\mu}{2\pi\varepsilon_0 hca^3} f(n) \qquad R_{or} = \frac{\mu}{2\pi\varepsilon_0 hca^3} \Delta f(\varepsilon, n) \qquad (2)$$

where $\Delta f(\varepsilon,n) = f(\varepsilon) - f(n)$. The energy of the dipole moment μ in reaction field R is:

$$E_d = -\mu \cdot R = -\mu(R_{el} + R_{or}) = -\mu \frac{\mu}{2\pi\varepsilon_0 hca^3} \cdot f(\varepsilon) \qquad (3)$$

In a solvent the excited state energy $E(\mu)$ is the calculated (for gas phase) value E_v decreased by the E_d :

$$E(\mu) = E_v - E_d = E_v - \frac{\mu^2}{2\pi\varepsilon_0 hca^3} \cdot f(\varepsilon) \qquad (4)$$

With this formula one may calculate the energy of the excited state in a polar solvent of a given dielectric constant at two OH distances and check how much the energy barrier between them changes. The radius of Onsager cavity for **PQPhen** was assumed a = 0.6 nm, dielectric constant of methanol ε=32.66 and the dipole moment values for S_1 μ(d(OH) = 0.963 Å) = 3.10 D, μ(d(OH) = 1.04 Å) = 3.19 D and for S_2 μ(d(OH) = 0.97 Å) = 12.79 D, μ(d(OH) = 1.12 Å) = 13.21 D at point of the energy minimum and at the charge transfer point in each state respectively were taken from calculations for the gas phase. With formula (3) one gets for the assumed parameters a bathochromic shift 0.0265 and 0.0281 eV for the S_1 state and 0.451 and 0.481 eV the S_2 state at minimum energy and PCET occurrence points respectively. The difference between them, 1.6 and 30 meV, gives the decrease of the barriers in the S_1 and S_2 respectively. This result is obtained for a non-polarizable molecular dipole. It is known that usually the reaction field increases the dipole moment of the polarizable point dipole by 20 – 50 % [3]. However, even taking into account such correction, gas phase barriers are still substantially higher than the calculated change of the solvatochromic shift.

References

[1] J. N. Demas, *Excited State Lifetime Measurements*, Academic Press: New York, **1983**.
[2] J. R. Lakowicz, *Principles of Fluorescence Spectroscopy*, Springer Science+Business Media, LLC, New York, NY, USA, **2009**, pp. 210-212.
[3] C.J.F. Böttcher, *Theory of Electric Polarization*, Elsevier Scientific Publishing Company, Amsterdam, **1973**, pp.134-137

3. Theoretical methods

The ground-state equilibrium geometries of the compounds considered herein were determined with the second-order Møller-Plesset (MP2) method.[1] Vertical electronic excitation energies of singlet and triplet excited states were calculated with the algebraic-diagrammatic construction of second order (ADC(2)) method.[2] ADC(2) is a computationally efficient single-reference propagator method which generally yields similar results as the CC2 method[3], and more accurate methods, such as CASPT2 or CC3.[4,5] The correlation-consistent polarized valence-split double-ζ basis set (cc-pVDZ)[6] was employed in all calculations. The calculations were performed with the TURBOMOLE program package (V7.4)[7] making use of the resolution-of-the-identity (RI) approximation.[8]

References

[1] C. Møller and M. S. Plesset, *Phys. Rev.*, 1934, **46**, 618-622.

[2] J. Schirmer, *Phys. Rev. A*, 1982, **26**, 2395-2416.

[3] O. Christiansen, H. Koch and P. Jorgensen, *Chem. Phys. Lett.*, 1995, **243**, 409-418.

[4] S. P. A. Sauer, M. Schreiber, M. R. Silva-Junior and W. Thiel, *J. Chem. Theory Comput.*, 2009, **5**, 555-564.

[5] P.-F. Loos, F. Lipparini, M. Boggio-Pasqua, A. Scemama and D. Jacquemin, *J. Chem. Theory Comput.*, 2020, **16**, 1711-1741.

[6] T. H. Dunning Jr., *J. Chem. Phys.*, 1989, **90**, 1007-1023.

[7] TURBOMOLE V7.4 2019, a Development of Univeristy of Karlsruhe and Forschungszentrum Karlsruhe GmbH, 1989-2007, TURBOMOLE GmbH, since 2007; Available from Http://Www.Turbomole.Com.

[8] C. Hättig and F. Weigend, *J. Chem. Phys.*, 2000, **113**, 5154-5161.

4. Computational results

Table S1. Vertical absorption energy (ΔE), oscillator strength (f), dipole moment (μ), leading electronic configurations and relevant orbitals of the lowest excited states of **PQPhen**, **PQ2Py** and **Q2Py** determined with the ADC(2)/cc-pVDZ method at the MP2/cc-pVDZ equilibrium geometry of the ground state.

PQPhen

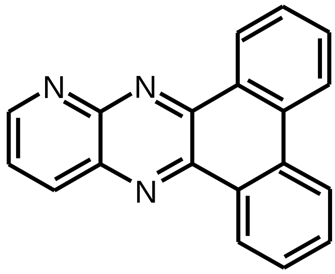

State	ΔE/eV	f	μ/Debye	Electronic Configuration
S0	0.0	-	2.14	(62a')2(11a")2
$^{3}n\pi^*$	2.932	-	1.09	0.84(62a'-12a")
$^{3}\pi\pi^*$	3.011	-	3.19	0.62(10a"-12a")+0.52(9a"-12a")
$^{1}n\pi^*$	3.226	0.0	1.20	0.97(62a'-12a")
$^{1}\pi\pi^*$	3.641	0.341	4.19	0.87(11a"-12a")
$^{1}\pi\pi^*$	3.754	0.031	12.23	0.92(10a"-12a")
$^{1}\pi\pi^*$	4.367	0.004	5.50	0.90(9a"-12a")
$^{1}\pi\pi^*$	4.440	0.184	3.16	0.60(10a"-13a")-0.53(11a"-14a")

LUMO		
12a"	13a"	14a"
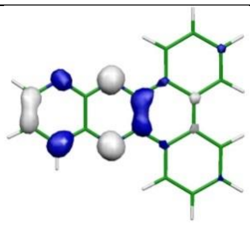	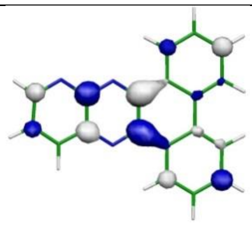	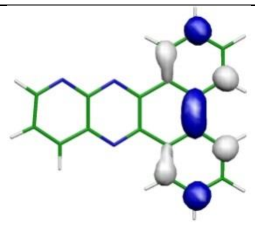
**HOMO**		
62a'	10a"	11a"
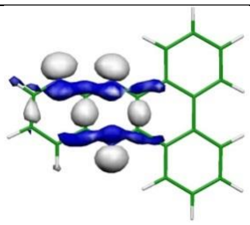	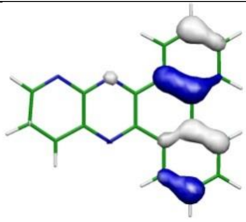	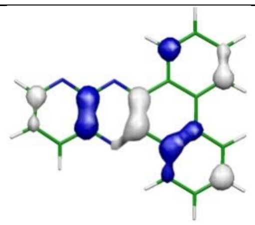

PQ2Py

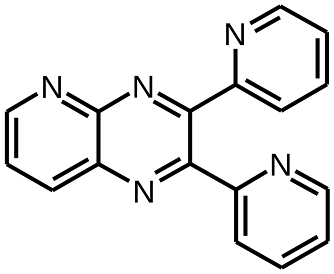

State	ΔE/eV	f	μ/Debye	Electronic Configuration
S_0	0.0	-	2.37	$(74a)^2$
$^3n\pi^*/^3\pi\pi^*$	3.127	-	1.20	0.78(68a-75a)-0.40(69a-75a)
$^3\pi\pi^*$	3.498	-	1.81	0.61(73a-75a)-0.44(74a-76a)+0.43(71a-75a)
$^3\pi\pi^*$	3.565	-	4.11	0.89(74a-75a)
$^1n\pi^*/^1\pi\pi^*$	3.466	0.001	0.81	0.85(68a-75a)-0.41(69a-75a)
$^1\pi\pi^*$	4.030	0.308	3.61	0.87(74a-75a)
$^1n\pi^*/^1\pi\pi^*$	4.290	0.011	1.25	0.77(68a-76a)-0.42(69a-76a)
$^1n\pi^*$	4.515	0.008	3.37	0.54(66a-75a)+0.49(65a-75a)
$^1n\pi^*$	4.643	0.002	8.53	0.52(67a-75a)-0.44(66a-76a)
$^1n\pi^*$	4.739	0.018	3.40	0.59(65a-75a)+0.46(67a-76a)
$^1\pi\pi^*$	4.760	0.085	3.96	0.64(73a-75a)-0.57(74a-76a)
$^1\pi\pi^*$	4.957	0.218	3.77	0.53(73a-76a)+0.43(72a-75a)

LUMO			
75a		76a	
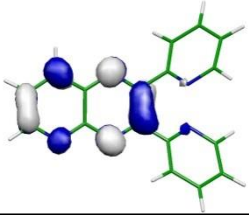		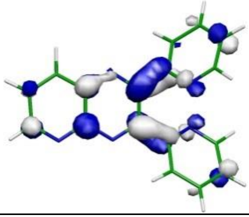	
**HOMO**			
69a	71a	73a	74a
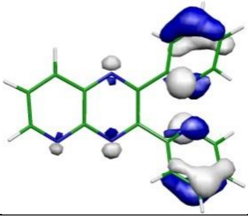	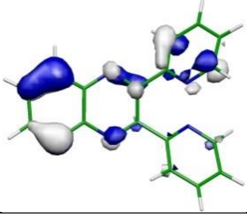	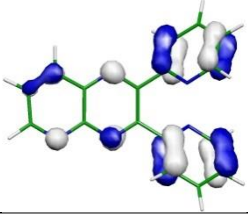	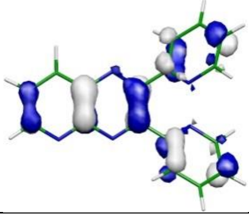
65a	66a	67a	68a
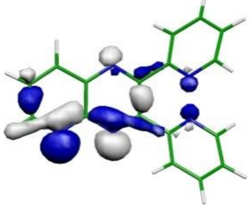	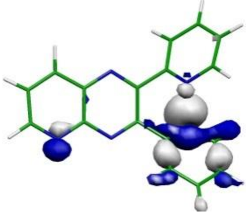	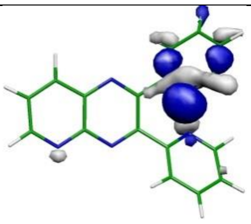	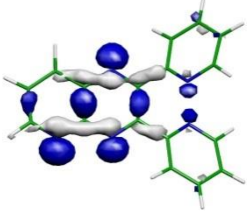

Q2Py

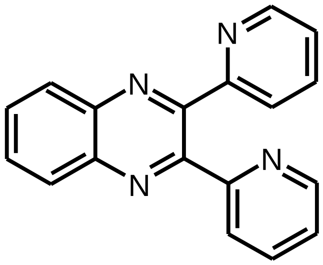

State	ΔE/eV	f	μ/Debye	Electronic Configuration
S_0	0.0	-	0.97	$(38a)^2(36b)^2$
$^3\pi\pi^*$	3.618	-	2.20	0.87(36b-37b)
$^3\pi\pi^*$	3.175	-	1.13	0.79(38a-37b)
$^3n\pi^*$	3.459	-	1.22	0.72(35a-37b)
$^1n\pi^*$	3.819	0.004	2.18	0.81(35a-37b)
$^1\pi\pi^*$	4.021	0.168	0.64	0.82(36b-37b)-0.40(38a-39a)
$^1\pi\pi^*$	4.474	0.002	3.38	0.91(38a-37b)
$^1n\pi^*/^1\pi\pi^*$	4.484	0.034	1.98	0.76(35a-39a)+0.40(36a-39a)
$^1n\pi^*$	4.714	0.012	4.63	0.57(33b-39a)-0.53(34a-37b)
$^1n\pi^*$	4.790	0.001	6.27	0.59(33b-37b)-0.54(34a-39a)
$^1\pi\pi^*$	4.891	0.144	1.31	0.53(36b-39a)-0.39(37a-37b)-0.34(36b-40a)

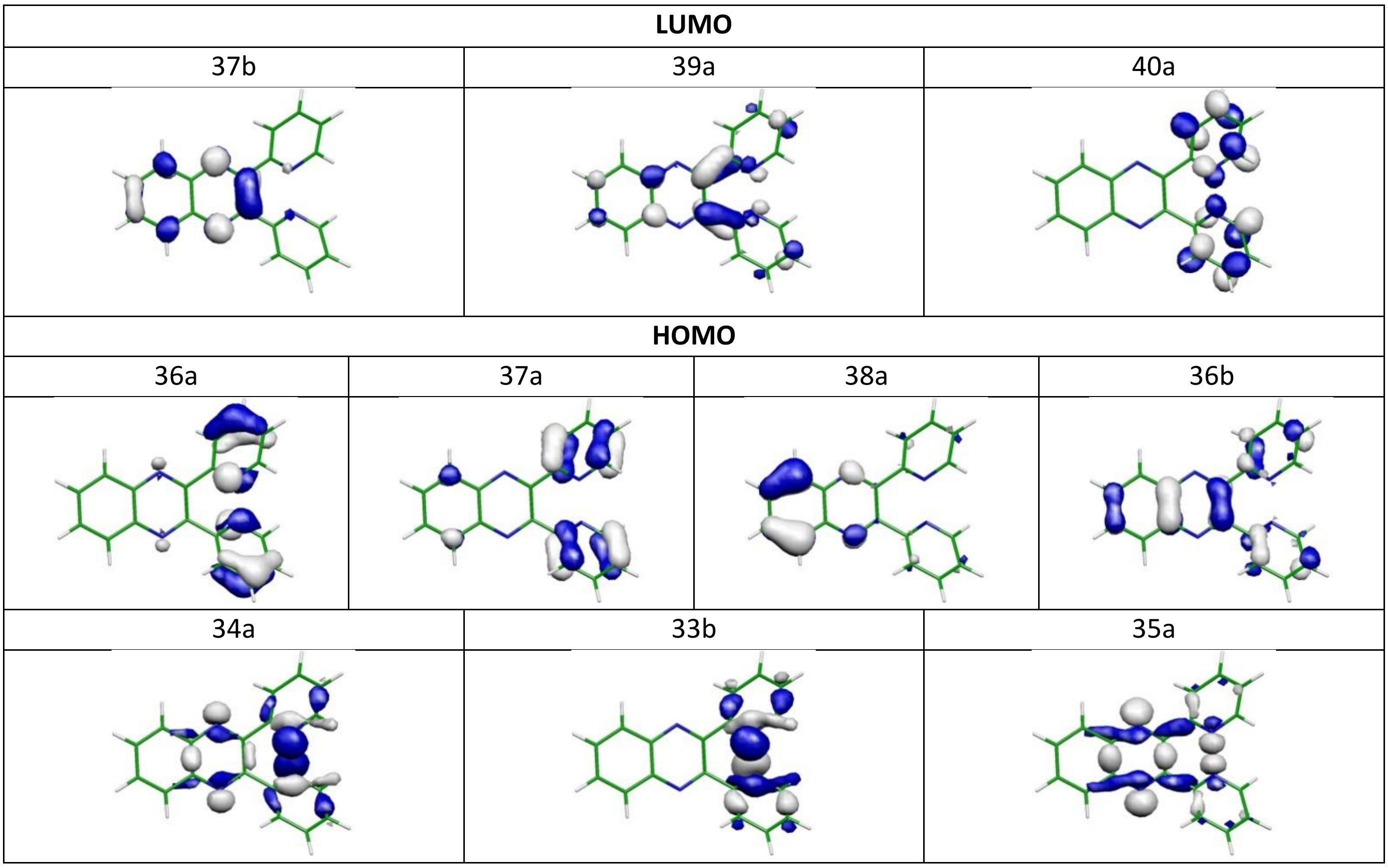

Table S2. Relative enthalpy, $E(S_0)$, adiabatic energy of the S_1 state, $E(S_1^{abo})$, the vertical energy, E_{fl}, and the oscillator strength, f, of the S_1-S_0 fluorescence of the lowest-energy conformers of hydrogen bonded complexes of **PQPhen**, **PQ2Py** and **Q2Py** with methanol molecule determined with the ADC(2)/cc-pVDZ method at the MP2/cc-pVDZ equilibrium geometry of the ground state. Energies are in electronvolts.

System	**PQPhen-1**	**PQPhen-2**
**PQPhen-MeOH**	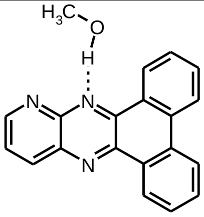	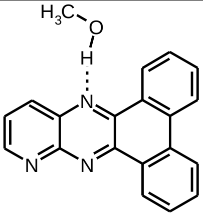
$E(S_0)$	0.0	0.019
$E(S_1^{abo})$	2.817	2.811
E_{fl}	2.252	2.265
f	$0.1x10^{-4}$	$0.1x10^{-4}$

System	**PQ2Py-1**	**PQ2Py -2**	**PQ2Py -3**	**PQ2Py -4**
**PQ2Py-MeOH**	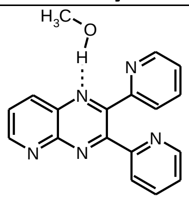	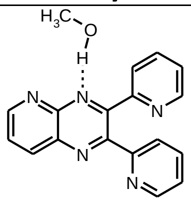	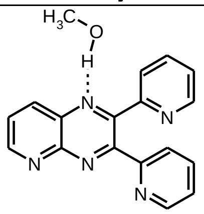	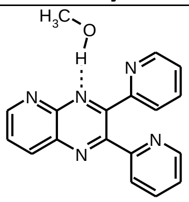
$E(S_0)$	0.0	0.0013	0.084	0.125
$E(S_1^{abo})$	2.903	2.996	2.999	3.048
E_{fl}	2.326	2.381	2.361	2.332
f	$0.3x10^{-3}$	$0.2x10^{-3}$	$0.6x10^{-4}$	$0.2x10^{-3}$

System	**Q2Py-1**	**Q2Py-2**
**Q2Py-MeOH**	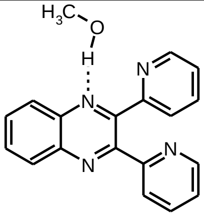	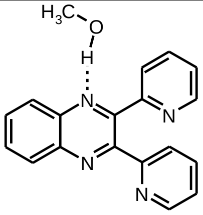
$E(S_0)$	0.0	0.038
$E(S_1^{abo})$	-	3.439
E_{fl}	-	2.952
f	-	$0.77x10^{-2}$

Table S3. Vertical absorption energy (ΔE), oscillator strength (f), dipole moment (μ), leading electronic configurations and relevant orbitals of the lowest excited states of the lowest-energy hydrogen-bonded complexes of **PQPhen**, **PQ2Py** and **Q2Py** with methanol molecule determined with the ADC(2)/cc-pVDZ method at the MP2/cc-pVDZ equilibrium geometry of the ground state.

PQPhen - MeOH

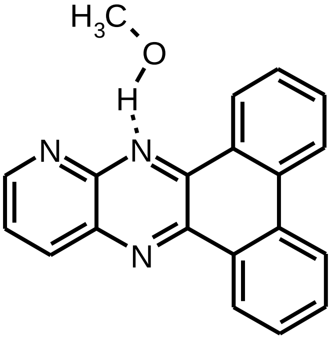

State	ΔE/eV	f	μ/Debye	Electronic Configuration
S_0	0.0	-	4.17	$(82a)^2$
$^3\pi\pi^*$	2.972	-	4.77	0.49(81a-83a)+0.49(80a-83a)-0.44(82a-83a)
$^3n\pi^*$	2.992	-	6.34	0.88(76a-83a)
$^1n\pi^*$	3.278	0.0	3.01	0.96(76a-83a)
$^1\pi\pi^*$	3.477	0.305	6.56	0.89(82a-83a)
$^1\pi\pi^*$	3.597	0.073	11.98	0.92(81a-83a)
$^1\pi\pi^*$	4.269	0.006	7.74	0.92(80a-83a)
$^1\pi\pi^*$	4.401	0.213	4.96	0.53(81a-84a)-0.44(82a-84a)

LUMO	
83a	84a
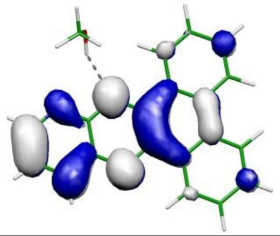	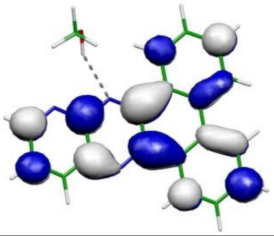

HOMO			
76a	80a	81a	82a
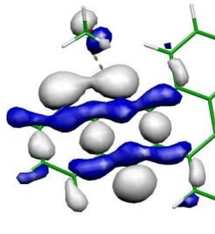	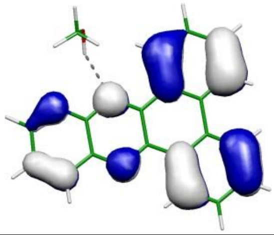	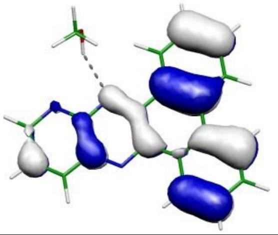	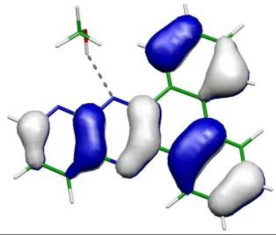

PQ2Py - MeOH

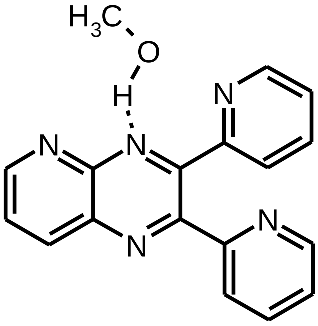

State	ΔE/eV	f	μ/Debye	Electronic Configuration
S_0	0.0	-	1.97	$(83a)^2$
$^3n\pi^*$	3.117	-	1.61	0.86(77a-84a)
$^3\pi\pi^*$	3.416	0.001	1.53	0.56(83a-84a)+0.52(82a-84a)
$^1n\pi^*$	3.443	0.001	1.39	0.91(77a-84a)
$^1\pi\pi^*$	3.977	0.308	5.30	0.88(83a-84a)
$^1n\pi^*$	4.241	0.024	6.63	0.57(74a-84a)+0.44(77a-85a)
$^1n\pi^*$	4.458	0.017	1.77	0.74(77a-85a)
$^1n\pi^*$	4.586	0.005	5.82	0.59(75a-84a)-0.52(73a-84a)
$^1\pi\pi^*$	4.728	0.056	4.99	0.55(82a-84a)-0.47(83a-84a)
$^1n\pi^*$	4.758	0.032	5.25	0.44(75a-84a)+0.41(75a-85a)
$^1\pi\pi^*$	4.922	0.133	5.15	0.52(82a-85a)

<table>
<tr><th colspan="6">LUMO</th></tr>
<tr><td colspan="3">84a</td><td colspan="3">85a</td></tr>
<tr><td colspan="3">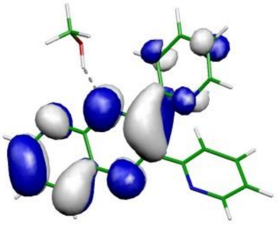</td><td colspan="3">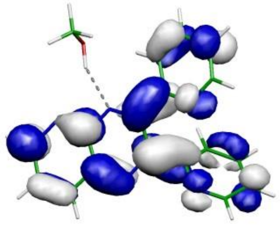</td></tr>
<tr><th colspan="6">HOMO</th></tr>
<tr><td colspan="2">77a</td><td colspan="2">82a</td><td colspan="2">83a</td></tr>
<tr><td colspan="2">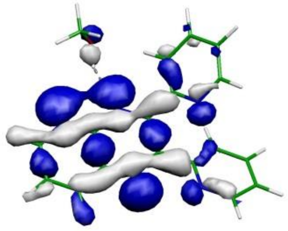</td><td colspan="2">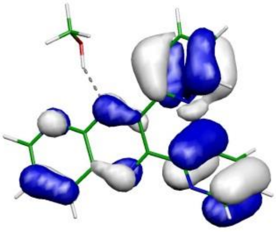</td><td colspan="2">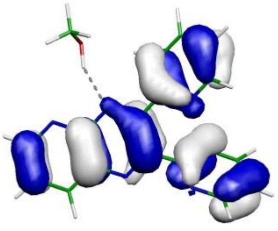</td></tr>
<tr><td colspan="2">73a</td><td colspan="2">74a</td><td colspan="2">75a</td></tr>
<tr><td colspan="2">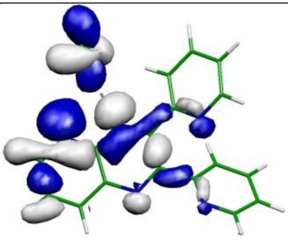</td><td colspan="2">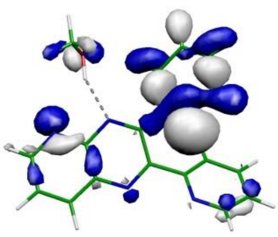</td><td colspan="2">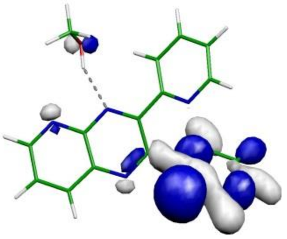</td></tr>
</table>

Q2Py - MeOH

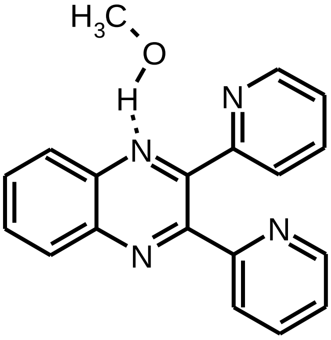

State	ΔE/eV	f	μ/Debye	Electronic Configuration
S_0	0.0	-	5.79	$(83a)^2$
$^{3}\pi\pi^*$	3.109	-	4.79	0.84(82a-84a)
$^{3}n\pi^*$	3.403	-	5.47	0.70(76a-84a)-0.41(77a-84a)
$^{1}n\pi^*$	3.779	0.004	5.63	0.74(76a-84a)-0.45(77a-84a)
$^{1}\pi\pi^*$	3.964	0.156	5.19	0.82(83a-84a)-0.41(82a-85a)
$^{1}\pi\pi^*$	4.293	0.009	6.18	0.92(82a-84a)
$^{1}n\pi^*$	4.452	0.015	5.09	0.61(76a-85a)
$^{1}n\pi^*$	4.646	0.006	7.19	0.52(75a-84a)-0.52(75a-85a)
$^{1}n\pi^*$	4.696	0.005	6.49	0.48(74a-84a)-0.41(76a-85a)+0.41(74a-85a)
$^{1}\pi\pi^*$	4.826	0.499	6.12	0.51(82a-85a)-0.36(83a85a)

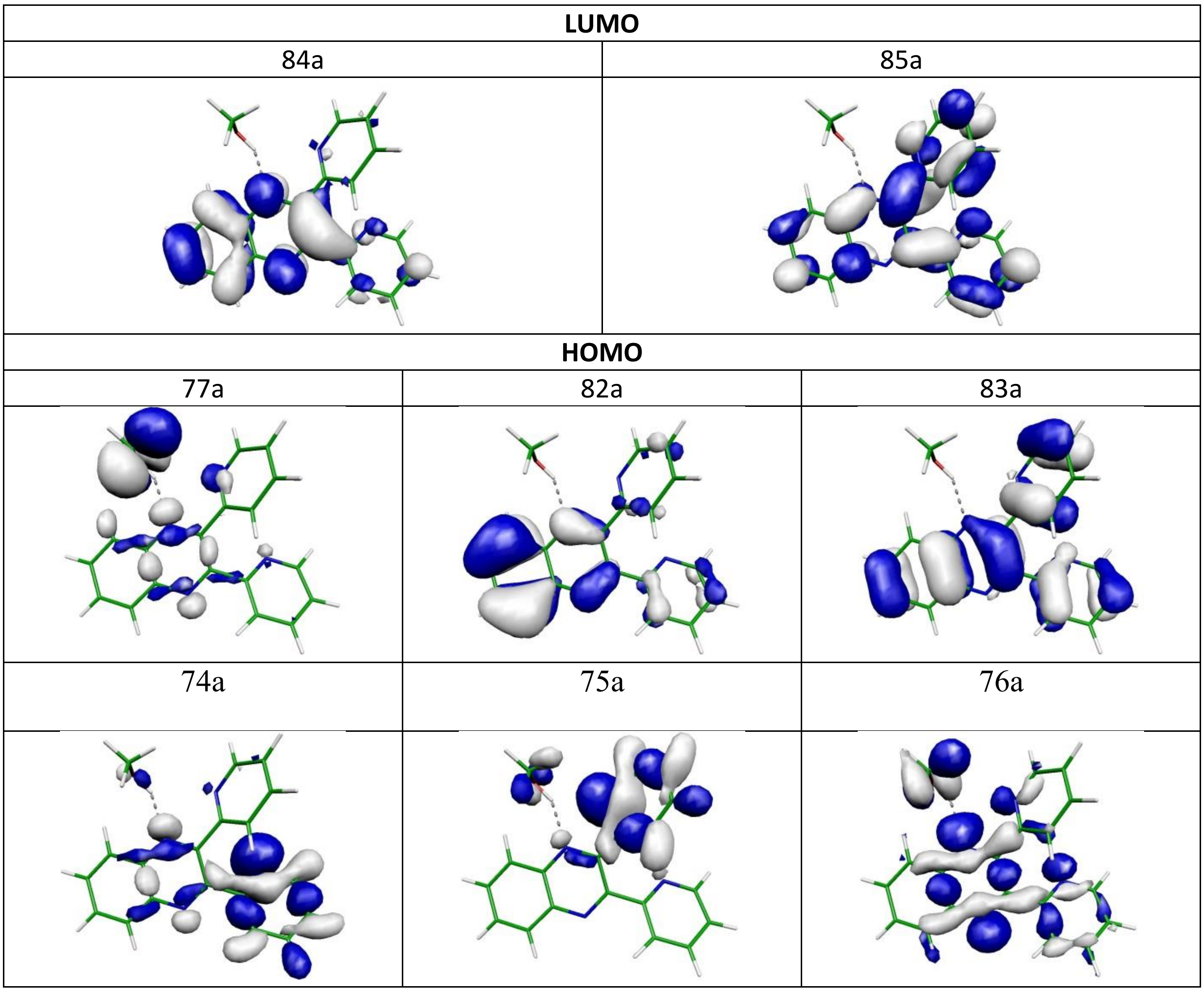

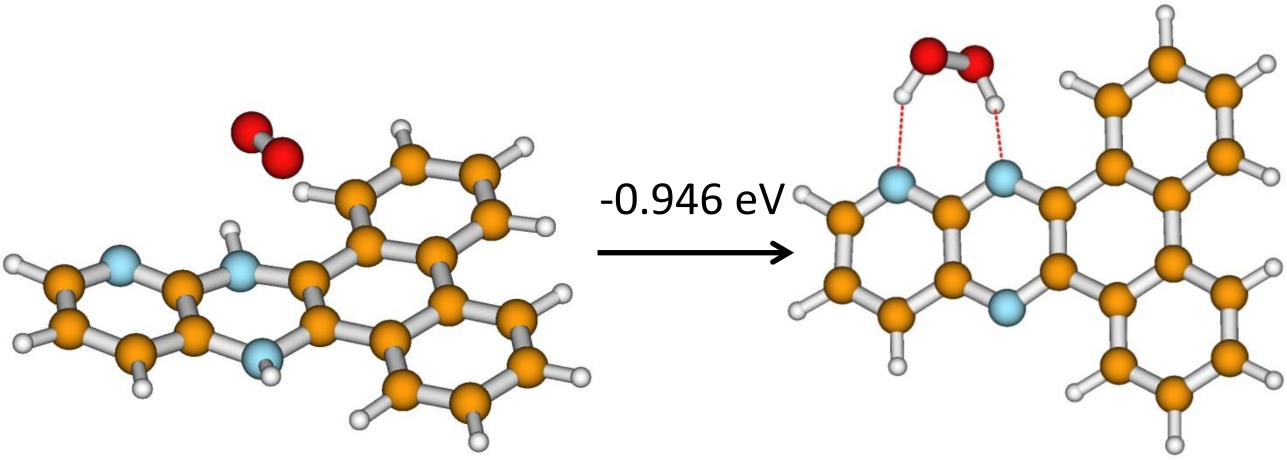

Scheme S1. Spontaneous dehydrogenation of dehydrogenated **PQPhen** by molecular oxygen. Enthalpy of the **PQPhen-H$_2$** + O_2 → **PQPhen** + H_2O_2 reaction calculated at the DFT/D3-B3LYP/cc-pVDZ level of theory is -0.946 eV.

5. Experimental results

5.1 Optical spectroscopy results

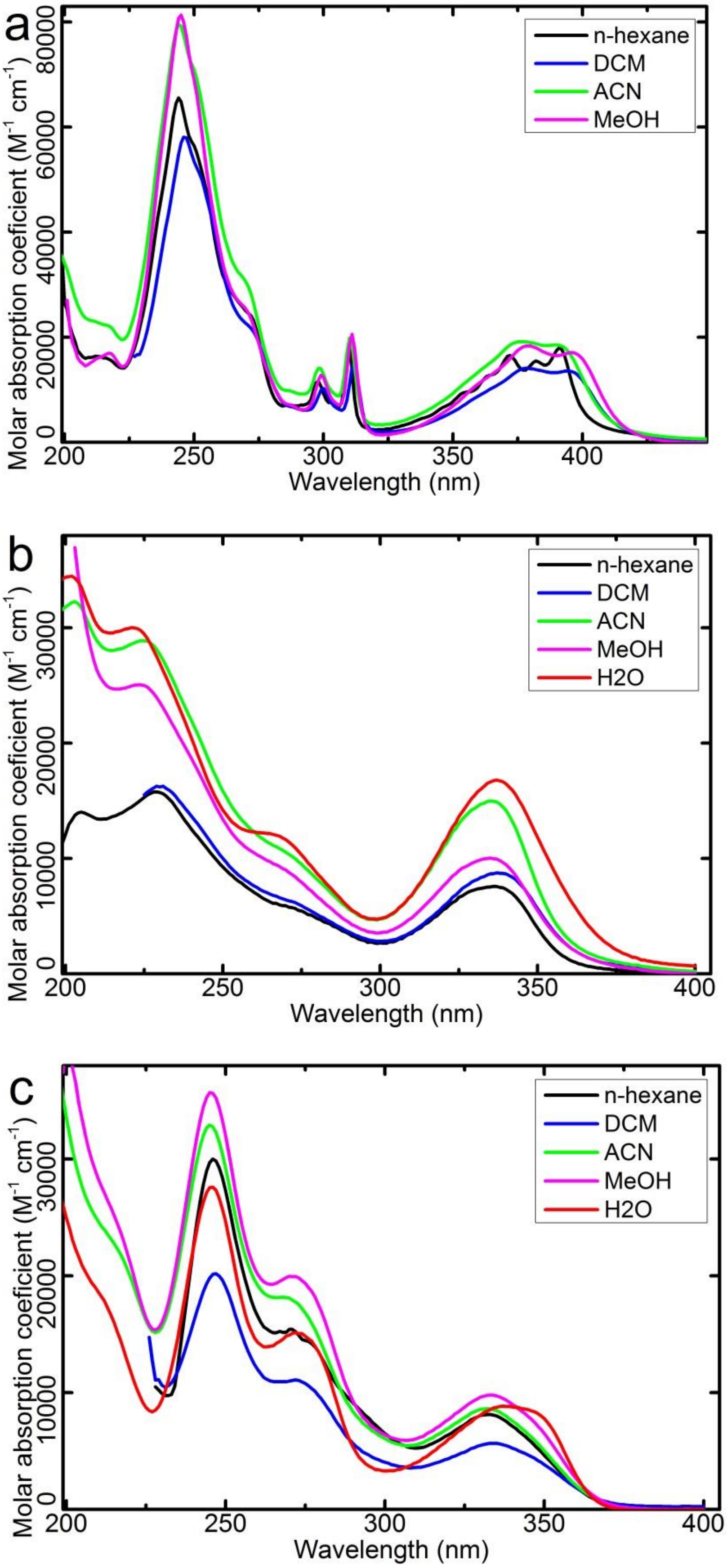

Figure S1. Absorption spectra of **PQPhen** (a), **PQ2Py** (b) and **Q2Py** (c) in solvents.

Table S4. Wavelength of the **i**th absorption band maximum, **λ_i**, and molar absorption coefficient at that wavelength, **$\varepsilon(\lambda_i)$**, and Stokes shift (**SS**) for **PQPhen**, **PQ2Py** and **Q2Py** in solvents.

**Compound**	**Solvent**	λ_1	$\varepsilon(\lambda_1)$	λ_2	$\varepsilon(\lambda_2)$	λ_3	$\varepsilon(\lambda_3)$	**SS**
		nm	$M^{-1}\cdot cm^{-1}$	nm	$M^{-1}\cdot cm^{-1}$	nm	$M^{-1}\cdot cm^{-1}$	cm^{-1}
PQPhen	*n*-hexane	391	17964	310	18695	244	65562	258
	DCM	394	13560	312	15065	248	58112	1284
	ACN	390	18544	310	19878	244	79580	4364
	MeOH	396	17062	311	20539	245	81320	3930
PQ2Py	*n*-hexane	335	7580	272	5734	229	15774	7921
	DCM	337	8747	273	6173	230	16280	8214
	ACN	335	14975	270	10547	224	28877	8619
	MeOH	335	10026	269	9117	224	25054	10357
	H_2O	337	16810	265	12155	221	30012	8487
Q2Py	*n*-hexane	333	8125	271	15400	246	30000	5216
	DCM	335	5659	272	11085	247	20185	5037
	ACN	333	8620	268	18167	245	32910	5216
	MeOH	333	9460	271	19600	245	35345	4841
	H_2O	338	8850	272	16020	246	30208	4010

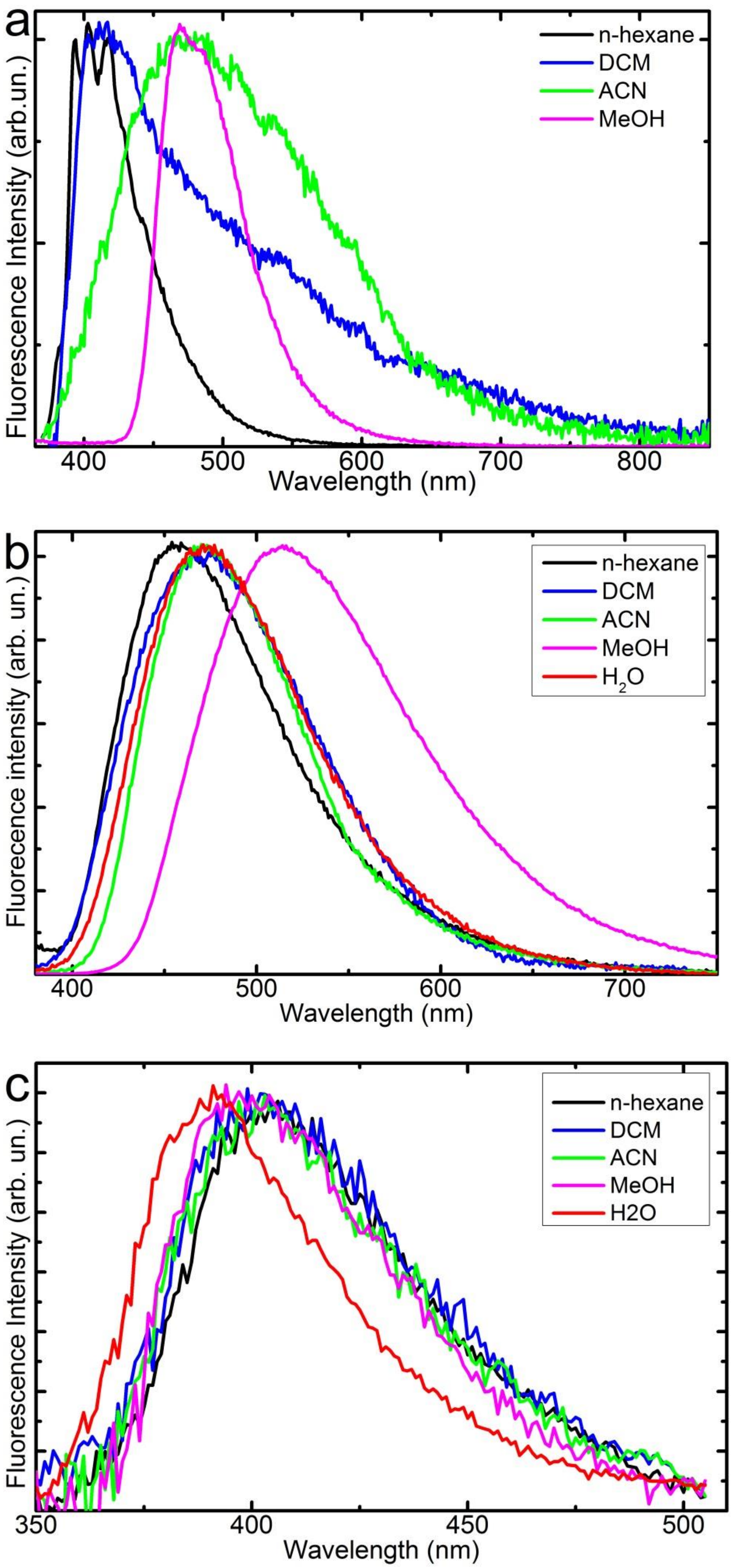

Figure S2. Normalized fluorescence spectra of (a) **PQPhen**, (b) **PQ2Py** and (c) **Q2Py** in solvents.

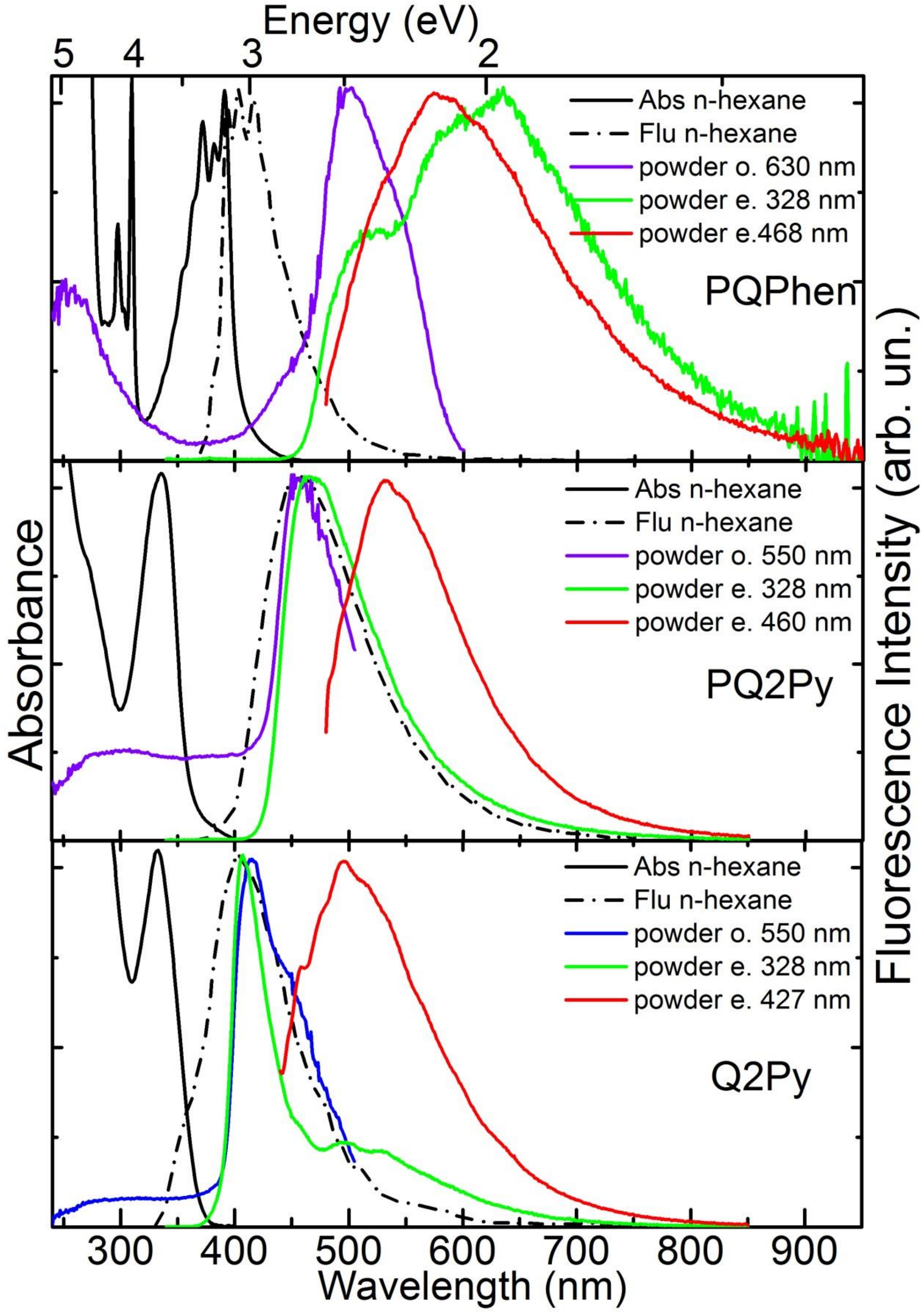

Figure S3. Normalized fluorescence excitation spectra (blue) and fluorescence spectra (green and red lines) of **PQPhen** (top), **PQ2Py** (middle) and **Q2Py** (bottom panel) in powder compared to absorption (black solid line) and fluorescence in *n*-hexane (black dot-dashed line). Legend specifies wavelength of observation for fluorescence excitation spectra and wavelength of excitation for fluorescence spectra in powder.

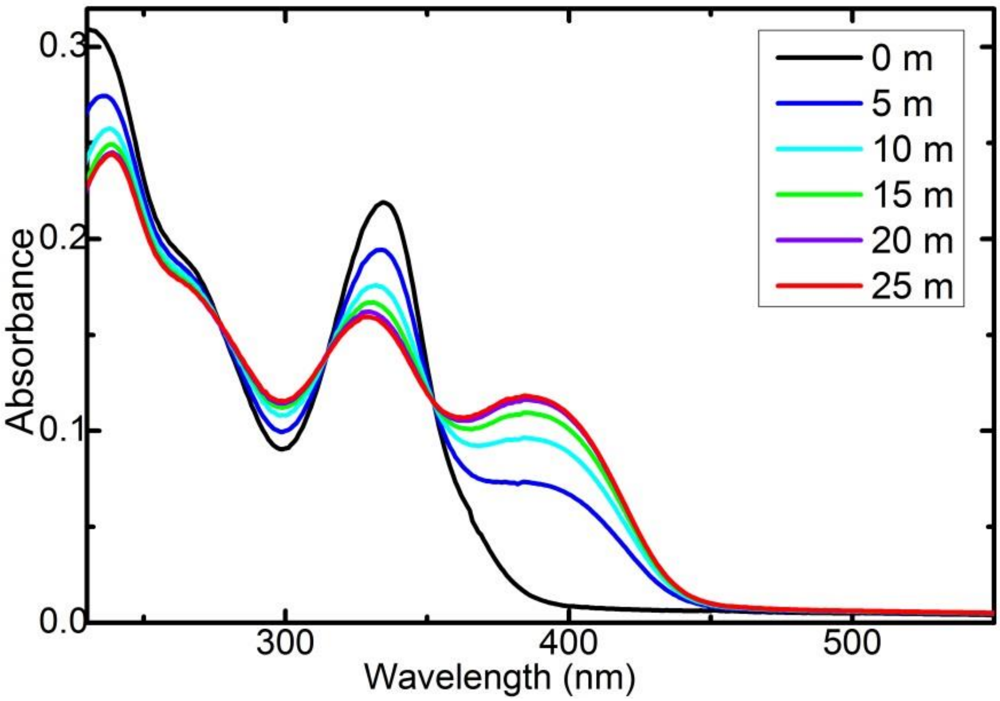

Figure S4. Effect of 375 nm photoirradiation on absorption spectra of **PQ2Py** in methanol. The legend specifies time of irradiation in minutes.

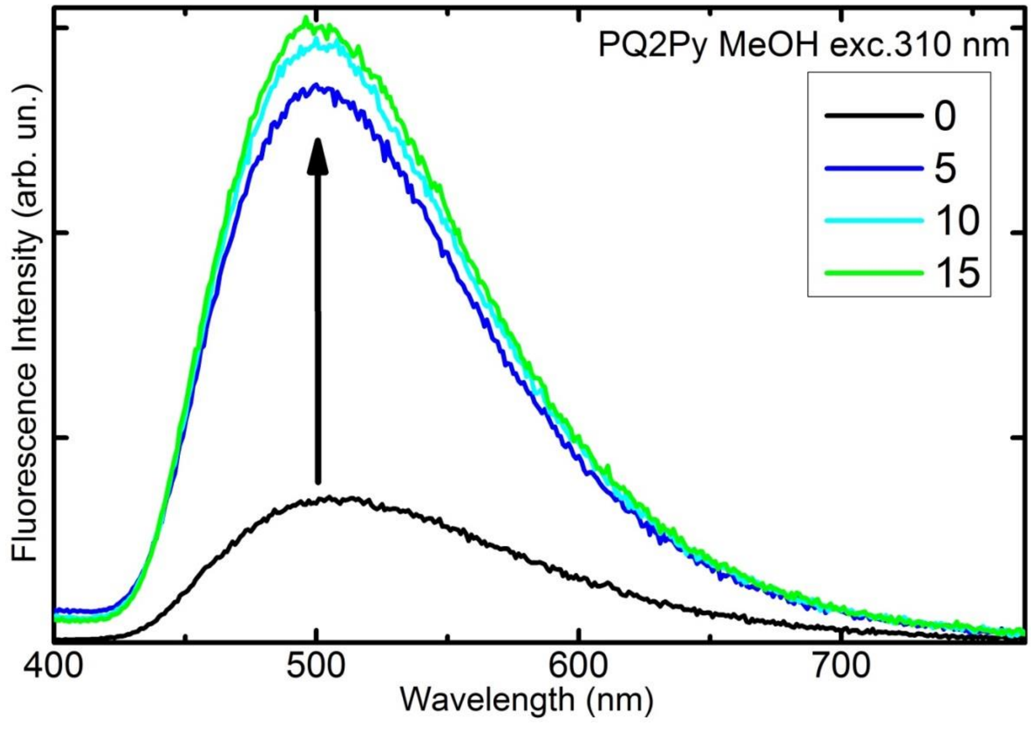

Figure S5. Effect of 375 nm photo-irradiation on fluorescence spectra of **PQ2Py** in methanol. Spectra obtained with 310 nm excitation. The legend specifies time of irradiation in minutes. Arrow visualizes the increase of fluorescence intensity with time of the irradiation.

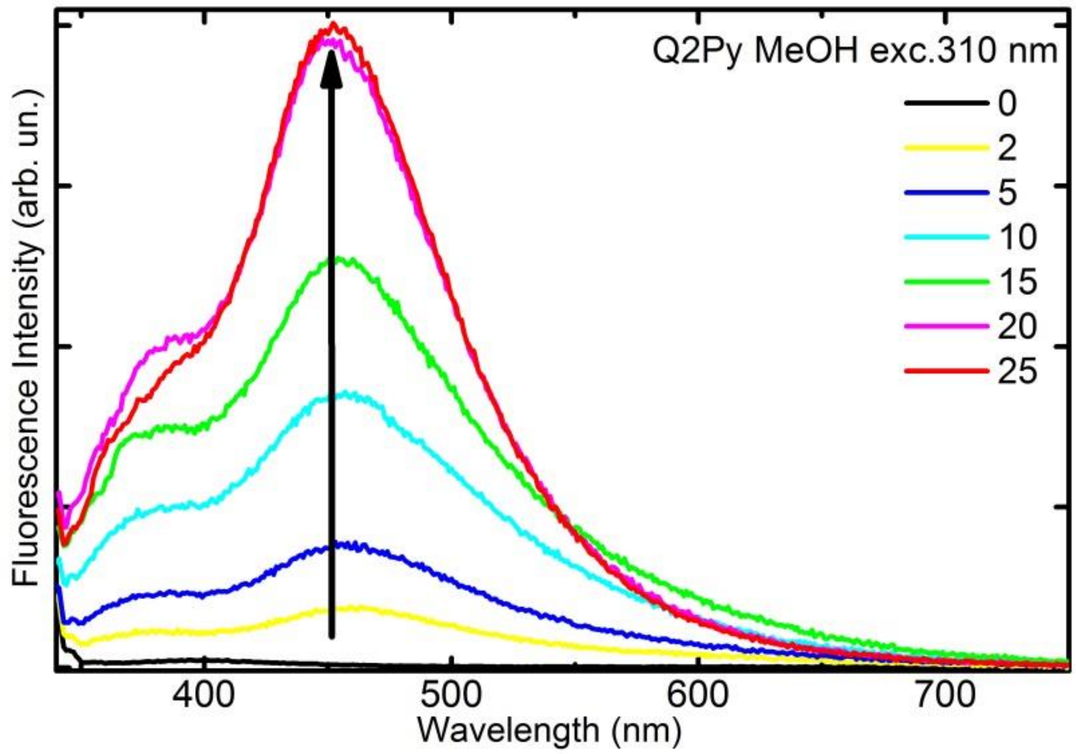

Figure S6. Effect of 277 nm photo-irradiation on fluorescence spectra of **Q2Py** in methanol. Spectra obtained with 310 nm excitation. The legend specifies time of irradiation in minutes. Arrow visualizes the increase of fluorescence intensity with time of the irradiation.

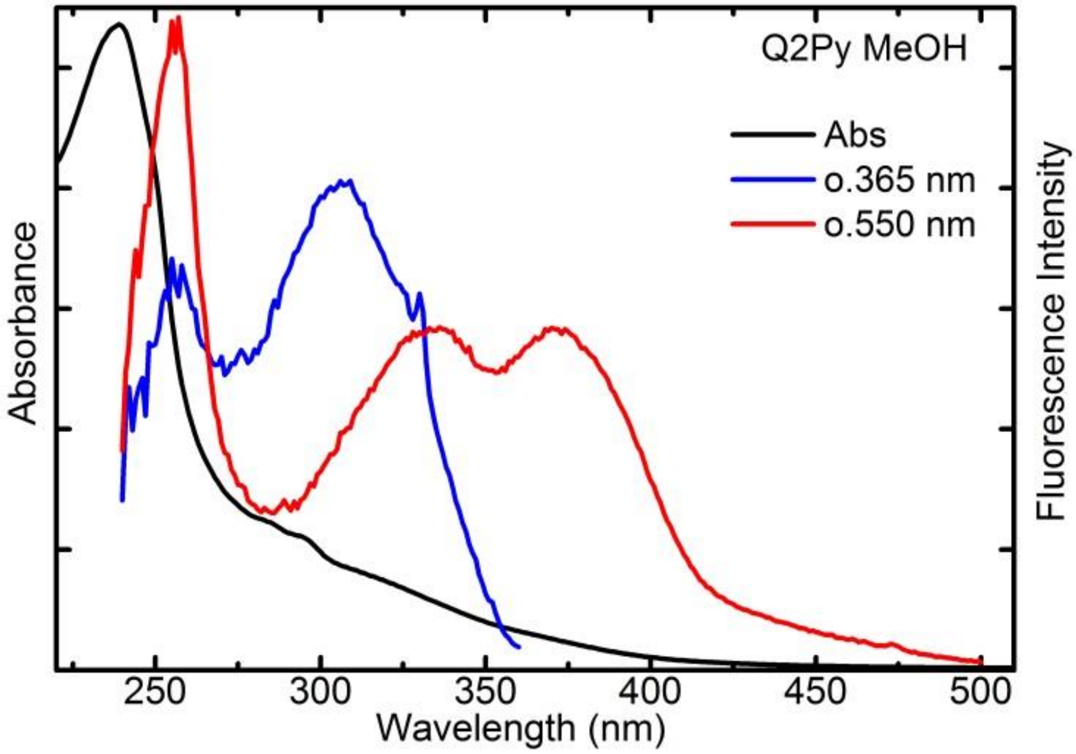

Figure S7. Absorption and fluorescence excitation spectra (observed at 365 and 550 nm) of **Q2Py** in methanol recorded after 25 minutes of irradiation at 277 nm.

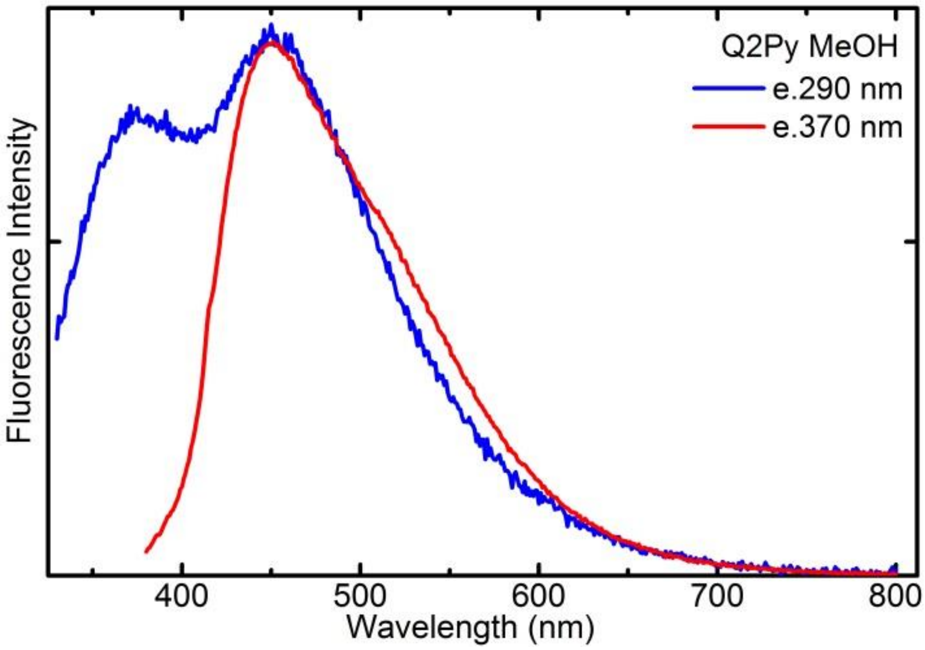

Figure S8. Fluorescence spectra (excitation at 290 and 370 nm) of **Q2Py** in methanol recorded after 25 minutes of irradiation at 277 nm.

5.2 Electrochemical characterization

Electrochemical reduction of quinoxaline leads to its di-protonated form.[1-3] Consequently, our motivation to perform electrochemical characterizations on our quinoxaline derivatives to investigate whether their varied structures affect the reduction processes. In aprotic acetonitrile a pair of distinct redox waves is always observed at -1.60 / -1.51 , -1.49 / -1.42 and -1.49 / -1.42V for **PQPhen**, **PQ2Py** and **Q2Py** respectively (Figure SF15), proving occurrence of one reversible reduction to radical anion. For **PQPhen** and **PQ2Py** another reduction also occurs, but irreversible, suggesting the formed dianion reacts with solvent or electrolyte. In contrast, the radical anion turns up to be very stable. In protic methanol the waves for reduction and oxidation within a pair are more separated (-1.35 / -1.02, -1.34 / -0.98 and -1.68 / -1.12 V respectively) pointing at different mechanism, reduction and electrochemical hydrogenation are the likely candidates. The increased separation within a pair may suggest a re-aromatization is related with this cyclic process. For **PQ2Py** and **Q2Py** an additional lonely reduction wave is visible at -2.09 and -2.25 V respectively which again points at irreversible process with the dianion. In both environments **PQPhen** behavies a bit differently than **PQ2Py** and **PQ2Py**, which suggests that the extension of π-conjugated structure influences the radical anion and the electrochemical hydrogenation more than the acceptor elements. As this is the only difference between molecules, one may state that all compounds have similar redox property.

[1] D. van der Meer, D. Feil, *Electrochemical reduction of aza aromatics. Part I. Polarographic reduction of aromatic di-aza compounds and correlation of results with H.M.O. calculations*, *Recl. Trav. Chim. Pays-Bas.*, **1968**, 87, 746-754.

[2] D. van der Meer, *Electrochemical reduction of AZA aromatics: Part II. Protonation products of di-aza aromatics*, *Recl. Trav. Chim. Pays-Bas.*, **1969**, 88, 1361-1372.

[3] J. D. Milshtein, L. Su, C. Liou, A. F. Badel, F. R. Brushett, *Voltammetry study of quinoxaline in aqueous electrolytes*, *Electrochimica Acta*, **2015**, 180, 695-704.

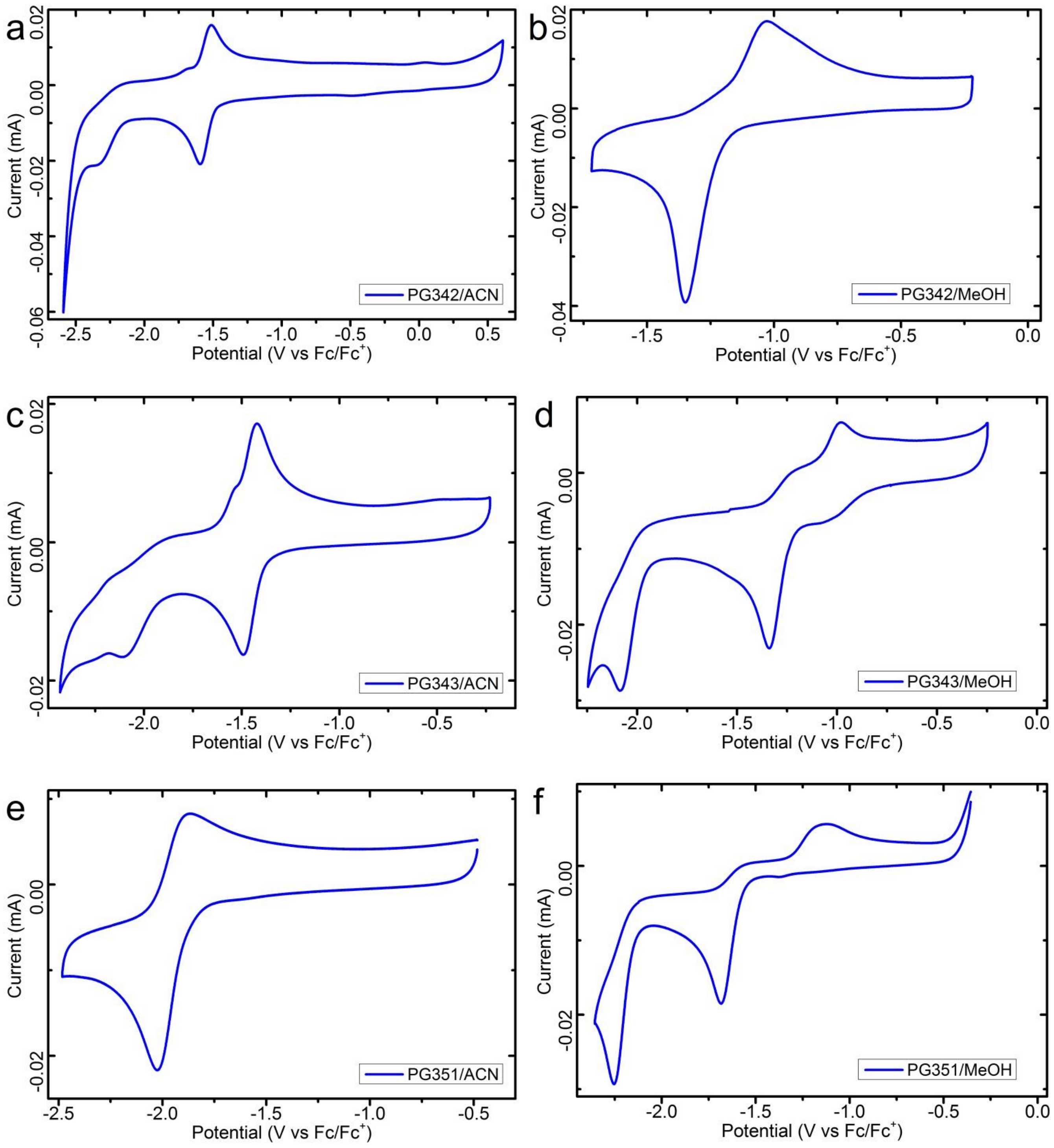

Figure S9. Cyclic voltammetry (CV) of the **PQPhen** (a, b), **PQ2Py** (c, d), and **Q2Py** (e, f), compounds in 0.1 M [NBu$_4$][PF$_6$] acetonitrile (ACN, a, c and e) and methanol (MeOH, b, d and f) solutions. Scan rate was 200 mV s^{-1} with a 3 mm glassy carbon working electrode. The concentration of the compounds was within 5×10^{-4} to 10^{-3} M range. The 0 V corresponds to the Fc/Fc$^+$ redox couple.

5.3 The ^{1}H NMR results

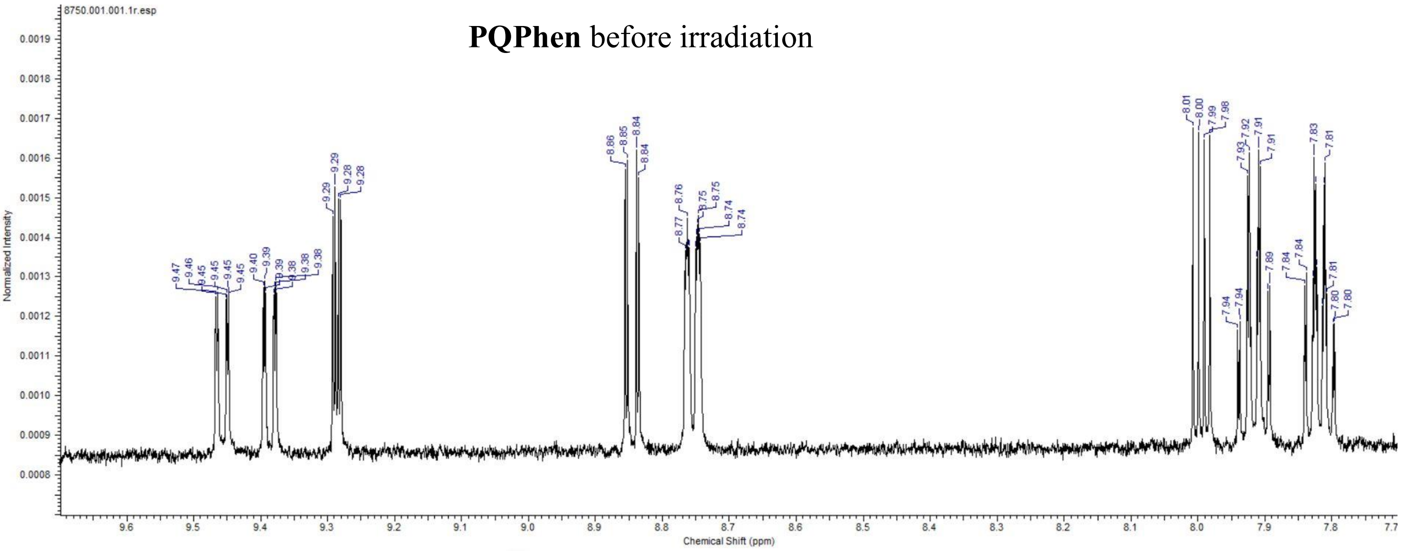

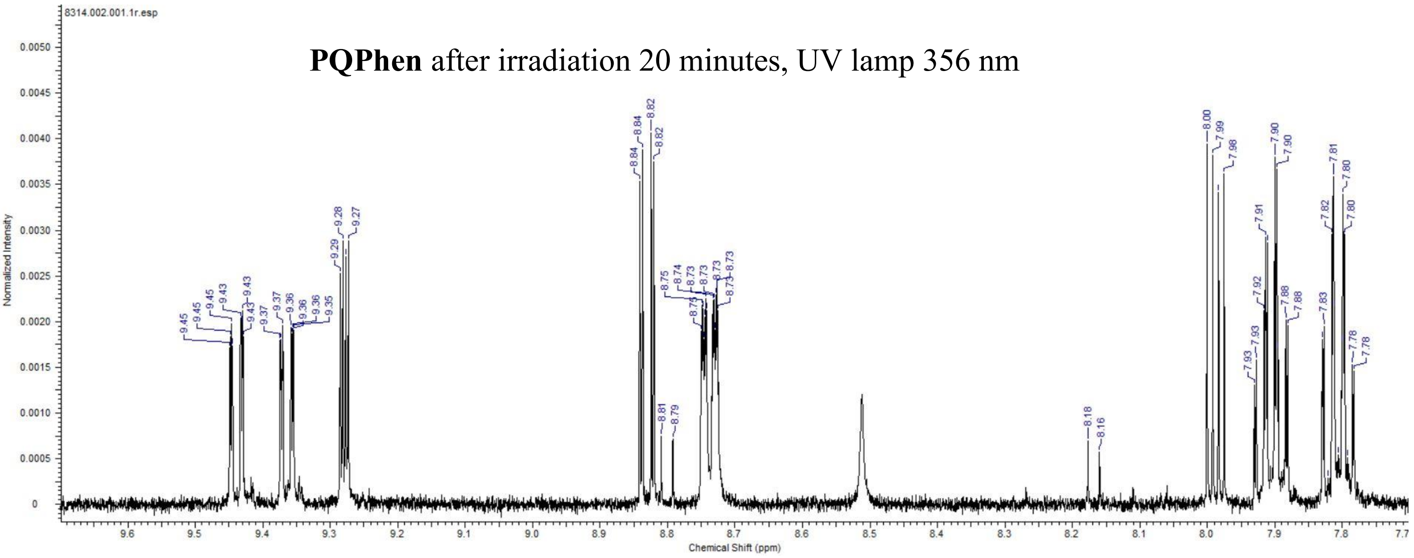

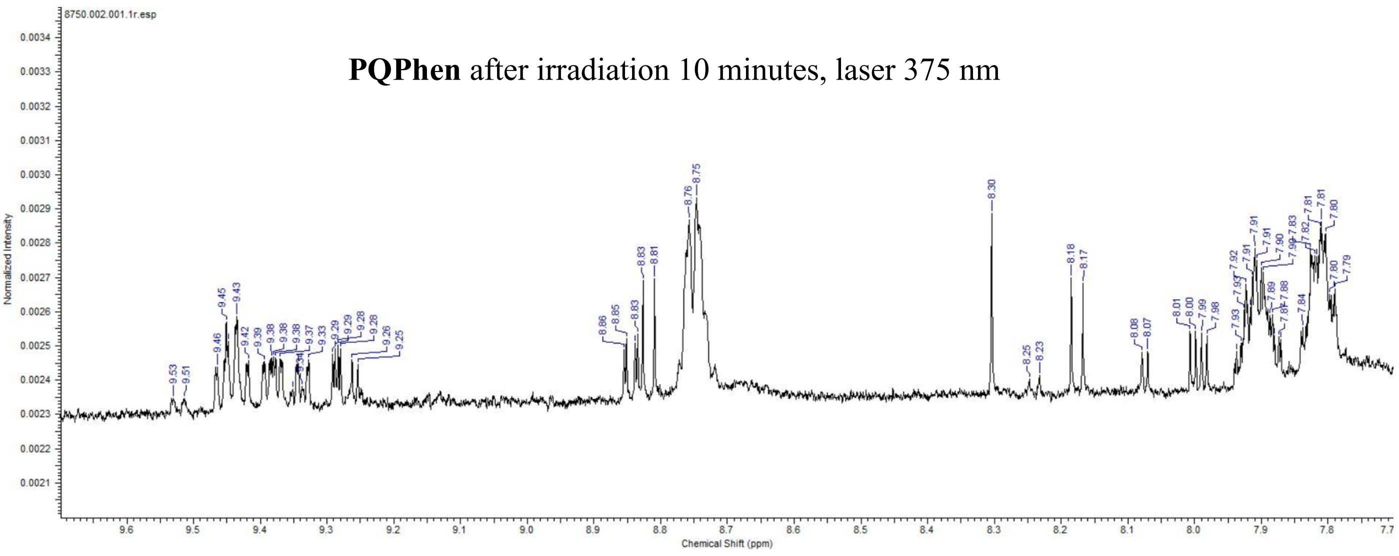

Figure S10. The ^{1}H spectra of diluted **PQPhen** in methanol-d_4 before and after irradiation.

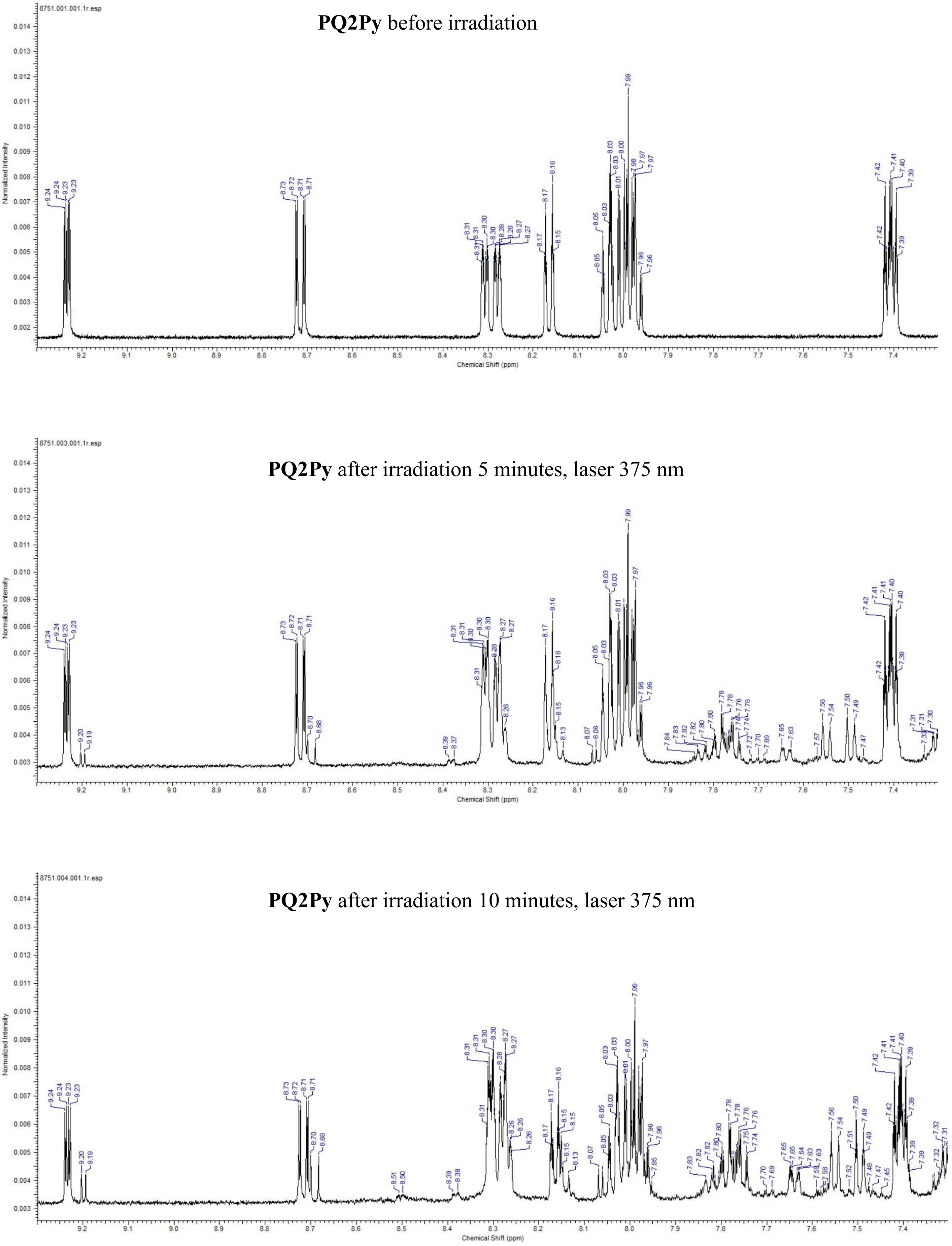

Figure S11. The ^{1}H spectra of diluted **PQ2Py** in methanol-d_4 before and after irradiation.

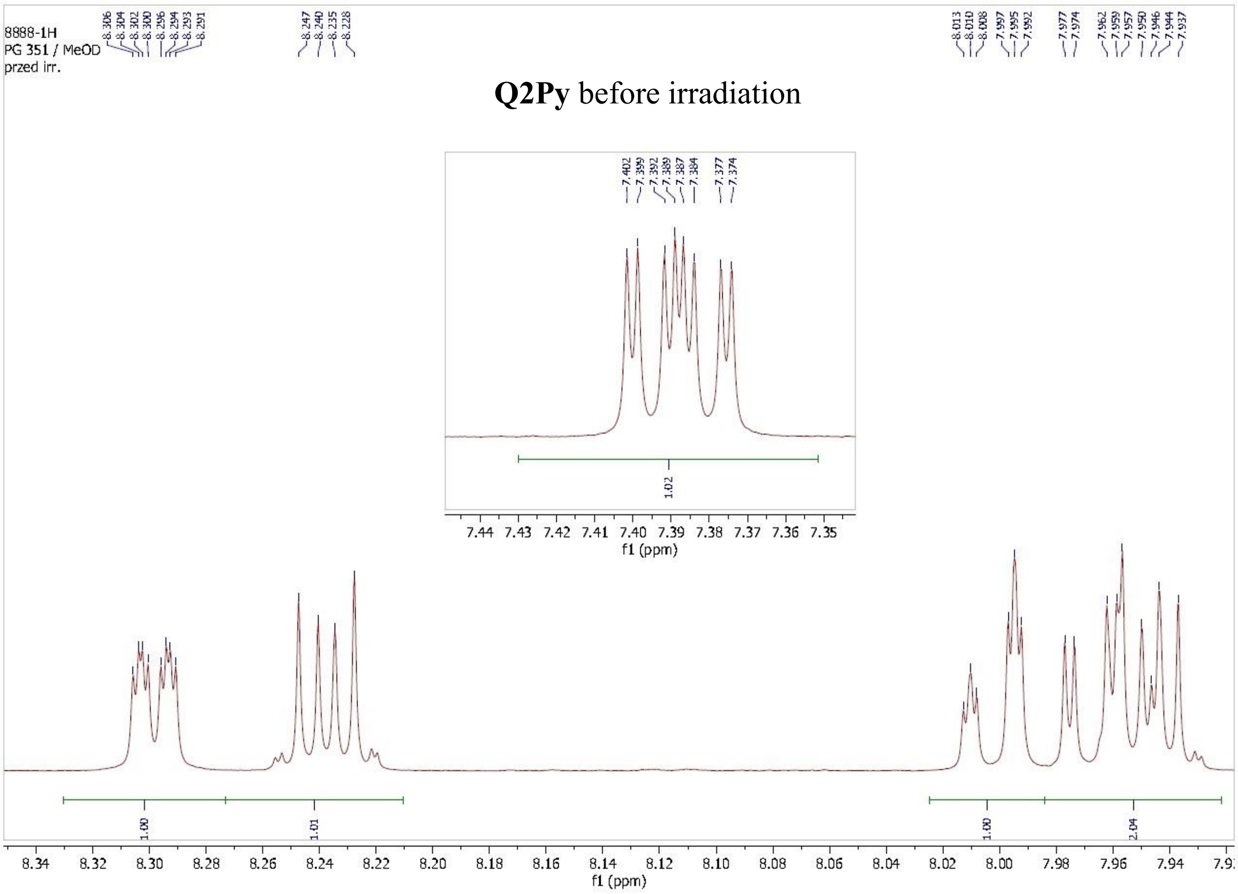

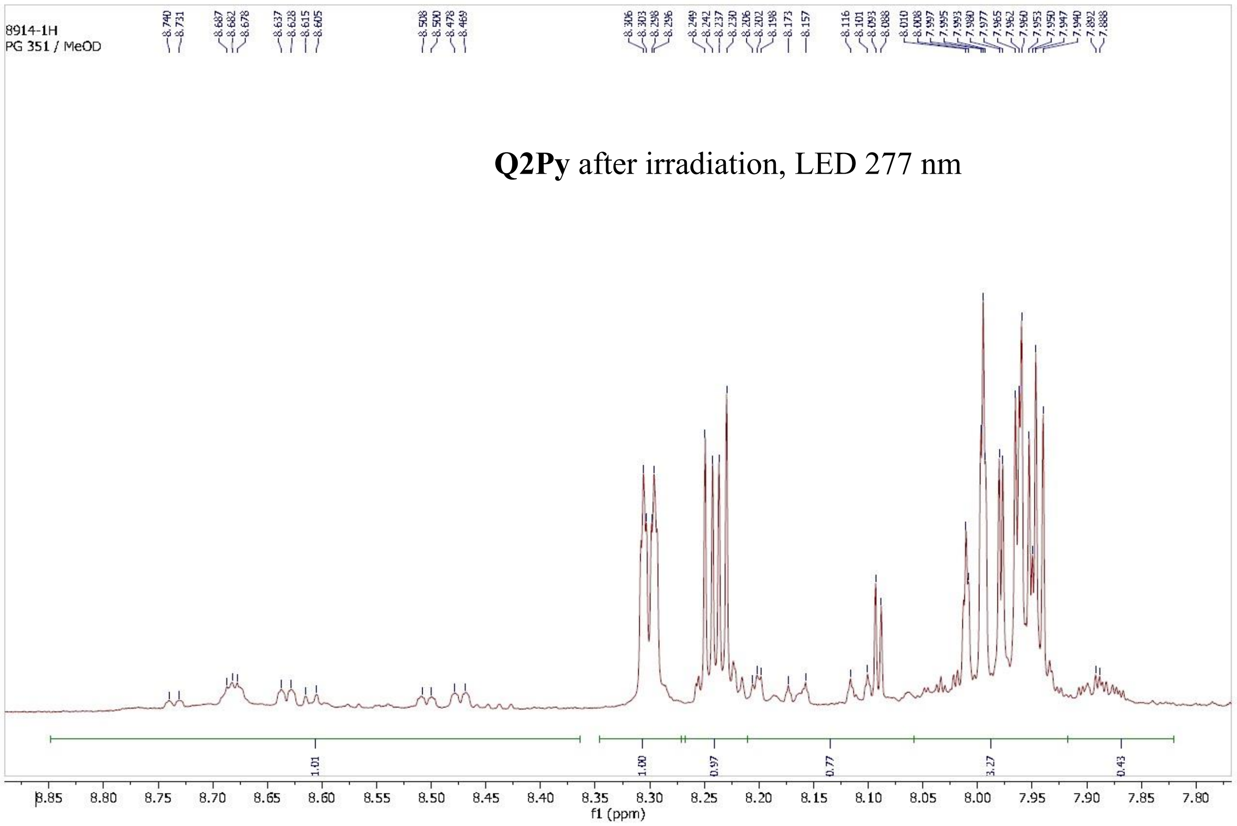

Figure S12. The ^{1}H spectra of diluted **Q2Py** in methanol-d_4 before and after irradiation.

5.4 Mass Spectroscopy results

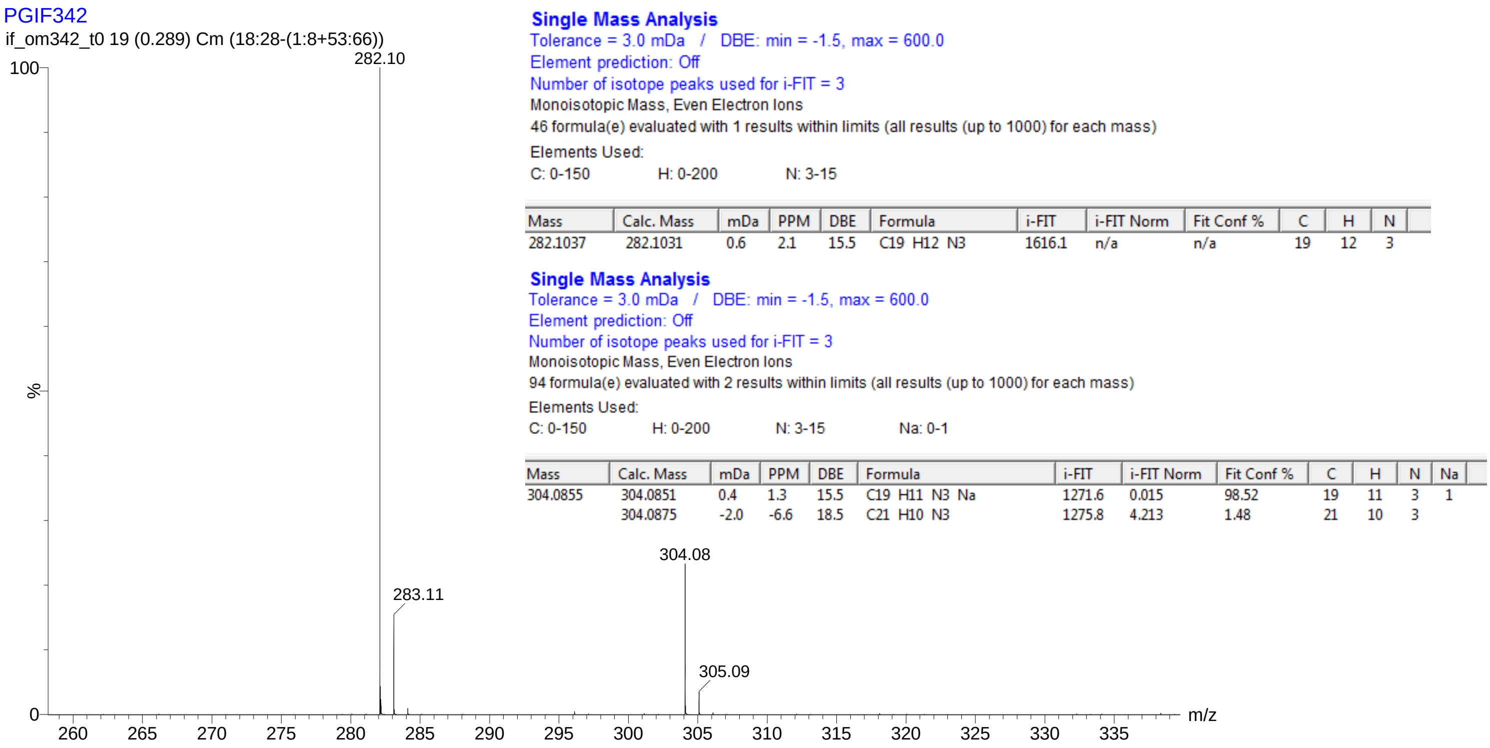

Single Mass Analysis
Tolerance = 3.0 mDa / DBE: min = -1.5, max = 600.0
Element prediction: Off
Number of isotope peaks used for i-FIT = 3
Monoisotopic Mass, Even Electron Ions
46 formula(e) evaluated with 1 results within limits (all results (up to 1000) for each mass)
Elements Used:
C: 0-150 H: 0-200 N: 3-15

Mass	Calc. Mass	mDa	PPM	DBE	Formula	i-FIT	i-FIT Norm	Fit Conf %	C	H	N
282.1037	282.1031	0.6	2.1	15.5	C19 H12 N3	1616.1	n/a	n/a	19	12	3

Single Mass Analysis
Tolerance = 3.0 mDa / DBE: min = -1.5, max = 600.0
Element prediction: Off
Number of isotope peaks used for i-FIT = 3
Monoisotopic Mass, Even Electron Ions
94 formula(e) evaluated with 2 results within limits (all results (up to 1000) for each mass)
Elements Used:
C: 0-150 H: 0-200 N: 3-15 Na: 0-1

Mass	Calc. Mass	mDa	PPM	DBE	Formula	i-FIT	i-FIT Norm	Fit Conf %	C	H	N	Na
304.0855	304.0851	0.4	1.3	15.5	C19 H11 N3 Na	1271.6	0.015	98.52	19	11	3	1
	304.0875	-2.0	-6.6	18.5	C21 H10 N3	1275.8	4.213	1.48	21	10	3	

Figure S13. The extended mass spectrum of the sample **PQPhen** before laser irradiation and elemental prediction tables for the m/z = 282.10 [**PQPhen** +H]$^+$ and for the m/z = 304.08 [**PQPhen** +Na]$^+$.

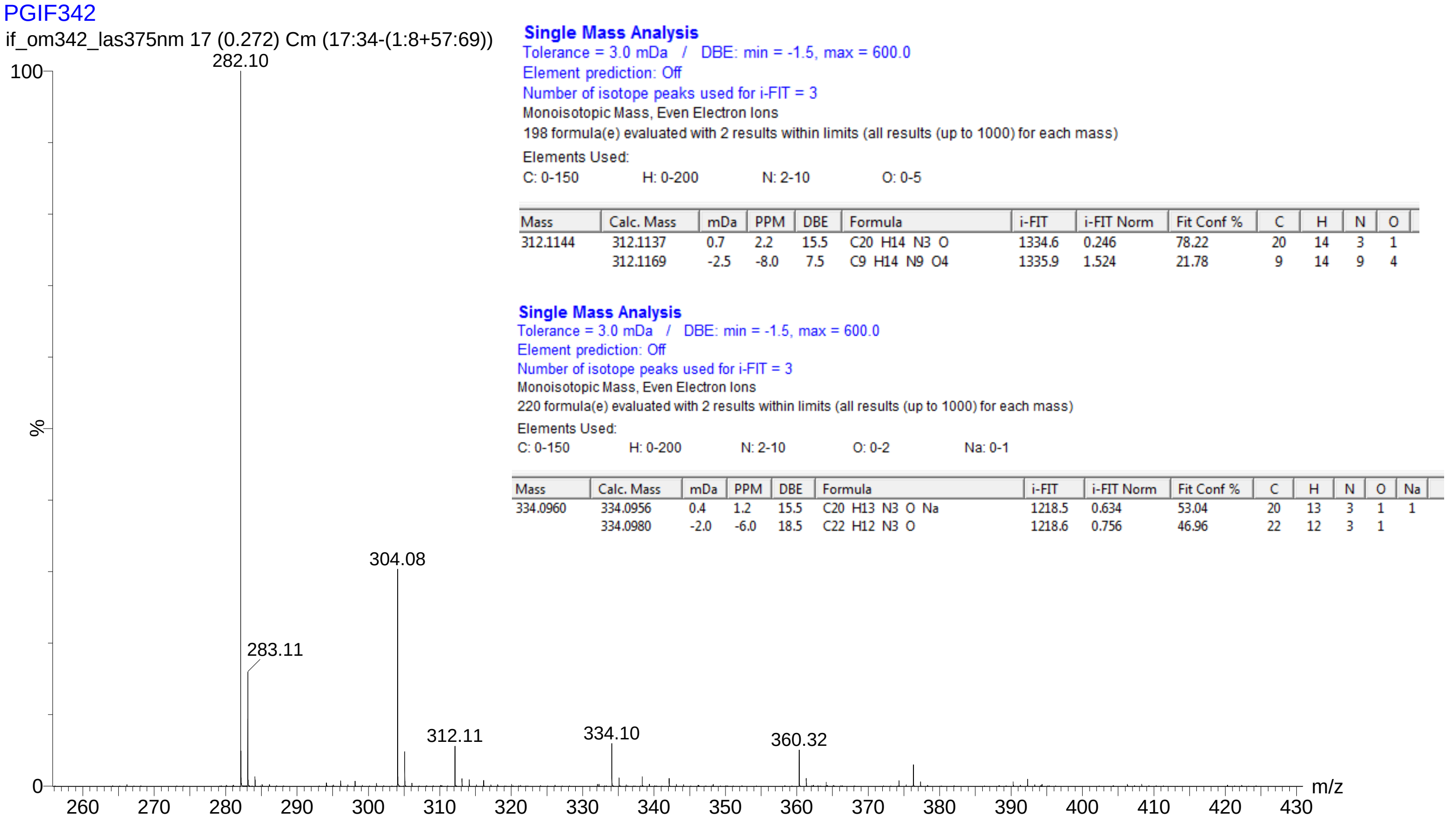

Single Mass Analysis
Tolerance = 3.0 mDa / DBE: min = -1.5, max = 600.0
Element prediction: Off
Number of isotope peaks used for i-FIT = 3
Monoisotopic Mass, Even Electron Ions
198 formula(e) evaluated with 2 results within limits (all results (up to 1000) for each mass)
Elements Used:
C: 0-150 H: 0-200 N: 2-10 O: 0-5

Mass	Calc. Mass	mDa	PPM	DBE	Formula	i-FIT	i-FIT Norm	Fit Conf %	C	H	N	O
312.1144	312.1137	0.7	2.2	15.5	C20 H14 N3 O	1334.6	0.246	78.22	20	14	3	1
	312.1169	-2.5	-8.0	7.5	C9 H14 N9 O4	1335.9	1.524	21.78	9	14	9	4

Single Mass Analysis
Tolerance = 3.0 mDa / DBE: min = -1.5, max = 600.0
Element prediction: Off
Number of isotope peaks used for i-FIT = 3
Monoisotopic Mass, Even Electron Ions
220 formula(e) evaluated with 2 results within limits (all results (up to 1000) for each mass)
Elements Used:
C: 0-150 H: 0-200 N: 2-10 O: 0-2 Na: 0-1

Mass	Calc. Mass	mDa	PPM	DBE	Formula	i-FIT	i-FIT Norm	Fit Conf %	C	H	N	O	Na
334.0960	334.0956	0.4	1.2	15.5	C20 H13 N3 O Na	1218.5	0.634	53.04	20	13	3	1	1
	334.0980	-2.0	-6.0	18.5	C22 H12 N3 O	1218.6	0.756	46.96	22	12	3	1	

Figure S14. The extended mass spectrum of the sample **PQPhen** after 405 nm laser irradiation and elemental prediction tables for the m/z = 312.11 [(**PQPhen**+OCH_3)+H]$^+$ and the m/z = 334.10 [(**PQPhen**+OCH_3)+Na]$^+$.

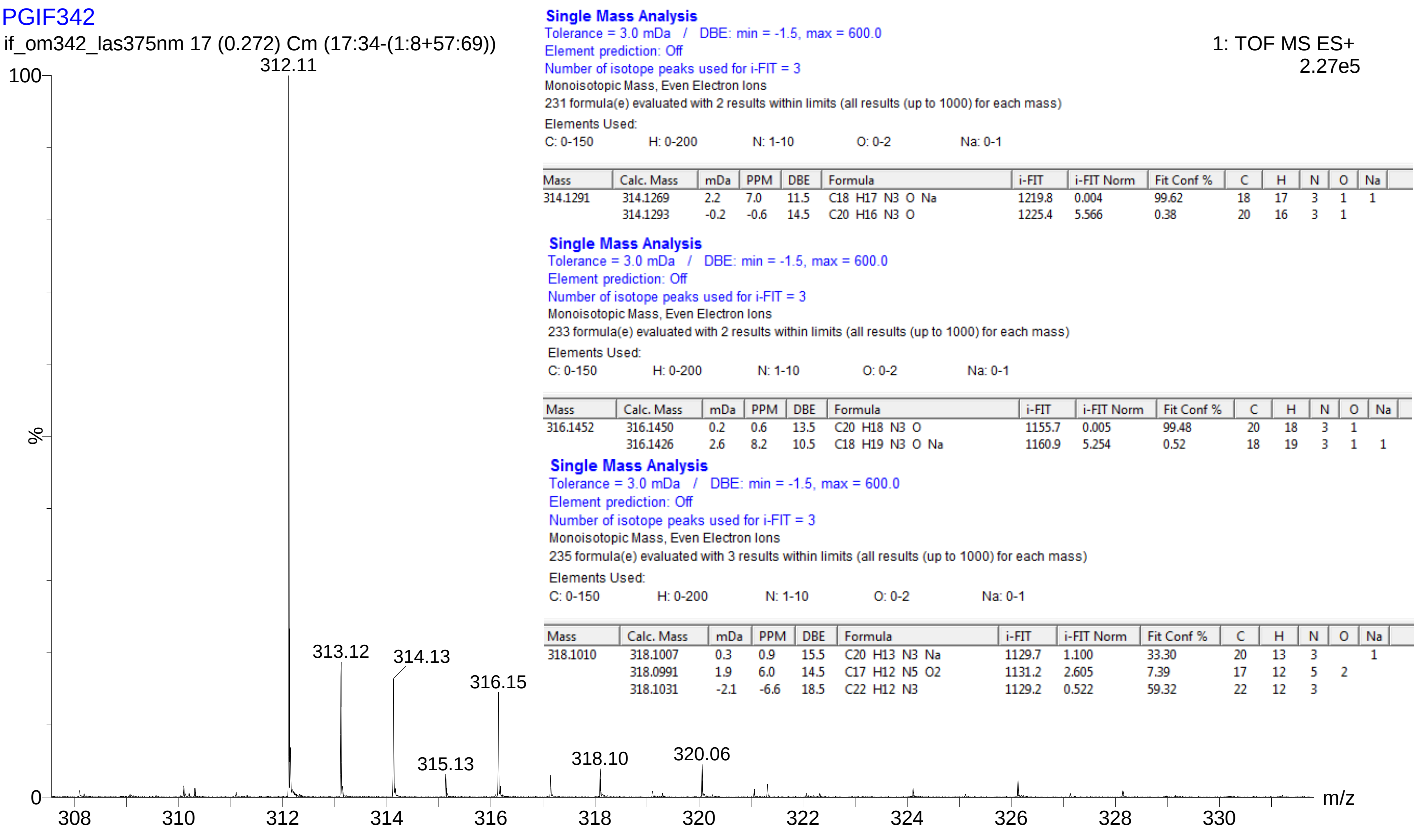

Single Mass Analysis
Tolerance = 3.0 mDa / DBE: min = -1.5, max = 600.0
Element prediction: Off
Number of isotope peaks used for i-FIT = 3
Monoisotopic Mass, Even Electron Ions
231 formula(e) evaluated with 2 results within limits (all results (up to 1000) for each mass)
Elements Used:
C: 0-150 H: 0-200 N: 1-10 O: 0-2 Na: 0-1

Mass	Calc. Mass	mDa	PPM	DBE	Formula	i-FIT	i-FIT Norm	Fit Conf %	C	H	N	O	Na
314.1291	314.1269	2.2	7.0	11.5	C18 H17 N3 O Na	1219.8	0.004	99.62	18	17	3	1	1
	314.1293	-0.2	-0.6	14.5	C20 H16 N3 O	1225.4	5.566	0.38	20	16	3	1	

Single Mass Analysis
Tolerance = 3.0 mDa / DBE: min = -1.5, max = 600.0
Element prediction: Off
Number of isotope peaks used for i-FIT = 3
Monoisotopic Mass, Even Electron Ions
233 formula(e) evaluated with 2 results within limits (all results (up to 1000) for each mass)
Elements Used:
C: 0-150 H: 0-200 N: 1-10 O: 0-2 Na: 0-1

Mass	Calc. Mass	mDa	PPM	DBE	Formula	i-FIT	i-FIT Norm	Fit Conf %	C	H	N	O	Na
316.1452	316.1450	0.2	0.6	13.5	C20 H18 N3 O	1155.7	0.005	99.48	20	18	3	1	
	316.1426	2.6	8.2	10.5	C18 H19 N3 O Na	1160.9	5.254	0.52	18	19	3	1	1

Single Mass Analysis
Tolerance = 3.0 mDa / DBE: min = -1.5, max = 600.0
Element prediction: Off
Number of isotope peaks used for i-FIT = 3
Monoisotopic Mass, Even Electron Ions
235 formula(e) evaluated with 3 results within limits (all results (up to 1000) for each mass)
Elements Used:
C: 0-150 H: 0-200 N: 1-10 O: 0-2 Na: 0-1

Mass	Calc. Mass	mDa	PPM	DBE	Formula	i-FIT	i-FIT Norm	Fit Conf %	C	H	N	O	Na
318.1010	318.1007	0.3	0.9	15.5	C20 H13 N3 Na	1129.7	1.100	33.30	20	13	3		1
	318.0991	1.9	6.0	14.5	C17 H12 N5 O2	1131.2	2.605	7.39	17	12	5	2	
	318.1031	-2.1	-6.6	18.5	C22 H12 N3	1129.2	0.522	59.32	22	12	3		

Figure S15. The extended mass spectrum of the sample **PQPhen** after 405 nm laser irradiation and elemental prediction tables for the m/z = 314.13 [(**PQPhen**+2H+OCH_3)+H]$^+$ and for the m/z = 316.15 [(**PQPhen**+4H+OCH_3)+H]$^+$ and for the m/z = 320.06 [(**PQPhen**+2H+CH_3)+Na]$^+$.

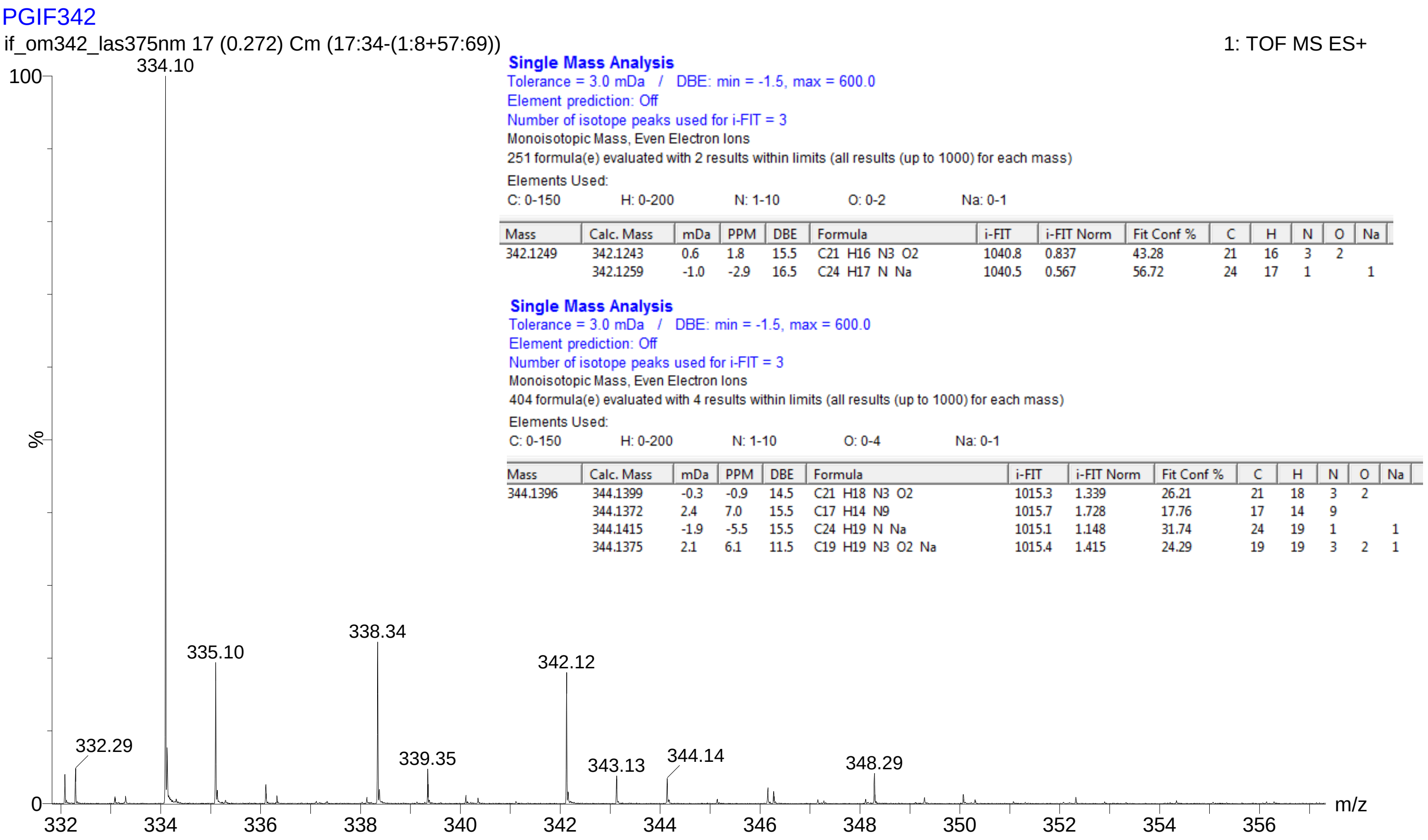

Single Mass Analysis
Tolerance = 3.0 mDa / DBE: min = -1.5, max = 600.0
Element prediction: Off
Number of isotope peaks used for i-FIT = 3
Monoisotopic Mass, Even Electron Ions
251 formula(e) evaluated with 2 results within limits (all results (up to 1000) for each mass)
Elements Used:
C: 0-150 H: 0-200 N: 1-10 O: 0-2 Na: 0-1

Mass	Calc. Mass	mDa	PPM	DBE	Formula	i-FIT	i-FIT Norm	Fit Conf %	C	H	N	O	Na
342.1249	342.1243	0.6	1.8	15.5	C21 H16 N3 O2	1040.8	0.837	43.28	21	16	3	2	
	342.1259	-1.0	-2.9	16.5	C24 H17 N Na	1040.5	0.567	56.72	24	17	1		1

Single Mass Analysis
Tolerance = 3.0 mDa / DBE: min = -1.5, max = 600.0
Element prediction: Off
Number of isotope peaks used for i-FIT = 3
Monoisotopic Mass, Even Electron Ions
404 formula(e) evaluated with 4 results within limits (all results (up to 1000) for each mass)
Elements Used:
C: 0-150 H: 0-200 N: 1-10 O: 0-4 Na: 0-1

Mass	Calc. Mass	mDa	PPM	DBE	Formula	i-FIT	i-FIT Norm	Fit Conf %	C	H	N	O	Na
344.1396	344.1399	-0.3	-0.9	14.5	C21 H18 N3 O2	1015.3	1.339	26.21	21	18	3	2	
	344.1372	2.4	7.0	15.5	C17 H14 N9	1015.7	1.728	17.76	17	14	9		
	344.1415	-1.9	-5.5	15.5	C24 H19 N Na	1015.1	1.148	31.74	24	19	1		1
	344.1375	2.1	6.1	11.5	C19 H19 N3 O2 Na	1015.4	1.415	24.29	19	19	3	2	1

Figure S16. The extended mass spectrum of the sample **PQPhen** after laser irradiation and elemental prediction table for the m/z = 342.12 [(**PQPhen**+2(OCH_3))+H]$^+$ and for the m/z= 344.15 [(**PQPhen**+2H+2(OCH_3))+H]$^+$.

Table S5. The proposed structure of some photoproducts found for **PQPhen** irradiated in methanol.

m/z	Structure
298.14	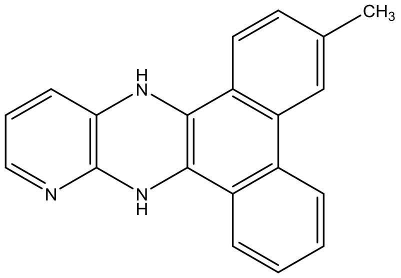<br>Chemical Formula: $C_{20}H_{15}N_3$<br>Exact Mass: 297,12660
314.13	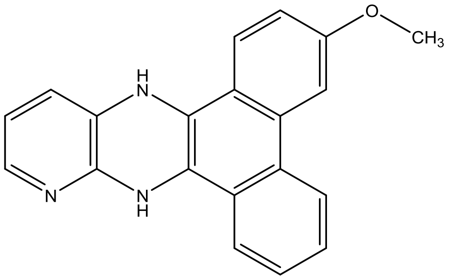<br>Chemical Formula: $C_{20}H_{15}N_3O$<br>Exact Mass: 313,12151
316.15	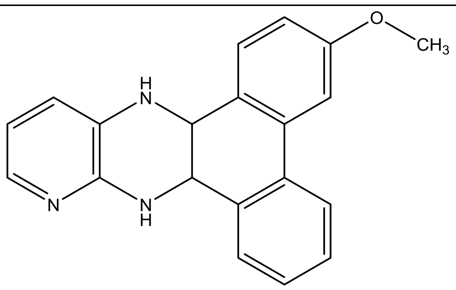<br>Chemical Formula: $C_{20}H_{17}N_3O$<br>Exact Mass: 315,13716<br>or<br>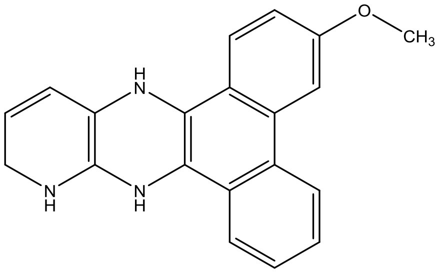<br>Chemical Formula: $C_{20}H_{17}N_3O$<br>Exact Mass: 315,13716
344.15	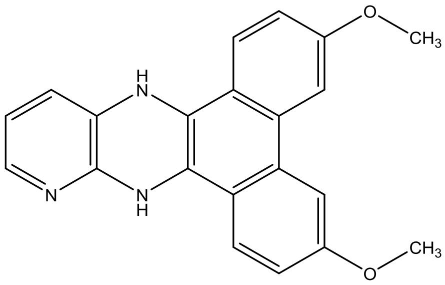<br>Chemical Formula: $C_{21}H_{17}N_3O_2$<br>Exact Mass: 343,13208

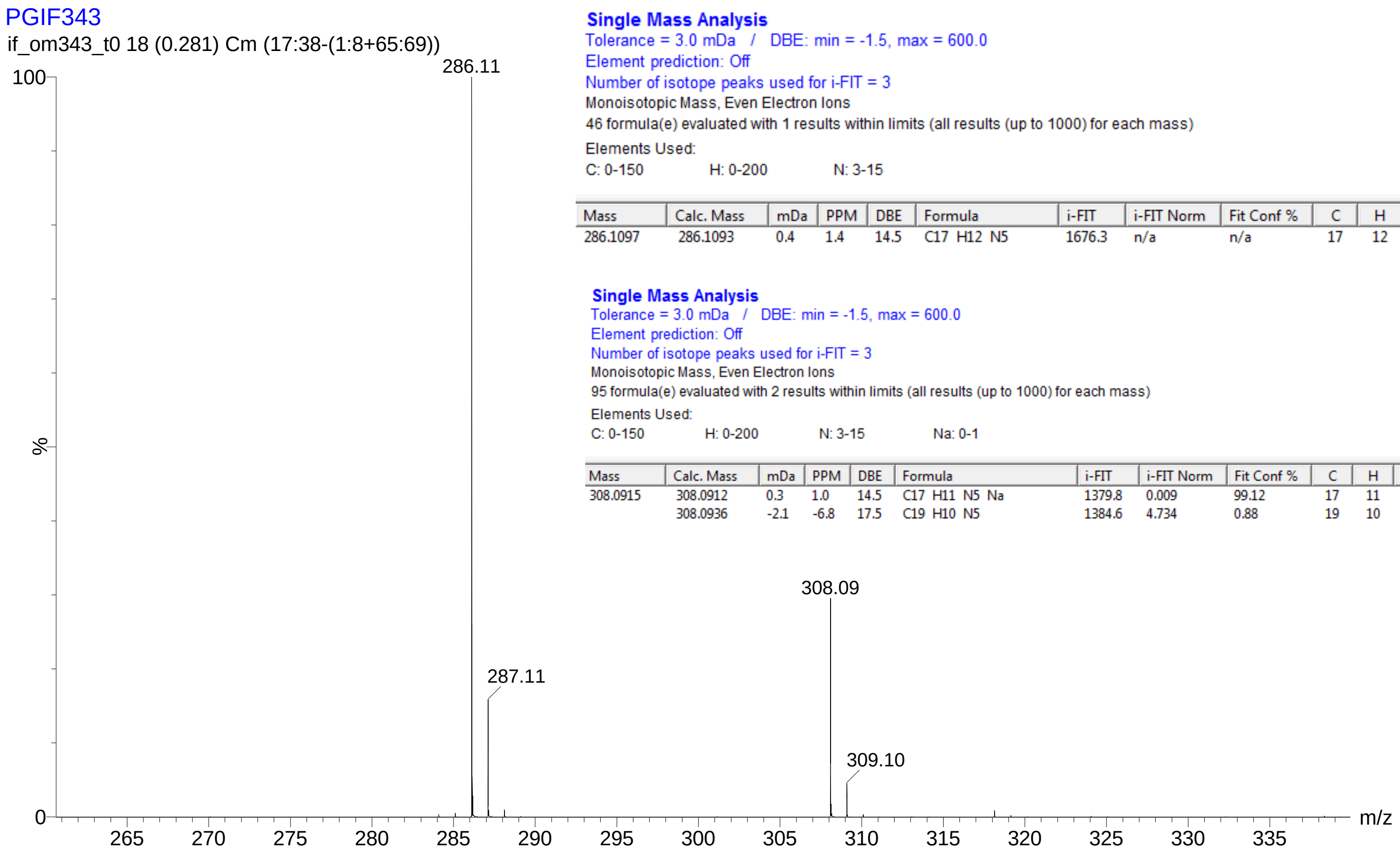

Single Mass Analysis
Tolerance = 3.0 mDa / DBE: min = -1.5, max = 600.0
Element prediction: Off
Number of isotope peaks used for i-FIT = 3
Monoisotopic Mass, Even Electron Ions
46 formula(e) evaluated with 1 results within limits (all results (up to 1000) for each mass)
Elements Used:
C: 0-150 H: 0-200 N: 3-15

Mass	Calc. Mass	mDa	PPM	DBE	Formula	i-FIT	i-FIT Norm	Fit Conf %	C	H	N
286.1097	286.1093	0.4	1.4	14.5	C17 H12 N5	1676.3	n/a	n/a	17	12	5

Single Mass Analysis
Tolerance = 3.0 mDa / DBE: min = -1.5, max = 600.0
Element prediction: Off
Number of isotope peaks used for i-FIT = 3
Monoisotopic Mass, Even Electron Ions
95 formula(e) evaluated with 2 results within limits (all results (up to 1000) for each mass)
Elements Used:
C: 0-150 H: 0-200 N: 3-15 Na: 0-1

Mass	Calc. Mass	mDa	PPM	DBE	Formula	i-FIT	i-FIT Norm	Fit Conf %	C	H	N	Na
308.0915	308.0912	0.3	1.0	14.5	C17 H11 N5 Na	1379.8	0.009	99.12	17	11	5	1
	308.0936	-2.1	-6.8	17.5	C19 H10 N5	1384.6	4.734	0.88	19	10	5	

Figure S17. The extended mass spectrum of the sample **PQ2Py** before laser irradiation and elemental prediction tables for the m/z = 286.11 [**PQ2Py**+H]$^+$ and for the m/z = 308.09 [**PQ2Py**+Na]$^+$.

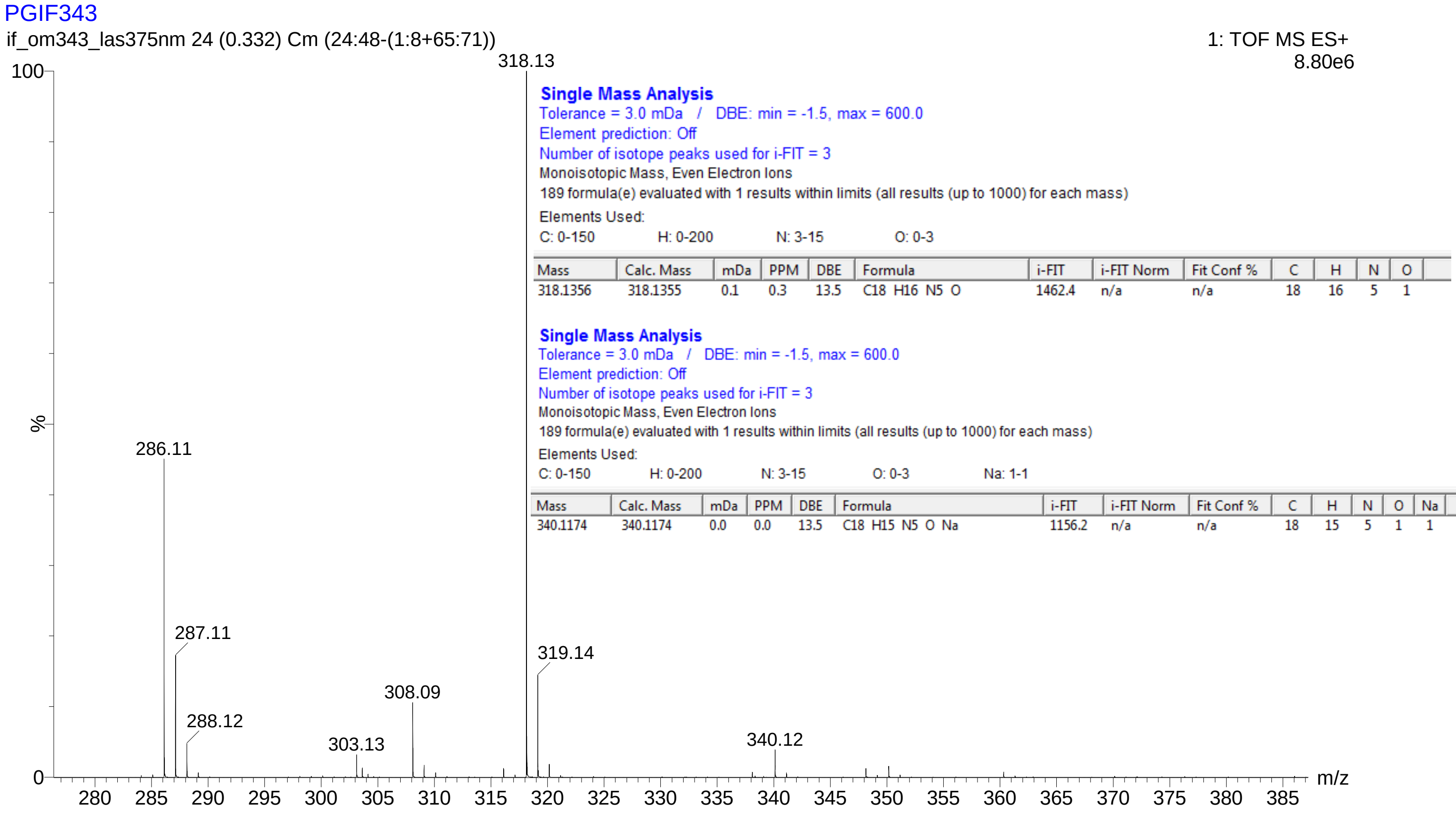

Single Mass Analysis
Tolerance = 3.0 mDa / DBE: min = -1.5, max = 600.0
Element prediction: Off
Number of isotope peaks used for i-FIT = 3
Monoisotopic Mass, Even Electron Ions
189 formula(e) evaluated with 1 results within limits (all results (up to 1000) for each mass)
Elements Used:
C: 0-150 H: 0-200 N: 3-15 O: 0-3

Mass	Calc. Mass	mDa	PPM	DBE	Formula	i-FIT	i-FIT Norm	Fit Conf %	C	H	N	O
318.1356	318.1355	0.1	0.3	13.5	C18 H16 N5 O	1462.4	n/a	n/a	18	16	5	1

Single Mass Analysis
Tolerance = 3.0 mDa / DBE: min = -1.5, max = 600.0
Element prediction: Off
Number of isotope peaks used for i-FIT = 3
Monoisotopic Mass, Even Electron Ions
189 formula(e) evaluated with 1 results within limits (all results (up to 1000) for each mass)
Elements Used:
C: 0-150 H: 0-200 N: 3-15 O: 0-3 Na: 1-1

Mass	Calc. Mass	mDa	PPM	DBE	Formula	i-FIT	i-FIT Norm	Fit Conf %	C	H	N	O	Na
340.1174	340.1174	0.0	0.0	13.5	C18 H15 N5 O Na	1156.2	n/a	n/a	18	15	5	1	1

Figure S18. The extended mass spectrum of the sample **PQ2Py** after 375 nm laser irradiation and elemental prediction tables for the m/z = 318.13 [(**PQ2Py**+2H+OCH_3)+H]$^+$ and the m/z = 340.12 [(**PQ2Py**+2H+OCH_3)+Na]$^+$.

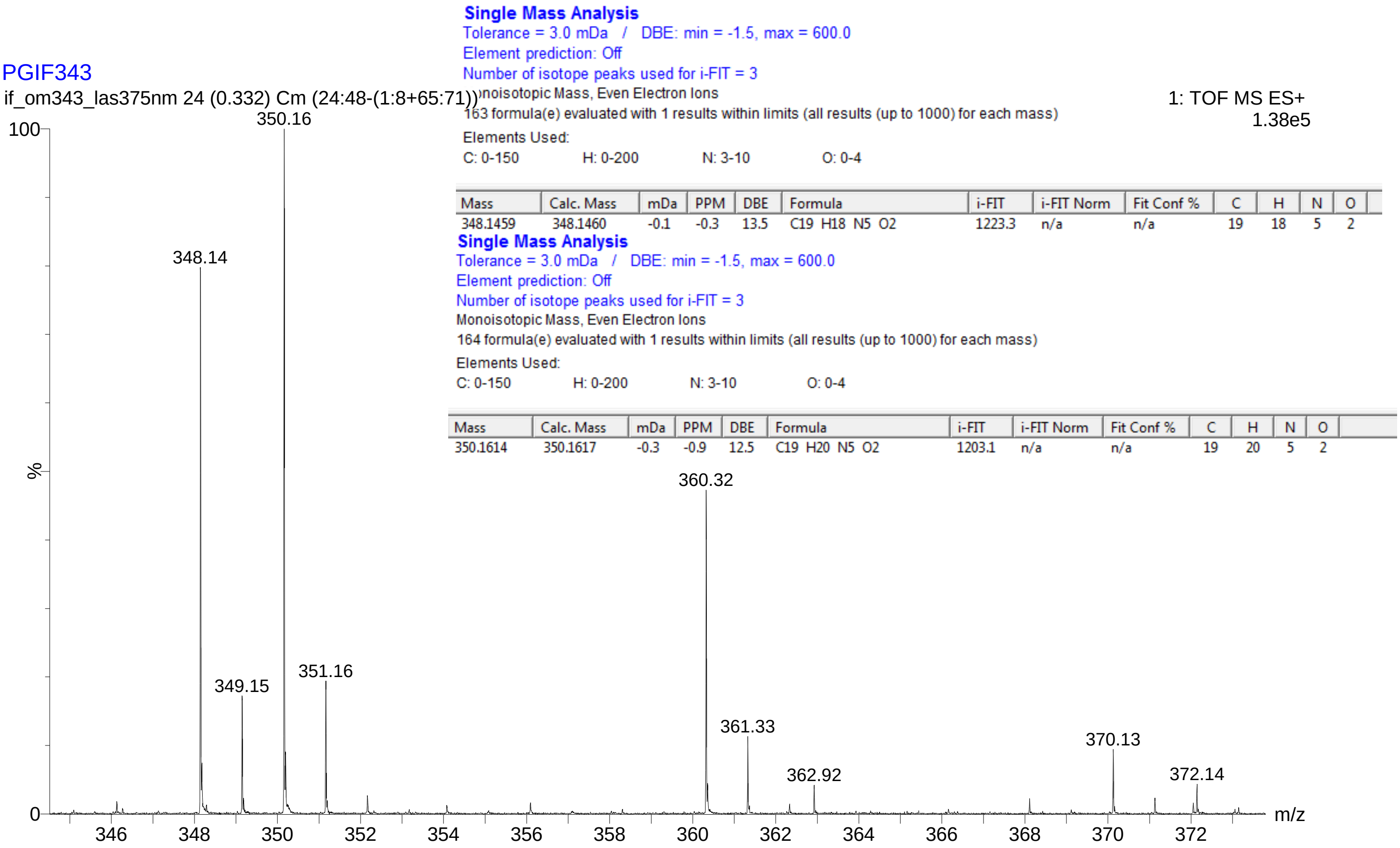

Figure S19. The extended MS of the sample **PQ2Py** after 375 nm laser irradiation and elemental prediction tables for the m/z = 348.14 [(**PQ2Py**+H+2(OCH_3)+H]$^+$ and for the m/z = 350.16 [(**PQ2Py** +2H+2(OCH_3)+H]$^+$.

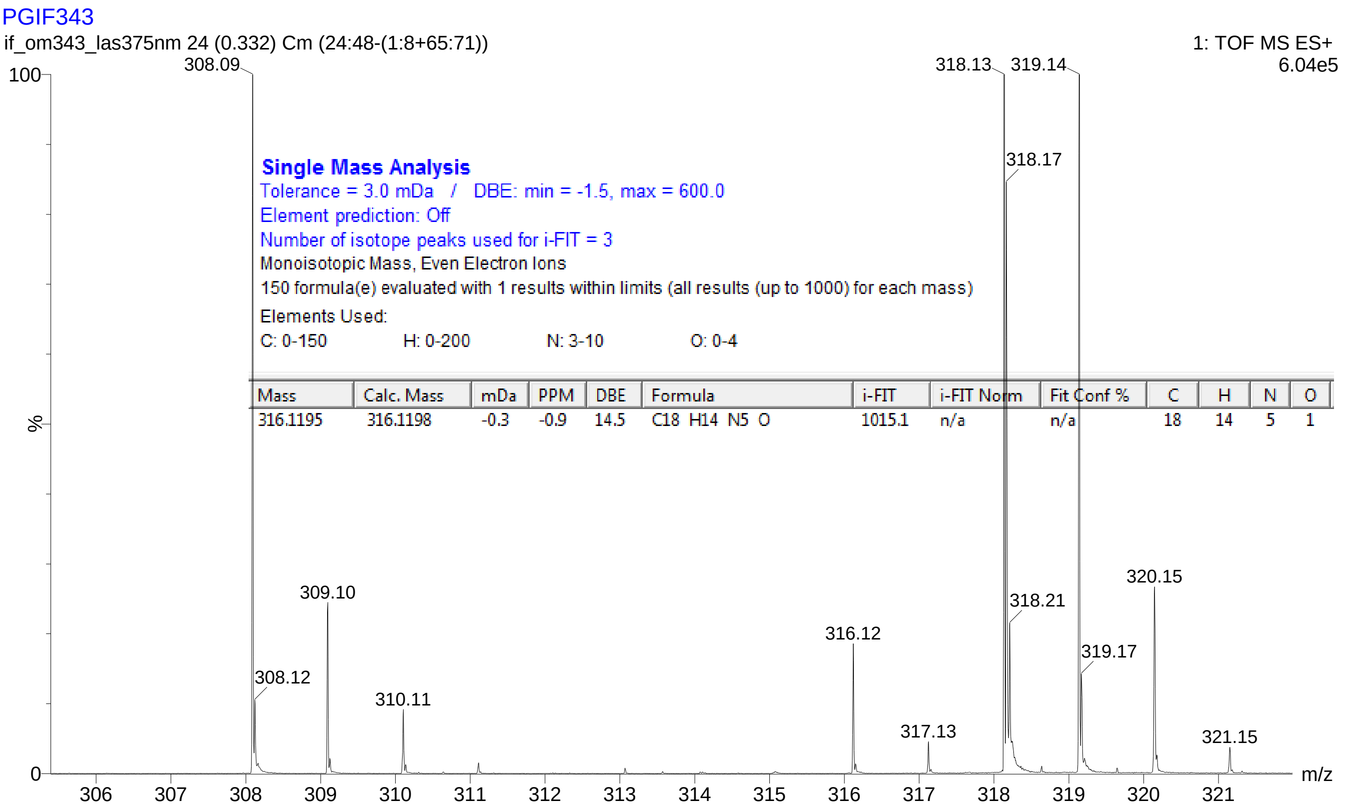

Figure S20. The extended and zoomed mass spectrum of the sample **PQ2Py** after 375 nm laser irradiation and elemental prediction table for the m/z = 316.12 [(**PQ2Py**+OCH_3)+H]$^+$.

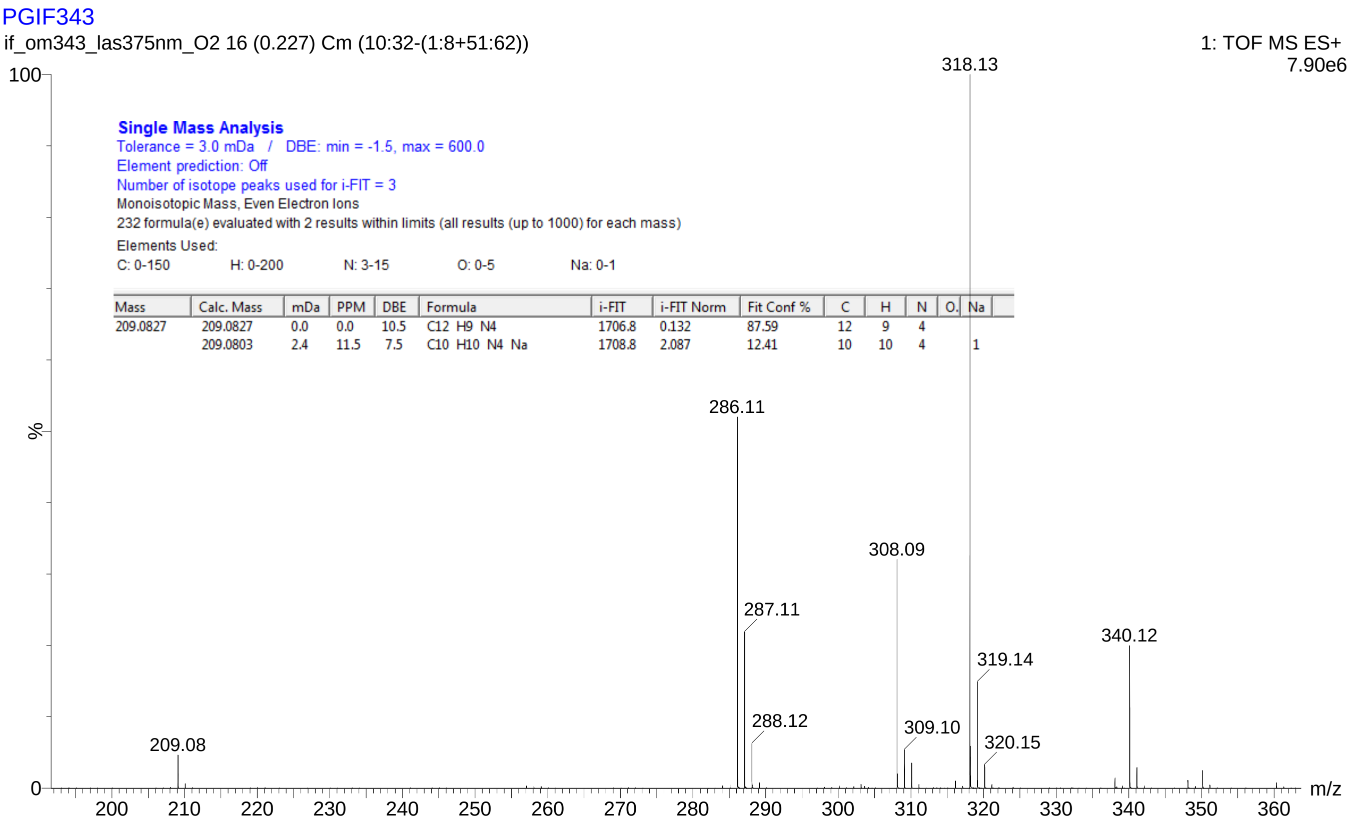

Mass	Calc. Mass	mDa	PPM	DBE	Formula	i-FIT	i-FIT Norm	Fit Conf %	C	H	N	O	Na
209.0827	209.0827	0.0	0.0	10.5	C12 H9 N4	1706.8	0.132	87.59	12	9	4		
	209.0803	2.4	11.5	7.5	C10 H10 N4 Na	1708.8	2.087	12.41	10	10	4		1

Figure S21. The mass spectrum of the sample **PQ2Py** after 375 nm laser irradiation plus additional 15 min of rest time and elemental prediction table for the m/z = 209.08 [(**PQ2Py**-Pyr)+H]$^+$. (The minus sign means the loss of the pyridine ring.)

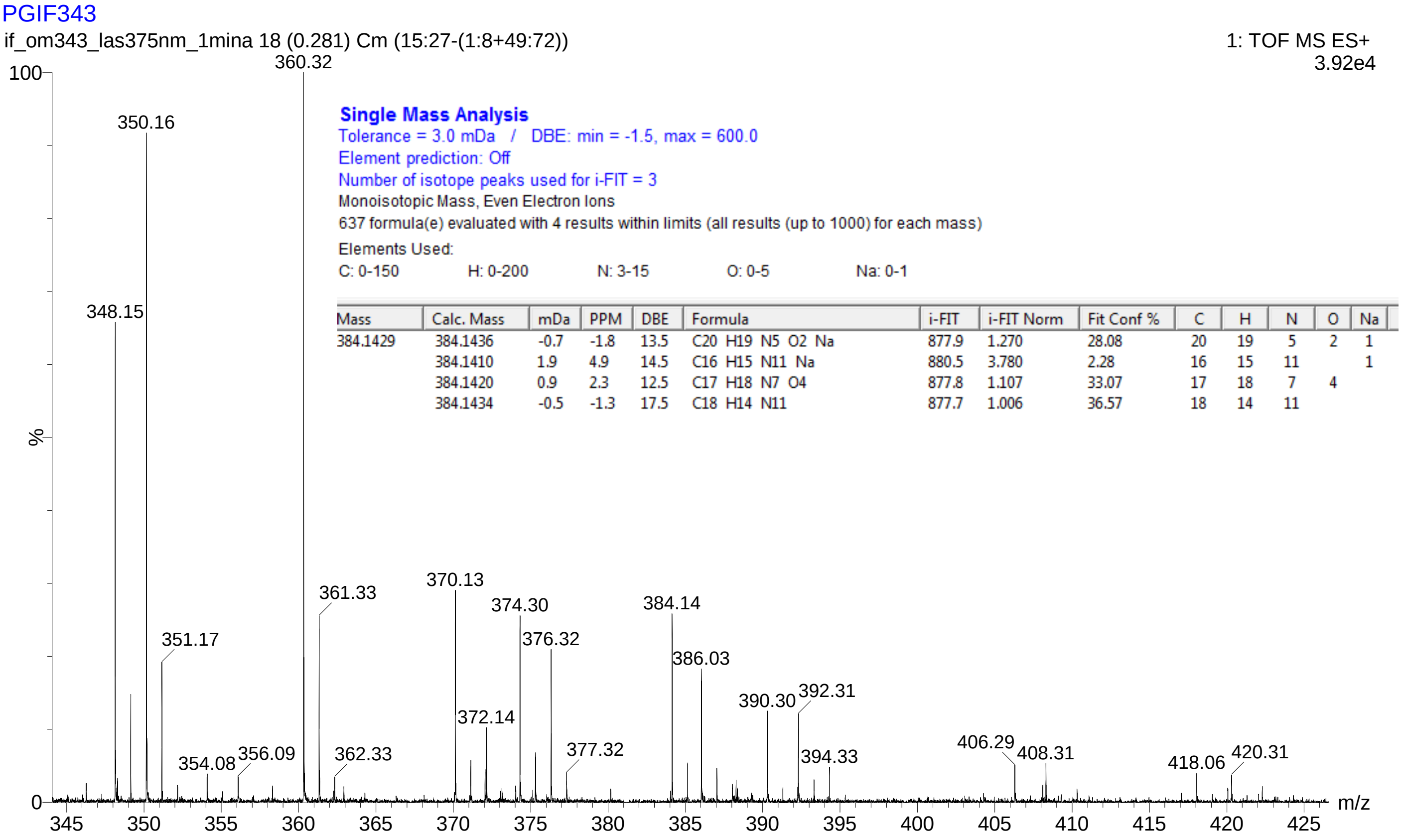

Mass	Calc. Mass	mDa	PPM	DBE	Formula	i-FIT	i-FIT Norm	Fit Conf %	C	H	N	O	Na
384.1429	384.1436	-0.7	-1.8	13.5	C20 H19 N5 O2 Na	877.9	1.270	28.08	20	19	5	2	1
	384.1410	1.9	4.9	14.5	C16 H15 N11 Na	880.5	3.780	2.28	16	15	11		1
	384.1420	0.9	2.3	12.5	C17 H18 N7 O4	877.8	1.107	33.07	17	18	7	4	
	384.1434	-0.5	-1.3	17.5	C18 H14 N11	877.7	1.006	36.57	18	14	11		

Figure S22. The extended mass spectrum of the sample **PQ2Py** in methanol after 30 minutes 375 nm laser irradiation and elemental prediction table for the m/z = 384.14 [(**PQ2Py**+2H+2(OCH_3)+CH_3)+Na]$^+$.

Table S6. The proposed structure of some photoproducts found for **PQ2Py** irradiated in methanol.

m/z	Structure
209.08	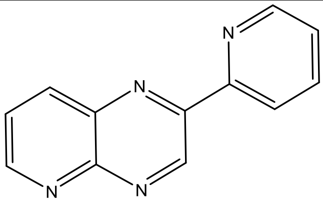<br>Chemical Formula: $C_{12}H_8N_4$<br>Exact Mass: 208,07490
315.11	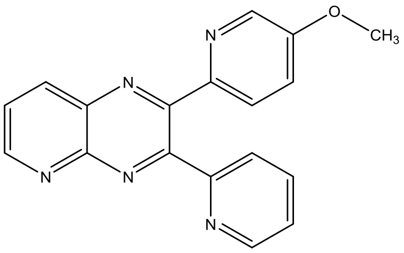<br>Chemical Formula: $C_{18}H_{13}N_5O$<br>Exact Mass: 315,11201<br>or<br>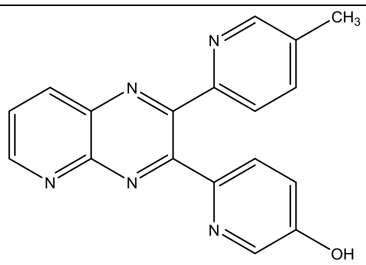<br>Chemical Formula: $C_{18}H_{13}N_5O$<br>Exact Mass: 315,11201
347.14	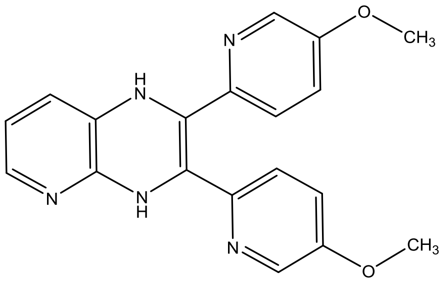<br>Chemical Formula: $C_{19}H_{17}N_5O_2$<br>Exact Mass: 347,13822<br>or<br>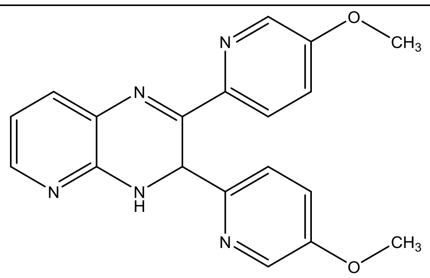<br>Chemical Formula: $C_{19}H_{17}N_5O_2$<br>Exact Mass: 347,13822
349.15	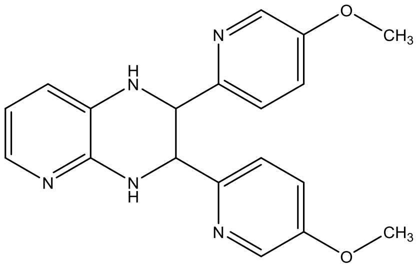<br>Chemical Formula: $C_{19}H_{19}N_5O_2$<br>Exact Mass: 349,15387
384.14<br>$[M+Na]^+$	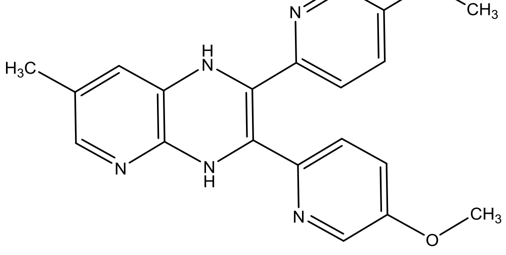<br>Chemical Formula: $C_{20}H_{19}N_5O_2$<br>Exact Mass: 361,15387

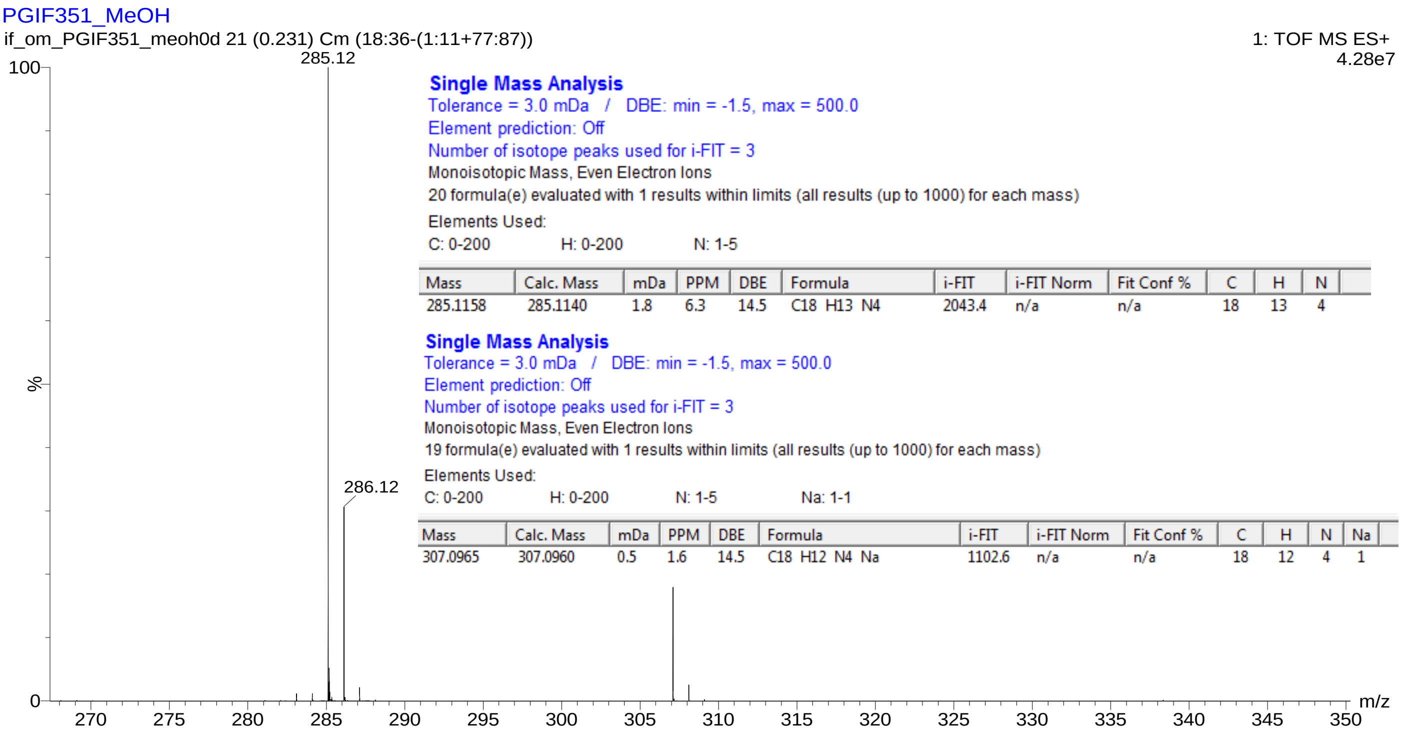

Single Mass Analysis
Tolerance = 3.0 mDa / DBE: min = -1.5, max = 500.0
Element prediction: Off
Number of isotope peaks used for i-FIT = 3
Monoisotopic Mass, Even Electron Ions
20 formula(e) evaluated with 1 results within limits (all results (up to 1000) for each mass)
Elements Used:
C: 0-200 H: 0-200 N: 1-5

Mass	Calc. Mass	mDa	PPM	DBE	Formula	i-FIT	i-FIT Norm	Fit Conf %	C	H	N
285.1158	285.1140	1.8	6.3	14.5	C18 H13 N4	2043.4	n/a	n/a	18	13	4

Single Mass Analysis
Tolerance = 3.0 mDa / DBE: min = -1.5, max = 500.0
Element prediction: Off
Number of isotope peaks used for i-FIT = 3
Monoisotopic Mass, Even Electron Ions
19 formula(e) evaluated with 1 results within limits (all results (up to 1000) for each mass)
Elements Used:
C: 0-200 H: 0-200 N: 1-5 Na: 1-1

Mass	Calc. Mass	mDa	PPM	DBE	Formula	i-FIT	i-FIT Norm	Fit Conf %	C	H	N	Na
307.0965	307.0960	0.5	1.6	14.5	C18 H12 N4 Na	1102.6	n/a	n/a	18	12	4	1

Figure S23. The extended mass spectrum of the sample **Q2Py** in methanol before 277 nm LED irradiation and elemental prediction tables for the m/z = 285.12 [**Q2Py**+H]$^+$ and for the m/z = 307.10 [**Q2Py**+Na]$^+$.

Single Mass Analysis
Tolerance = 5.0 mDa / DBE: min = -1.5, max = 500.0
Element prediction: Off
Number of isotope peaks used for i-FIT = 3
Monoisotopic Mass, Odd and Even Electron Ions
238 formula(e) evaluated with 2 results within limits (all results (up to 1000) for each mass)
Elements Used:
C: 0-100 H: 0-200 N: 1-10 O: 0-5

Mass	Calc. Mass	mDa	PPM	DBE	Formula	i-FIT	i-FIT Norm	Fit Conf %	C	H	N	O
338.3422	338.3423	-0.1	-0.3	1.5	C22 H44 N O	1324.4	0.350	70.47	22	44	1	1
	338.3409	1.3	3.8	2.0	C20 H42 N4	1325.2	1.220	29.53	20	42	4	

Single Mass Analysis
Tolerance = 5.0 mDa / DBE: min = -1.5, max = 500.0
Element prediction: Off
Number of isotope peaks used for i-FIT = 3
Monoisotopic Mass, Even Electron Ions
253 formula(e) evaluated with 3 results within limits (all results (up to 1000) for each mass)
Elements Used:
C: 0-100 H: 0-200 N: 1-10 O: 0-5

Mass	Calc. Mass	mDa	PPM	DBE	Formula	i-FIT	i-FIT Norm	Fit Conf %	C	H	N	O
360.3240	360.3266	-2.6	-7.2	4.5	C24 H42 N O	1198.3	0.585	55.73	24	42	1	1
	360.3226	1.4	3.9	0.5	C19 H42 N3 O3	1198.5	0.821	43.98	19	42	3	3
	360.3199	4.1	11.4	1.5	C15 H38 N9 O	1203.6	5.855	0.29	15	38	9	1

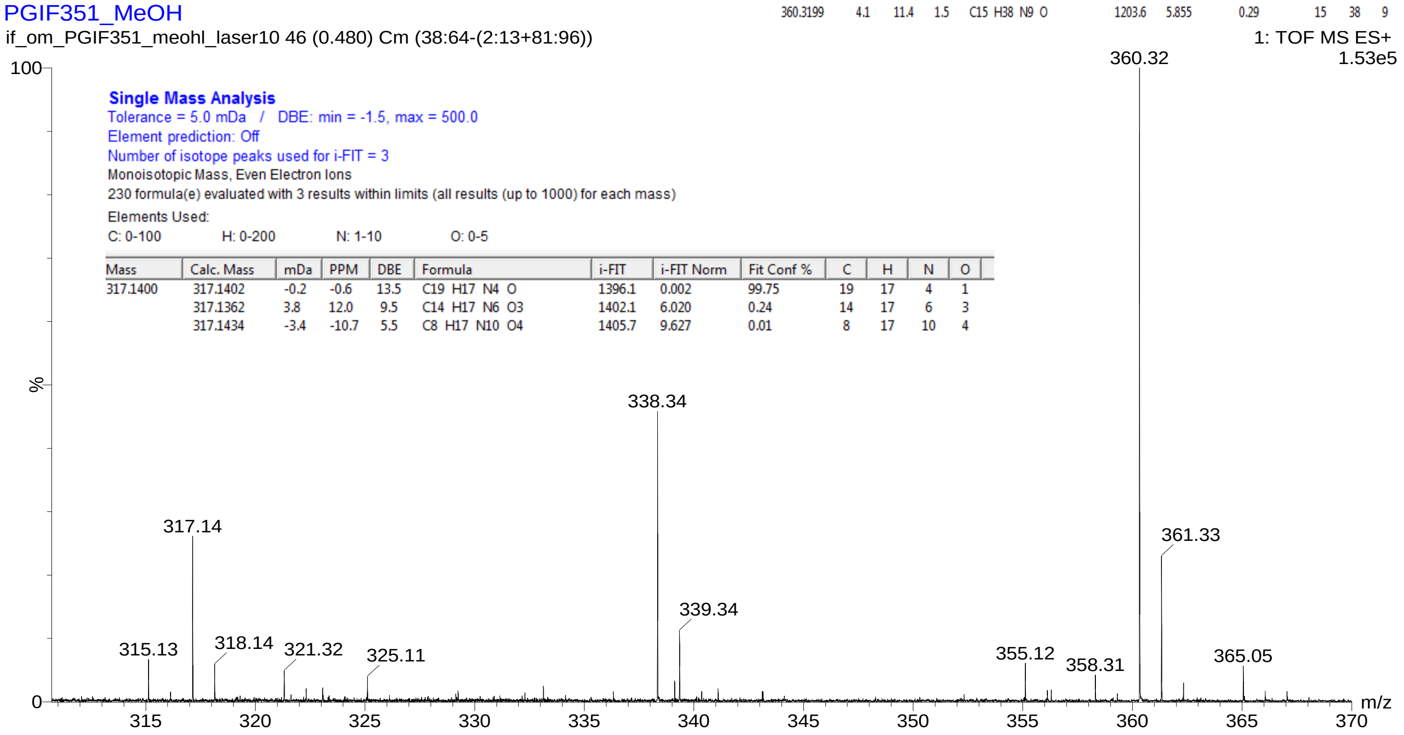

Single Mass Analysis
Tolerance = 5.0 mDa / DBE: min = -1.5, max = 500.0
Element prediction: Off
Number of isotope peaks used for i-FIT = 3
Monoisotopic Mass, Even Electron Ions
230 formula(e) evaluated with 3 results within limits (all results (up to 1000) for each mass)
Elements Used:
C: 0-100 H: 0-200 N: 1-10 O: 0-5

Mass	Calc. Mass	mDa	PPM	DBE	Formula	i-FIT	i-FIT Norm	Fit Conf %	C	H	N	O
317.1400	317.1402	-0.2	-0.6	13.5	C19 H17 N4 O	1396.1	0.002	99.75	19	17	4	1
	317.1362	3.8	12.0	9.5	C14 H17 N6 O3	1402.1	6.020	0.24	14	17	6	3
	317.1434	-3.4	-10.7	5.5	C8 H17 N10 O4	1405.7	9.627	0.01	8	17	10	4

Figure S24. The extended mass spectrum of the sample **Q2Py** in methanol after 277 nm LED irradiation and elemental prediction tables for the m/z = 317.14 [(**Q2Py**+CH_3O)+H]$^+$, m/z=338.34 and the m/z = 360.32 (not assigned).

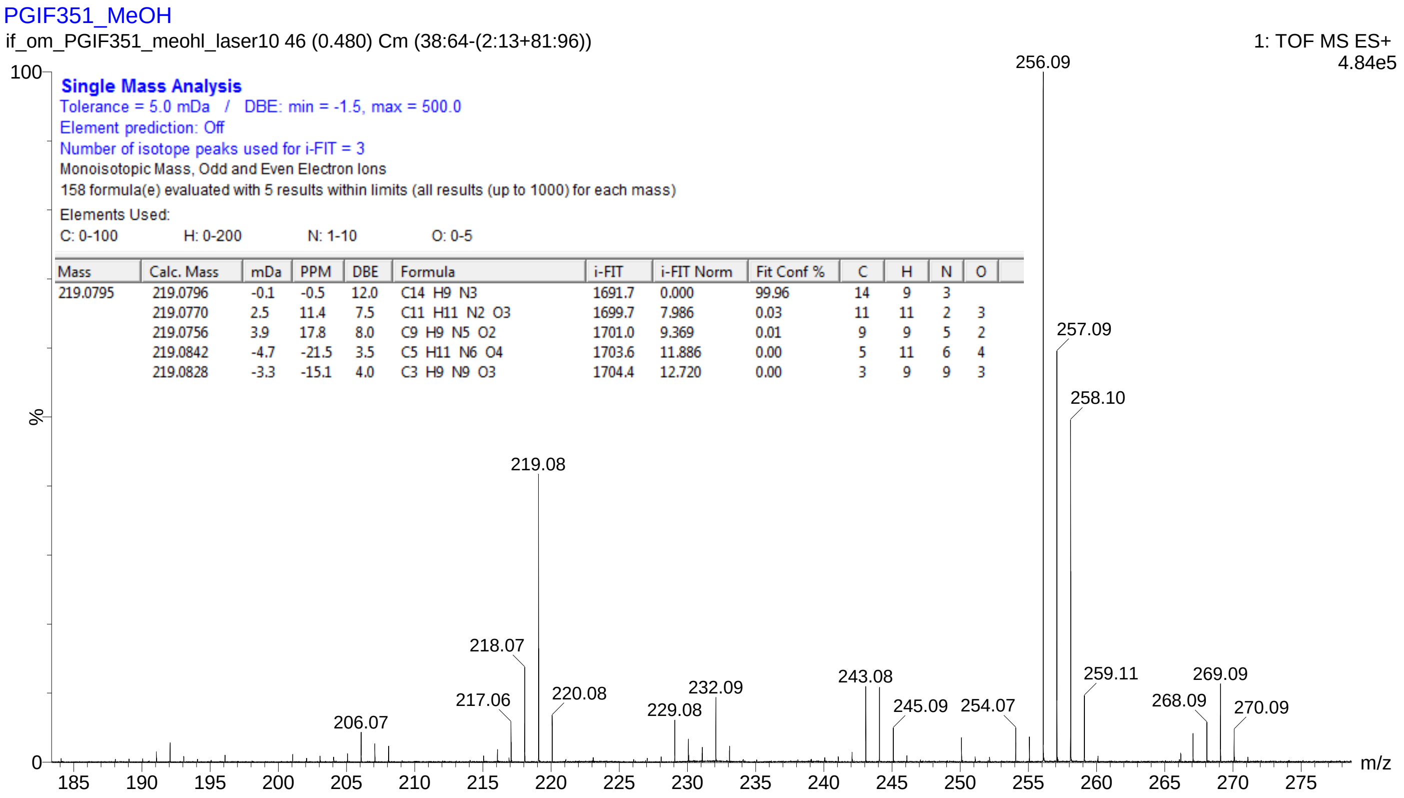

Figure S25. The extended mass spectrum of the sample **Q2Py** in methanol after 277 nm LED irradiation and elemental prediction table for the m/z = 219.08 (not assigned).

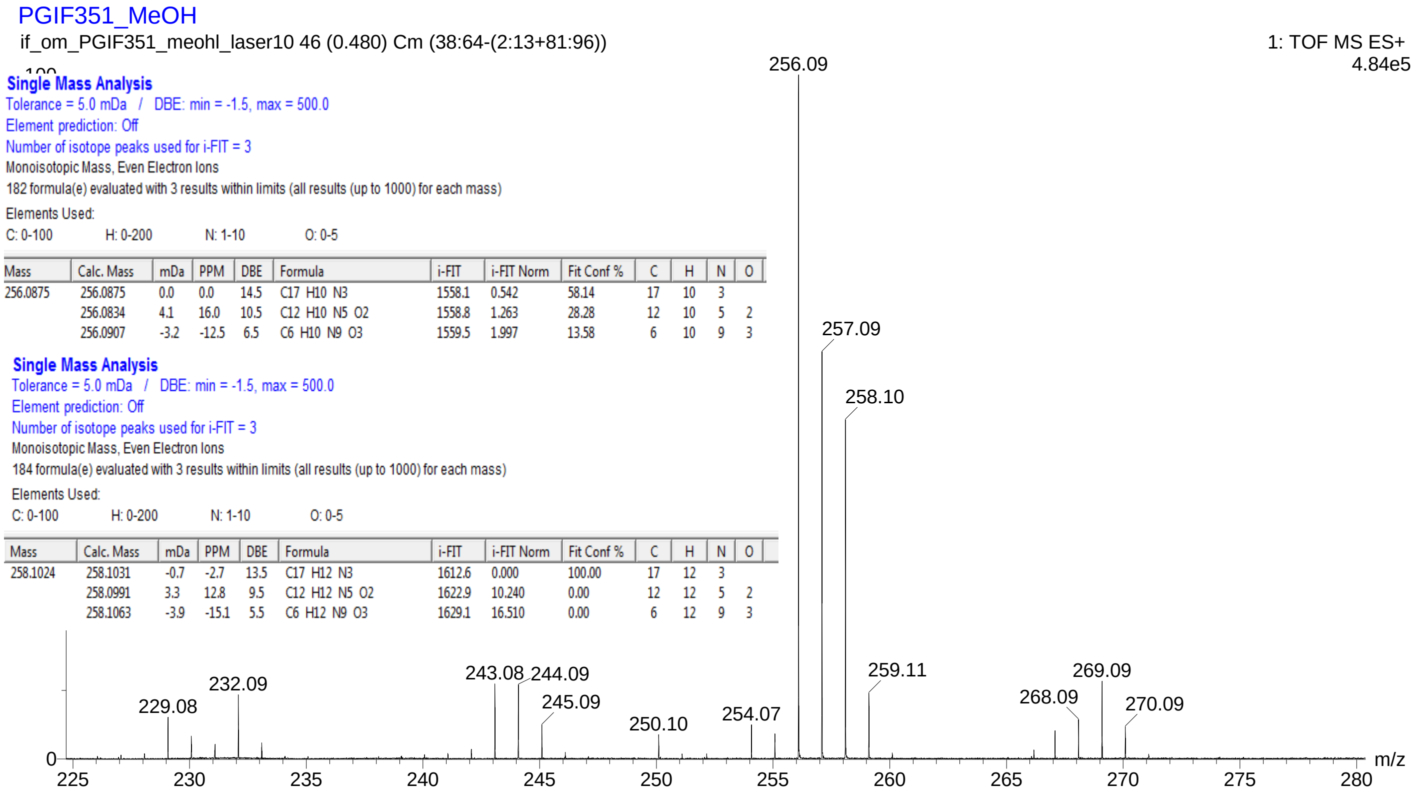

Figure S26. The extended mass spectrum of the sample **Q2Py** in methanol after 277 nm LED irradiation and elemental prediction tables for the m/z = 256.09 and the m/z = 258.10 (not assigned).
